# Cluster randomized evaluation of the Nia Project: study protocol

**DOI:** 10.1186/s12978-018-0586-4

**Published:** 2018-12-29

**Authors:** Eunice Muthengi, Karen Austrian

**Affiliations:** Population Council, P.O. Box 17643-00500, Nairobi, Kenya

**Keywords:** Adolescent girls, Randomized trial, Menstrual health, Kenya, Comprehensive sexuality education, Education, Reproductive health, Menstrual hygiene, Sanitary pads, School attendance

## Abstract

**Background:**

The onset of puberty and menarche is a specifically vulnerable time for girls, during which they begin to show interest in the opposite sex, while becoming exposed to a myriad of external pressures, including sexual coercion or harassment from boys and men, expectations to marry from their families, and the need to perform well in primary school in order to qualify for secondary school. According to several qualitative studies in Africa, such pressures are exacerbated by girls’ lack of knowledge of their bodies, their rights, and the implications of their decisions, and by their inability to manage puberty and adolescence safely and comfortably with appropriate menstrual health and hygiene management (MHM) products. The evaluation of the Nia Project is one of the first to analyze the individual and combined contributions of sanitary pads and provision of comprehensive reproductive health education on girls’ education and reproductive health outcomes.

**Methods:**

The design for the evaluation of the Nia Project is a longitudinal, cluster-randomized controlled trial consisting of a baseline survey with a cohort of Class 7 girls, a school quality survey, qualitative data collection, school attendance tracking, and an endline survey at the completion of the 18-month intervention period with the same cohort. The study involves 140 public primary schools in three rural sub-counties (Magarini, Kaloleni and Ganze) of Kilifi County in the Coastal area of Kenya. The research sample includes 3489 girls, with about 25 girls per school on average. Before program implementation, the schools were stratified by sub-county and randomized to one of four study arms (35 schools per arm): 1) control, 2) disposable sanitary pads distribution, 2) reproductive health education, and 4) sanitary pad distribution and reproductive health education.

**Discussion:**

The evidence provided will inform program investment and design, and contribute to the literature on the effect of menstrual health-based interventions on girls’ agency, safety and life outcomes.

**Trial registration:**

ISRCTN10894523. Trial Registration Date: August 22, 2017.

**Electronic supplementary material:**

The online version of this article (10.1186/s12978-018-0586-4) contains supplementary material, which is available to authorized users.

## Plain English summary

The time when adolescent girls start to experience puberty and menstruate for the first time contains many risks, including facing sexual harassment and violence from boys and men, their families pressuring them to get married and the expectation to perform well in school. Other studies in Africa confirm that these risks and pressures are made worse by girls’ lack of knowledge of their bodies and their inability to manage puberty and adolescence safely and comfortably with appropriate menstrual products. The evaluation of the Nia Project is one of the first studies to examine the effect of sanitary pads and reproductive health education on girls’ education and reproductive health.

This paper describes the design of the evaluation of the Nia Project, a program that provides girls with sanitary pads and reproductive health education. The study includes a baseline survey of about 3500 girls in Class 7, a school quality survey, qualitative data collection, school attendance tracking, and an endline survey with the same group of girls after the program is completed. The study includes 140 public primary schools in Kilifi County in the coastal area of Kenya. Schools were randomly assigned to one of four program versions: 1) control, 2) disposable sanitary pads distribution, 2) reproductive health education, and 4) disposable sanitary pad distribution and reproductive health education.

The results of this study will shape future program investment and design, and contribute to the understanding of the effects of menstrual health-based interventions on girls’ agency, safety and life outcomes.

## Background

The onset of puberty and menarche is a specifically vulnerable time for girls, during which they begin to show interest in the opposite sex, while becoming exposed to a myriad of external pressures, including sexual coercion or harassment from boys and men, expectations to marry from their families, and the need to perform well in primary school in order to qualify for secondary school [1]. According to several qualitative studies in Africa, such pressures are exacerbated by girls’ lack of knowledge of their bodies, their rights, and the implications of their decisions, and by their inability to manage puberty and adolescence safely and comfortably with appropriate menstrual health and hygiene management (MHM) products [2–8].

Qualitative studies conducted in Kenya have identified several challenges girls face in managing their menstruation, including lack of access to menstrual products and lack of accurate information about menstruation. In a study conducted in Nyanza Province in Western Kenya, girls who participated in focus groups and in-depth interviews reported poverty as a main barrier for proper menstruation management. When families are unable to prioritize buying sanitary pads, girls resort to using cloths, mattress pieces and other materials, but they describe these alternatives as ineffective in preventing leaking of blood onto their clothing [9]. In Siaya County, also in Western Kenya, a qualitative study of young adolescent girls also highlighted the inadequacy of alternatives such as cloths, blankets and mattress pieces and their preference for sanitary pads. Although the most common source of sanitary pads was mothers, some girls reported receiving money from boyfriends to buy pads, often with the expectation that they will have sex with them in return [3].

Both studies found that girls commonly had little or no prior knowledge of menstruation because traditional systems for passing on this knowledge were no longer functional and many mothers did not prepare their daughters for menstruation [3, 9]. Data from focus group discussions in an informal settlement in Nairobi also showed that mothers’ limited knowledge, embarrassment and cultural taboos prevented them from providing their daughters with accurate information on reproductive health, including menstruation [10]. Furthermore, teachers in Siaya most often stated that it was not their role to provide girls with information on menstruation [9].

While several programs have previously been developed to address girls’ MHM needs in Kenya, as well as globally, few have been rigorously evaluated, and where evidence does exist, the results have been mixed. The government of Kenya, in response to girls’ MHM needs has committed to sanitary pad distribution in government schools [11], however, evaluations have shown that the supply chains of sanitary pads to government schools were not reliable, and girls were not assured of equitable pad provision [12].

A 2013 systematic review of the literature identified 14 studies that examined health outcomes such as reproductive tract infections and 11 articles that examined psycho-social outcomes of menstrual hygiene [13]. The authors concluded that while there is some evidence on the impact of MHM on psycho-social outcomes, the impact on health outcomes, specifically reproductive tract infections, is unclear. Furthermore, there is no quantitative evidence on the effects of MHM on reducing school absenteeism. They also noted the lack of rigorous studies showing the impact of MHM on girls’ general health and well-being.

In February 2016, Hennegan and Montgomery published an MHM related systematic review that identified eight studies that fulfilled their criteria: individual or cluster randomized controlled trials (RCT) and non-randomized controlled trials [14]. The purpose of the review was to assess the risk of bias in these studies and synthesize the evidence on the effects of MHM interventions on educational and psychosocial outcomes for women and girls in low and middle income countries. The authors outlined two dominant types of MHM intervention approaches: products, or the provision of physical objects useful for MHM, such as menstrual cups or sanitary pads; and empowerment, or the provision of human and social capital through education and non-tangible benefits. The authors found considerable risk of bias in these studies, mainly selection bias, performance bias, attrition bias, and reporting bias. They were unable to synthesize most results due to differences in interventions and methods and, therefore, concluded that while there are some indications of positive results, insufficient evidence exists for the effectiveness of MHM interventions.

Only one study identified in the review examined the effect of a combined empowerment and products intervention on schooling attendance. The study was conducted in Ghana using a non-randomized cluster-control trial design, with a sample of 120 girls between the ages of 12 and 18 [15]. The three interventions implemented were: 1) provision of pads with puberty education, 2) puberty education alone, and 3) control group (no intervention). School attendance was measured using official school records, triangulated with researcher visits. At the end of the 5-month study period, study investigators observed statistically significant improvements in attendance for both treatment arms, by approximately 5 to 6 days per 65-day term. However, the main limitation of the study was the small sample size and non-randomized design. Dolan and colleagues [16] further reported a significant improvement over time in confidence regarding menstruation management in the study arm that included provision of pads. No statistically significant change in confidence (as measured by shame, self-confidence and insecurity) was observed in the education-only arm. However, as noted by Hennegan and Montgomery [14], these findings should be interpreted with caution because confidence was not measured in the control group, and girls differed on this indicator across sites at baseline.

Due to the lack of clear evidence on the impact of MHM interventions on girls’ education and health, ZanaAfrica received funding in 2015, from the Bill & Melinda Gates Foundation, to implement a holistic solution combining sanitary pads and reproductive health education, including multi-media health education resources. The package of interventions was branded as ‘The Nia Project’, from the Swahili word ‘nia’ which means ‘purpose.’ ZanaAfrica is partnering with Population Council to rigorously evaluate the Nia Project interventions among adolescent girls in Kilifi County, which is located in the Coastal region of Kenya. Kilifi was identified as the study site based on a review of indicators related to education and reproductive health; for example, the low transition rate from primary to secondary school, recorded as 40% in 2010, compared to the national rate of 72% [17]. In addition, Kilifi was ranked 36 out of 47 counties in regards to the Net Enrollment Rate for secondary school for boys and girls, which was only 26% in 2014 [18]. According to the 2014 Kenya Demographic and Health Survey [19], approximately 22% of girls between the ages of 15 and 19 in Kilifi County had begun childbearing, compared to the national average of 18%.

Using a randomized controlled trial (RCT) research design, this evaluation is one of the first to analyze the individual and combined contributions of sanitary pads and provision of comprehensive reproductive health education on girls’ education and reproductive health outcomes. The evidence provided will inform program investment and design, and contribute to the literature on the effect of menstrual health-based interventions on girls’ agency, safety and life outcomes.

The main objective of the research is to answer the following research question: What is the effect of an MHM intervention combining empowerment (reproductive health education) and products (sanitary pads) approaches on girls’ well-being (social, and personal competencies) and education, versus empowerment or products alone? The study was specifically designed to address key limitations of previous studies, which include small sample sizes, inability to determine causation, non-random assignment to study arms, and short follow-up periods.

This paper describes the intervention and research design of the Nia Project.

## Methods/design

The Nia Project interventions are implemented by ZanaAfrica and Plan International, Kenya. Girls enrolled in Class 7 at the start of the 2017 school year are the beneficiaries, receiving interventions over a period of 5 school terms, from May 2017 to December 2018. The Nia Project includes two intervention components: 1) provision of Nia brand disposable sanitary pads, and 2) reproductive health education, including menstrual hygiene management as an extracurricular program for girls enrolled in school.

### Sanitary pads

In 2011, the Kenyan government, through the Ministry of Education, Science and Technology, initiated the Schools’ National Sanitary Towels program to increase girls’ school attendance and participation. The program includes the provision of sanitary pads to school girls and the training of teachers on hygienic usage and disposal of pads (UNESCO Booklet 9 – puberty education and MHM). However, results from a formative assessment conducted in Kilifi to inform the design of the Nia Project indicated that some schools receive inadequate supplies to meet the needs of all female students throughout the year.

For the Nia Project, each girl receives one packet of 10 disposable sanitary pads each month of ZanaAfrica’s Nia Teen brand, for the entire project period. To avoid duplication, schools agreed to only distribute government-issued pads to girls who are not part of the Nia Project, while ZanaAfrica distributes Nia brand disposable sanitary pads to Class 7 girls. Lack of underwear was identified as a barrier to pad usage during the formative assessment. Therefore, project beneficiaries also receive two pairs of underwear at the start of the intervention, and an additional pair at the end of each subsequent term.

### Reproductive health education

Kenyan primary education includes a National Life Skills Education curriculum, which focuses on communication, negotiation and decision-making skills. The curriculum is not comprehensive and has been assessed as being weak in regards to topics such as: power in relationships, reproduction, sexuality and sexual behavior, HIV and sexually transmitted infections (STIs), contraceptives and gender and human rights [20]. Kenya’s 2015 National Adolescent Sexual and Reproductive Health Policy provides the basis for the provision of age-appropriate comprehensive sexuality education (CSE) for in-school adolescents.

The Nia Project reproductive health education intervention is comprised of facilitated health education sessions (FHE) and a health magazine developed by ZanaAfrica. Both components are based on the UNESCO International Technical Guidance on Sexuality Education [21] and designed to incorporate recent evidence on the importance of addressing gender and power in sexuality and HIV education programs [22, 23]. *Nia Yetu,* the curriculum for the FHE sessions, was developed by ZanaAfrica [24] with many elements adapted from the Tuko Pamoja: Adolescent Reproductive Health and Life Skills Curriculum [25] developed by PATH and Population Council as part of the Kenya Adolescent Reproductive Health Program (KARHP). The 25-session curriculum (see Table 1) is delivered by trained facilitators during weekly girls-only health clubs in 2017, and bi-monthly health clubs in 2018, held during time allocated for extra-curricular activities in school.

**Table 1:** Nia Yetu Curriculum Topics

Module:	Sessions:	Duration:
1. Welcome to Puberty	1. Getting Started, Values	1:30
	2. Setting Goals	1:05
	3. Adolescence and Puberty	1:35
	4. Menstruation	1:35
	5. Menstrual Health Management	1:30
2. Gender	1. Male and Female Reproductive Systems	1:20
	2. Self Esteem	1:20
	3. Communication	1:20
	4. Introduction to Gender	1:20
	5. Gender Stereotypes	1:20
3. Gender, Power, and Rights	1. Human Rights	1:35
	2. Power Dynamics	1:35
	3. Sexual Violence and Exploitation	1:35
	4. How to Report and Avoid Cases of Sexual Violence	1:10
	5. Being Assertive	1:35
	6. Decision Making	1:10
4. Gender, Power, and Rights	1. Healthy Relationships	1:10
	2. Romantic Relationships	1:10
	3. Sexuality and Behavior	1:15
	4. Teenage Pregnancy	1:15
	5. Sexually Transmitted Infections (STIs) and HIV	1:35
5. Being True to Yourself	1. Resisting Peer Pressure	1:20
	2. Drug Use and Abuse	1:30
	3. Managing Stress, Anger, and Conflict	1:30
	4. Program Wrap Up	1:30

ZanaAfrica’s health magazine, *Nia Teen,* provides content that girls can refer to. It is designed to appeal to adolescent girls and conveys core reproductive health messaging through storytelling using aspirational personal stories, a relatable comic-style story, and activities. *Nia Teen* magazine is distributed at the start of each term for the five-term period. Each issue corresponds directly to the topic covered in each module of the Nia Yetu curriculum that term.

### Cluster randomized study design

**Fig. 1:**
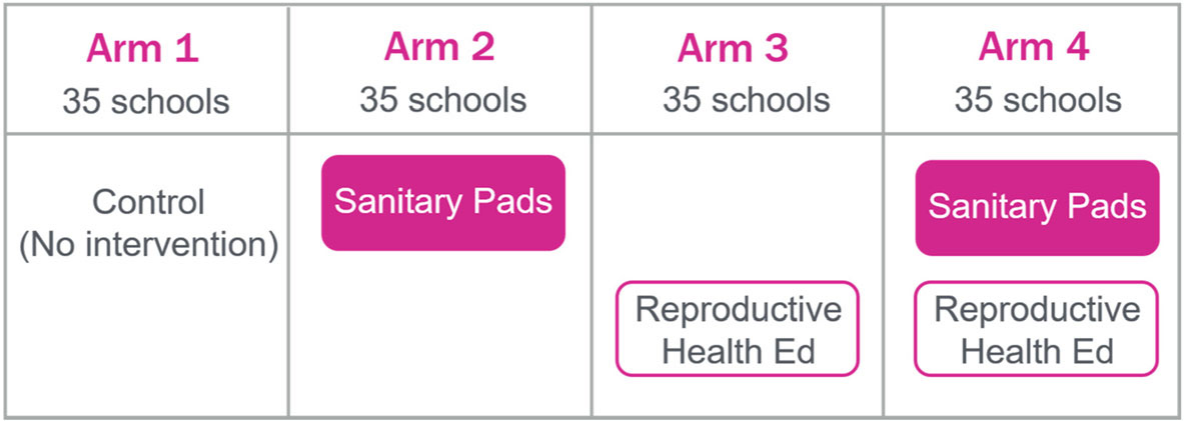
Nia Project study design

The design for the evaluation of the Nia Project is a longitudinal, cluster-randomized controlled trial consisting of a baseline survey with a cohort of Class 7 girls, a school quality survey, qualitative data collection, school attendance tracking, and an endline survey at the completion of the 18-month intervention period with the same cohort. The study involves 140 public primary schools (35 schools per arm) in three rural sub-counties (Magarini, Kaloleni and Ganze) of Kilifi County in the Coastal area of Kenya. Before program implementation, the schools were stratified by sub-county and randomly assigned to one of the following four study arms (See Fig. 1):

1. Control group;
2. Sanitary pad distribution;
3. Reproductive health education; or
4. Sanitary pad distribution + reproductive health education.

### Study sites

The sub-counties and schools were selected in collaboration with the Kilifi County Department of Education, Ministry of Education, Science and Technology. Within the three sub-counties (Magarini, Kaloleni and Ganze), all schools with 25 or more girls in Class 7 were eligible for the study. A total of 215 schools were mapped, and a 1000 m buffer was created around each school. For schools with overlapping boundaries, one school was randomly selected. This resulted in a list of 173 schools with at least 25 girls in Class 6 or in Class 7 in 2016. A listing exercise was conducted to verify enrollment and school type in the first quarter of 2017. Based on this data, 25 schools were excluded because they did not meet the minimum criteria of 25 girls in Class 7, and an additional seven were excluded because they were determined to be boarding schools. Subsequently, one school in Magarini was excluded after it was closed indefinitely by the Department of Education during baseline data collection. This resulted in 140 schools – 44 in Magarini, 50 in Kaloleni and 46 in Ganze.

In schools with 25 girls in Class 7, all girls were included in the research sample. In schools with a larger number of girls, 25 girls were randomly selected for the research sample and five additional girls were selected as alternates. Of the expected 3500 girls, a total of 3489 girls were interviewed as part of the baseline survey. All Class 7 girls, including those who were not in the research sample, are eligible to receive interventions. At the completion of data collection in each sub-county, stakeholder meetings were held and schools within that sub-county were randomly assigned to one of the four study arms through a public lottery.

### Sample size calculations

The sample size was calculated based on power calculations to determine the number of clusters (i.e., schools) and the number of girls needed in each cluster to observe the desired change in the mean days of school missed, according to differences detected in previous studies conducted in Kenya and Ghana [15, 26]. The estimate of the intra-cluster correlation (ICC) and the baseline estimates for childbearing and education were based on a previous menstrual health study conducted in Kenya by Wilson and colleagues [26]. With a sample size of 35 clusters per arm and 20 girls per cluster at endline (25 girls per cluster at baseline, assuming a loss of 20% by endline), the minimum detectable difference between study arms, with power of 0.80 and a significance level of 0.05 is:

- 1.18 mean days of school missed over a 4-week period, with an ICC of 0.173 and a standard deviation of 3.57; and
- a 13% increase in correct knowledge and attitudes on a reproductive health score from 75 to 85% in intervention arms with no change assumed in the control arm.

### Research instruments

The instruments were developed by the study investigators and reviewed by the project’s Research Advisory Committee (RAC) (Additional file 1). The school quality tool is based on the Kenya Ministry of Education, Science and Technology school census tool for their Education Management and Information System. It includes information on school enrollment, number and education level of teachers, access to water and sanitation facilities, provision of sanitary pads, etc. These quality measures will later be accounted for in the analysis as a mediating factor in the relationship between the intervention and schooling outcomes.

A quantitative survey for girls was administered at baseline in the first quarter of 2017, and a similar survey will be administered after 18-21 months (Additional file 2). The survey covered topics such as: household socio-demographic characteristics, education participation, gender norms, social assets and networks, self-efficacy, locus-of-control, savings and livelihoods, marital and child-bearing experiences, menstruation experiences, experience of physical and sexual harassment and violence, reproductive health knowledge, HIV and AIDS risk perception, sexual behavior, comprehension in Swahili and English, mathematics assessments, and cognitive testing. The endline survey will include additional questions to measure exposure to interventions. The surveys were translated into Swahili and pilot-tested prior to the start of data collection and revised based on feedback from interviewers before data collection begins.

### Study outcomes and hypotheses

The overall goal of the project is to determine the impact of Menstrual Health Management (MHM) interventions on girls’ well-being and education. The main objective of this research will be to determine what is the effect of an MHM intervention combining empowerment (reproductive health education) and products (sanitary pads) approaches on girls’ well-being and education, versus empowerment or products alone. The outcomes of interest include both short-term indicators of education and reproductive health and well-being, as well as long-term indicators, as shown in Table 2.

**Table 2:** Key Study Indicators

Key Indicators	Well-Being	Education
Short-term	- Sexual and reproductive health rights (SRHR) knowledge and attitudes - Menstrual health knowledge and attitudes - Gender norms - Self-efficacy	-School attendance -School participation/engagement
Long-term	- Experience of unwanted sex - Timing of first sex - Timing of pregnancy	-School retention -Performance in school

**Fig. 2:**
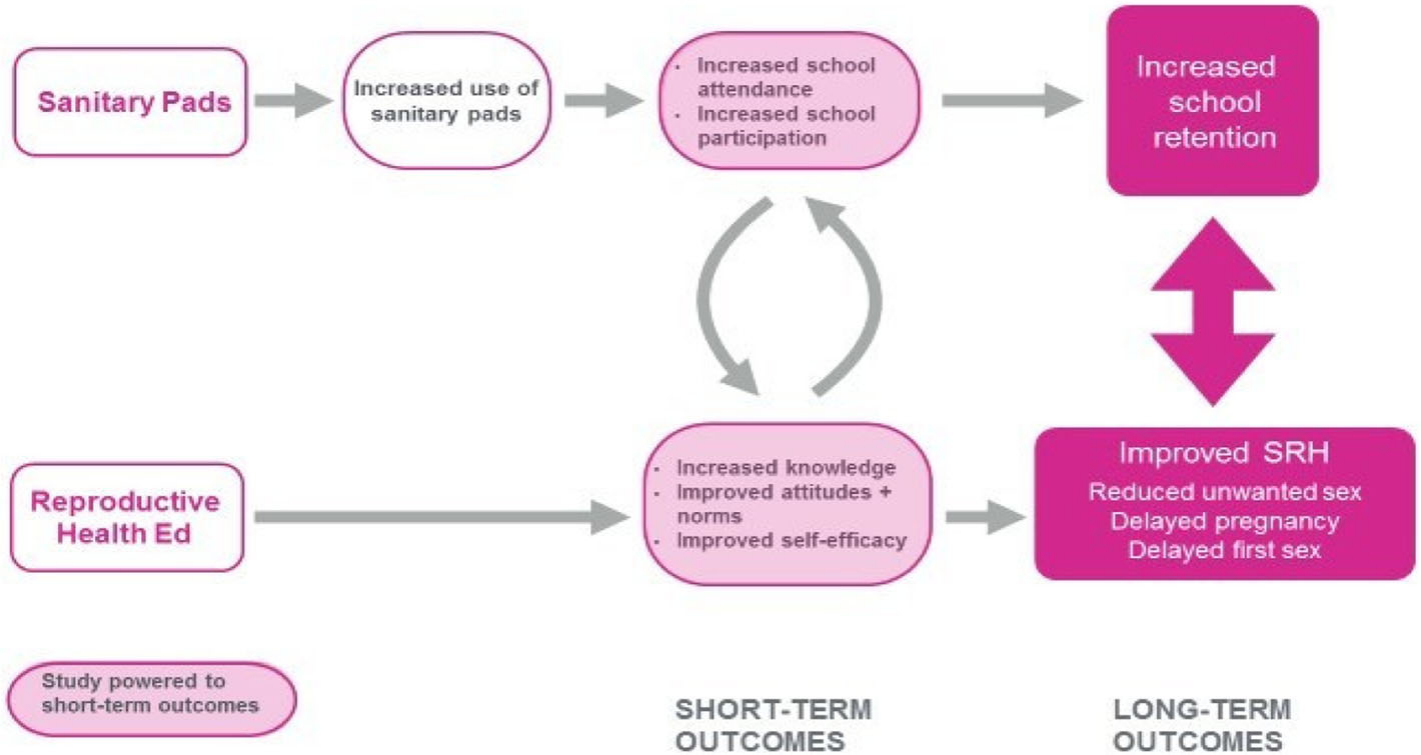
Nia Project Theory of Change

As shown in Fig. 2, the project’s theory of change hypothesizes that the Nia brand disposable sanitary pad distribution will increase the use of pads to manage menstruation, which will lead to an increase in two short-term education indicators: 1) school attendance, and 2) participation / engagement in class. The bottom panel of the figure shows the hypothesized impact of the reproductive health education component on key short-term indicators of girls’ social and personal competencies: 1) sexual and reproductive health and rights (SRHR) knowledge and attitudes, including menstrual health knowledge and attitudes, 2) gender norms, and 3) self-efficacy. This conceptual framework is based on Lloyd’s [27] typology of competencies (e.g., knowledge, skills attitudes and values) that basic education should incorporate in order to empower adolescent girls. According to this typology, social competencies include pro-social values (i.e. attitudes that are positive and/or beneficial for others and society), respect for human rights and gender consciousness, while personal competencies include self-esteem, reproductive health knowledge and management skills and self-protective skills.

The combination of the two intervention components is hypothesized to synergistically increase the effect size for all outcomes, which could potentially affect longer-term outcomes, such as school retention, reduced unwanted sex, delayed pregnancy and delayed first sex. While this study is powered to detect changes in the short-term indicators, additional analyses will be conducted to determine whether girls observed short-term improvements are associated with longer-term impacts.

### Analysis plan

The program evaluation of the Nia Project will examine the effect of the average treatment across study arms while identifying the causal mechanisms driving that impact. Using intent-to-treat analysis, all girls in the original sample randomized to study arms will remain part of the study despite non-compliance, protocol deviations, withdrawal, or anything else that happens after randomization. Econometric estimation techniques such as fixed effects estimation will be applied to control for unobserved time invariant individual characteristics. The evaluation will compare the average increase in outcomes of girls in intervention arms to the average increase in outcomes of girls in the control arm. Additional hypotheses will be tested using difference in difference analysis to understand both causal mechanisms among girls who received the interventions and the potential effect of improvements in short-term indicators on longer-term outcomes. The rich dataset will provide an extensive list of variables that can be adjusted for directly in the analysis. The results will be triangulated using qualitative data.

To examine balance across study arms following randomization, multinomial logistic regression models were estimated using each dataset to test for joint orthogonality for a selected list of indicators, comparing each treatment group with the control group. For the school quality dataset, the variables included were: school performance by gender, number of toilets, distribution of pads and availability of pads in stock. The results showed that there were jointly no significant differences between study arms (*p* = .60). For the girls’ dataset, indicators included were: ever repeated a grade, age at starting school, self-efficacy scale, decision-making scale, gender-norms scale, reproductive health knowledge score, ever menstruated, justification of violence, experience of symptoms of reproductive health infections and ever had sex. The results showed that there were jointly no significant differences between study arms (*p* = 0.14).

### Ethical considerations

The study protocol was approved by the Population Council Institutional Review Board and the AMREF Ethical and Scientific Review Committee. In addition, the protocol was reviewed by the Kenyan National Commission for Science, Technology and Innovation to obtain research permits for study investigators.

## Discussion

While there are a multitude of programs focused on distribution of sanitary pads, including government programs and others run by non-governmental organizations or community based organizations, and there is no challenge to girls’ right to manage their menstruation comfortably and with dignity, there is a gap in the literature on the efficacy of sanitary pad distribution as an intervention to improve schooling and reproductive health outcomes for school going adolescent girls. Furthermore, there is a lack of evidence on the synergistic effects of combining sanitary pad distribution with comprehensive reproductive health education [13, 14]. The evaluation of the Nia Project has the potential to fill those gaps and is poised as one of the first rigorous, randomized controlled trials to explore the role of sanitary pad distribution and reproductive health education – individually and in combination – to improve girls’ educational and SRH outcomes. The findings of this study will make a critical contribution to filling an evidence gap in the fields of MHM and girls education and, in so doing, has the ability to guide education and health policy in this area in Kenya and in the region to ensure that policies linking MHM with education and other health outcomes are evidence-based and likely to achieved the outcomes desired.

## Additional files

Additional file 1: Nia School Quality Tool (PDF 384 kb)

Additional file 2: Nia Baseline Tool (PDF 1134 kb)

## Abbreviations

AIDS: Acquired Immune Deficiency Syndrome
CSE: Comprehensive Sexuality Education
HIV: Human Immunodeficiency Virus
ICC: Intra-Cluster Correlation
KARHP: Kenya Adolescent Reproductive Health Program
MHM: Menstrual Health and Hygiene Management
RAC: Research Advisory Committee
RCT: Randomized Controlled Trial
SRHR: Sexual and Reproductive Health Rights
STI: Sexually Transmitted Infection
