# Supplementary material for: Cluster randomized evaluation of the Nia Project: study protocol
Source: Reprod Health. 2018 Dec 29;15:218. doi: 10.1186/s12978-018-0586-4 (PMC6310925; doi:10.1186/s12978-018-0586-4)
Supplement: Supplementary file 1 — Nia School Quality Tool (PDF 384 kb) [file 12978_2018_586_MOESM1_ESM.pdf]

SCHOOL QUALITY SURVEY

Primary Education

School Year 20…........

School Name: …………………………………………………….

Address:……………………………………………………….

School Year of Registration:..…………………………………

Registered with: MOEST☐ Other Ministry (Specify) ☐ ________________

Registration Number: |___|___||___|___||___|___||___|___||___|___||___|___|

TSC Code: |___|___||___|___||___|___||___||___||___|

KNEC Code: |___|___||___|___||___|___|

School Management Committee: Yes☐ No☐

County ……….…………Sub-County………………………….

Zone ……………………………..

Constituency………..………………………

Ward …………………………………….

Location: Rural☐ Urban☐

Does the school have a feeding program? Yes ☐ No☐

WF Program: Yes☐ No☐

Feed the Children: Yes☐ No☐

School Program: Yes☐ No☐

School Status : Public☐ Private/Non-public ☐

School Category : Ordinary☐ Integrated☐ Mobile☐ Special Needs☐

School Type : Boys☐ Girls☐ Mixed☐

Accommodation: Day☐ Boarding☐Day &Boarding☐

For Day &Boarding, how many pupils board? Boys…… Girls: ……

Enrolment, Repeaters, Streams, Special Needs And OVCs By Class

		Primary								
		Class 1	*Class 2*	*Class 3*	*Class 4*	*Class 5*	*Class 6*	*Class 7*	*Class 8*	*Total*
Current year Enrollment	*Boys*									
	Girls									
14 years & above	*Boys*									
	Girls									
Repeaters	*Boys*									
	Girls									
Number of streams	*Total*									
Pupils with no desks	*Total*									
No. of Special Needs	*Total*									
No. of OVCs	*Total*									

School Personnel

	Number of TSC Teachers by Qualification					Number Of Teachers Employed by SMC/BOG/ PTA	Number of non-Teaching			Labors
	With university degree	With diploma	With P1 Certificate	Others	Total		With diploma and above	Others	Total	Total number
Male										
Femal										

Exam Results, Textbooks, Facilities, School Feeding and Other Amenities

Last Year KCPE Examination Details		
	Boys	Girls
Number registered		
Mean score (100-500)		
Pupils admitted to join		

Number Of Textbooks By Subject					
	Maths	English	Kiswahili	Science	Social Studies
Lower					
Upper					

Number of class 7 pupils on school feeding:
Boys__________ Girls________

Is your school connected to electricity? *Yes*☐*No*☐<br>If No, source of lighting: No light☐ Other: ……………………………	School's water source for drinking: Tap☐ River☐ Rain☐ Borehole☐ Water Truck/Bowser☐ None☐ Other☐ (SPECIFY)____________
	School's water source for cleaning: Tap☐ River☐ Rain☐ Borehole☐ Water Truck/Bowser☐ None☐ Other☐ (SPECIFY)____________

**Type of Fees Paid by Parents/Guardians for Class 7 Directly to School**	**FIRST TERM 2016 Amount (Per Term)**	**SECOND TERM 2016 Amount (Per Term)**	**THIRD TERM 2016 Amount (Per Term)**	**FIRST TERM 2017 Amount (Per Term)**
Tuition/Extra Classes				
Room and Board				
Maintenance/Construction				
Examinations				
Activity fee				
School Meals				
Transport				
Other 1 (Specify) ________________________				
TOTAL				

		YES	NO	DK
**A**	Did the school provide soap for handwashing for the students in the previous term?	1	2	8
**B**	Did the school provide sanitary pads for pupils in the previous school term?	1	2	8

	(i) If YES, how often? 1 = Monthly, 2 = Twice, 3 = Only once
	(ii) To whom were sanitary pads provided? 1 = All girls; 2 = Only the menstruating girls
	(iii) To which classes were sanitary pads provided?<br>Class 3 Yes ☐ No☐<br>Class 4 Yes ☐ No☐<br>Class 5 Yes ☐ No☐<br>Class 6 Yes ☐ No☐<br>Class 7 Yes ☐ No☐<br>Class 8 Yes ☐ No☐
	(iv) If the sanitary pads were provided on an individual basis, please specify________________________

Name of Head of the school: _ CellPhone Number _________________________

Signature and Stamp: _ _ _ _ _ __ _ __ _ _ _ _ _ _

OBSERVATION

Section A: Rooms

**A1**	In total, how many classrooms does the school have? .... **[____]**							
	**Instruction: visit each classroom on school site. For each room, record:**							
		**A2** Structure type	**A3** Roof type	**A4** Flooring type	**A5** Blackboard	**A6** Teacher's desk / table	**A7** Desks / chairs for students	
		0 = Poles / pillars<br>1 = Damaged / partial walls<br>2 = Complete walls	0 = No roof<br>1 = Damaged / partial roof<br>2 = Complete roof	0 = Dirt<br>1 = Cement	0 = No blackboard<br>1 = Blackboard	0 = Not present<br>1 = Present	0 = Not present<br>1 = present	
	**A**							
	**B**							
	**C**							
	**D**							
	**E**							
	**F**							
	**G**							
	**H**							
	**I**							
	**J**							
	**K**							
	**L**							
	**M**							
	**N**							
	**P**							
	**Q**							
	**R**							
	**S**							
	**T**							
	**U**							

			NO	YES
**A8**	Does the school have the following dedicated rooms or facilities?	Library	0	1
		Secure book/storage room	0	1
		Sports / other equipment storage	0	1
		Staff room	0	1
		Head teacher's office	0	1

Section B: Toilet Facilities

Indicate how many toilets the school has for use how many are for use exclusively by boys/exclusively girls/exclusively for staff/shared				
**Instruction: Record total number of toilets and whether they are functional or not**	Boys toilets	Girls toilets	Shared toilets	Staff toilets
Functional	[____]	[____]	[____]	[____]
Not Functional	[____]	[____]	[____]	[____]

	Select one functional toilet for each user type that represents the majority of toilets for that user, and indicate the door type, privacy, structure material and their functionality.				
		**Toilet 1**	**Toilet 2**	**Toilet 3**	**Toilet 4**
**A**	Users:				
	Boys	1	1	1	1
	Girls	2	2	2	2
	Shared	3	3	3	3
	Staff only	4	4	4	4
	Other (Specify)	___________	___________	___________	_________
**B**	Door type:				
	Floor to ceiling door	1	1	1	1
	Partial door	2	2	2	2
	Fabric/gunia/nylon	3	3	3	3
	None	4	4	4	4
	Other (Specify)	____________	___________	__________	________
**C**	Privacy (Latch/lock):				
	Yes	1	1	1	1
	No	2	2	2	2
**D**	Structure material:				
	Corrugated Iron	1	1	1	1
	Wood	2	2	2	2
	Cement	3	3	3	3
	Mud	4	4	4	4
	Other (Specify)	_____________	________	__________	__________

Section C: Handwashing

Indicate how many hand washing stations the school has for use by pupils and how many are for use exclusively by boys/exclusively girls/shared					
**Instruction: Record total number of handwashing stations and whether for use by boys/girls/shared**	Total Number not functional	Total Number functional	Number functional for boys only	Number functional for girls only	Number functional for boys & girls/ Shared
Handwashing stations	[____]	[____]	[____]	[____]	[___]

Section D: Sanitation

Indicate whether the school provides the following:			
		**YES**	**NO**
**A**	Does the school have any sanitary pads in stock?	1	2
**B**	Is the storage area locked?	1	2
**C**	Did the school provide soap for handwashing for the students today?	1	2

Section E: Record Keeping & Registers

	**Instruction: Ask to observe the school's registers for class 4 - 8. For each class, record:**							
			**a.** Class 4	**b.** Class 5	**c.** Class 6	**d.** Class 7	**e.** Class 8	
**C1**	Is there an 'official' register or a makeshift one (e.g. exercise book)?	Official register<br>Makeshift register<br>No register	2<br>1<br>0	2<br>1<br>0	2<br>1<br>0	2<br>1<br>0	2<br>1<br>0	
**C2**	Look at the past 5 school-days. Has the register been:	Filled in completely<br>Filled in partially<br>Not filled in<br>Reg. not observed	2<br>1<br>0<br>99	2<br>1<br>0<br>99	2<br>1<br>0<br>99	2<br>1<br>0<br>99	2<br>1<br>0<br>99	
