# Supplementary material for: Cluster randomized evaluation of the Nia Project: study protocol
Source: Reprod Health. 2018 Dec 29;15:218. doi: 10.1186/s12978-018-0586-4 (PMC6310925; doi:10.1186/s12978-018-0586-4)
Supplement: Supplementary file 2 — Nia Baseline Tool (PDF 1134 kb) [file 12978_2018_586_MOESM2_ESM.pdf]

THE NIA PROJECT

Baseline Survey Instruments

POPULATION COUNCIL
Ideas. Evidence. Impact.

SERIAL #: [_______________________]

ENGLISH

SECTION 1: RESPONDENT'S BACKGROUND

	QUESTIONS	RESPONSES	SKIP TO	
101	**[RECORD THE TIME INTERVIEW BEGINS USE 24 HOUR TIME]**	**a.** HOUR [6-20] ☐☐<br>**b.** MINUTES [0-59] ☐☐		101
102	In what month and year were you born?	**a.** MONTH [1-12] ☐☐<br>DON'T KNOW MONTH 88<br>**b.** YEAR [1996-2007] ☐☐☐☐<br>DON'T KNOW YEAR 8888		102
103	How old were you on your last birthday?<br>**[COMPARE AND CORRECT 102 AND/OR 103 IF INCONSISTENT]**	AGE IN COMPLETED YEARS [10-20] ☐☐		103
104	What class in school are you attending now?	STANDARD [1-8] ☐<br>**IF NOT CLASS 7**	**END INT**	104
105	In which **county** were you born?<br>Note: Update with additional Countries from Pre-Test	COUNTY CODE [01-49] ☐☐<br>BORN IN SOMALIA 66<br>BORN IN ETHIOPIA 77<br>DON'T KNOW 88<br>BORN IN OTHER COUNTRY ______________ 98<br>SPECIFY		105
106	What is the name of the place where you are currently living?<br>Note: Pre-populated list	______________________________		106
107	How long have you been living continuously in [name of current place of residence]?<br>**[IF LESS THAN ONE YEAR, RECORD '0' YEARS]**	YEARS [0- 20] ☐☐<br>ALWAYS (SINCE BIRTH) 77<br>DON'T KNOW 88	**109**	107
108	Think about the place where you lived just before you moved here. Was it a city, a town, or a village?	TOWN / URBAN AREA 1<br>VILLAGE / RURAL AREA 2		108
109	In the last one year, on how many separate occasions have you traveled away from your home community and slept away?	NUMBER OF TRIPS [1-80] ☐☐<br>NONE 0<br>DON'T KNOW 88	**110**	109
	**a.** In the previous school term (between September and October last year), how many days in total were you away from your home community?	NUMBER OF DAYS [1-70] ☐☐<br>NONE	**110**	
	**b.** What were the reasons you were away for the days you just mentioned?<br>**[READ ALL OPTIONS]**	see below		
110		YES 1	**112**	110

	Reason	YES	NO
**a.**	For marriage	1	2
**b.**	For school	1	2
**c.**	For medical reasons	1	2
**d.**	Could not afford to live where I was	1	2
**e.**	Accompanyiing family members	1	2
**f.**	Personal problems at home (family dispute, abuse)	1	2
**g.**	Go for work	1	2
**h.**	Escaping early marriage	1	2
**i.**	Separation or divorce from husband or partner	1	2
**j.**	Death of parent(s)/guardian	1	2
**k.**	Separation or divorce of parents	1	2
**l.**	Death of husband/partner	1	2
**m..**	Political violence	1	2
**n.**	Famine or poverty in the other place	1	2
**o.**	Stigma due to HIV status	1	2
**p.**	Lack of school fees	1	2
**q.**	Other forms of stigma/discrimination	1	2
**r**	Other (SPECIFY)		98

	Is your mother alive?	NO 2<br>DON'T KNOW 88	 **112**		
**111**	How old were you when your mother died?	AGE IN YEARS [ ]<br>DON'T KNOW 88			**111**
**112**	What is the highest level of education your mother completed?	NO EDUCATION 1<br>NURSERY SCHOOL 2<br>SOME PRIMARY SCHOOL 3<br>COMPLETED PRIMARY SCHOOL 4<br>SOME SECONDARY SCHOOL 5<br>COMPLETED SECONDARY SCHOOL 6<br>PRE-SECONDARY SCHOOL VOCATIONAL/TECHNICAL TRAINING 7<br>POST- SECONDARY SCHOOL VOCATIONAL/TECHNICAL TRAINING 8<br>COLLEGE/UNIVERSITY 9<br>DON'T KNOW 88			**112**
**113**	Is your father alive?	YES 1<br>NO 2<br>DON'T KNOW 88	**115**<br><br>**115**		**113**
**114**	How old were you when your father died?	AGE IN YEARS [ ]<br>DON'T KNOW 88			**114**
**115**	What is the highest level of education your father completed?	NO EDUCATION 1<br>NURSERY SCHOOL 2<br>SOME PRIMARY SCHOOL 3<br>COMPLETED PRIMARY SCHOOL 4<br>SOME SECONDARY SCHOOL 5<br>COMPLETED SECONDARY SCHOOL 6<br>PRE-SECONDARY SCHOOL VOCATIONAL/TECHNICAL TRAINING 7<br>POST- SECONDARY SCHOOL VOCATIONAL/TECHNICAL TRAINING 8<br>COLLEGE/UNIVERSITY 9<br>DON'T KNOW 88			**115**
**116**	What is your ethnic group?<br><br>**[RECORD ALL MENTIONED]**	YES NO<br>EMBU 1 2<br>KALENJIN 1 2<br>KAMBA 1 2<br>KIKUYU 1 2<br>KISII 1 2<br>LUHYA 1 2<br>LUO 1 2<br>MASAI 1 2<br>MERU 1 2<br>MIJIKENDA/SWAHILI 1 2<br>SOMALI 1 2<br>BORANA 1 2<br>TAITA/TAVETA 1 2<br>NUBIAN 1 2<br>OTHER ______________ 1 2<br>SPECIFY			**116**
**117**	What language(s) do you speak at home?<br><br>**[RECORD ALL MENTIONED]**<br><br>Note: Update with additional Mijikenda languages	YES NO<br>**a.** EMBU 1 2<br>**b.** KALENJIN 1 2<br>**c.** KAMBA 1 2<br>**d.** KIKUYU 1 2<br>**e.** KISII 1 2<br>**f.** LUHYA 1 2<br>**g.** LUO 1 2<br>**h.** MASAI 1 2<br>**I.** MERU 1 2<br>**j.** GIRIAMA 1 2<br>**k.** SOMALI 1 2<br>**l.** KIBORANA 1 2<br>**m.** KISWAHILI 1 2<br>**n.** ENGLISH 1 2<br>**o.** NUBIAN 1 2<br>**p.** CHONYI 1 2<br>**q** RABAI 1 2<br>**r** DURUMA 1 2<br>**s** KAUMA 1 2<br>**t** DIGO 1 2<br>**u** KAMBE 1 2<br>**v** JIBANA 1 2<br>**w** RIBE 1 2<br>**x** OTHER (SPECIFY) ___________________ 1 2			**117**
**118**	Can you speak any other languages well enough to have a conversation? **[IF YES, ASK:]** Which ones?	YES NO<br>**a.** EMBU 1 2<br>**b.** KALENJIN 1 2			**118**

	[RECORD ALL MENTIONED]<br><br>Add Mijikenda option	**c.** KAMBA 1 2<br>**d.** KIKUYU 1 2<br>**e.** KISII 1 2<br>**f.** LUHYA 1 2<br>**g.** LUO 1 2<br>**h.** MASAI 1 2<br>**I.** MERU 1 2<br>**j.** GIRIAMA 1 2<br>**k.** SOMALI 1 2<br>**l.** KIBORANA 1 2<br>**m.** KISWAHILI 1 2<br>**n.** ENGLISH 1 2<br>**o.** NUBIAN 1 2<br>**P.** CHONYI 1 2<br>**q** RABAI 1 2<br>**r.** DURUMA 1 2<br>**s.** KAUMA 1 2<br>**t** DIGO 1 2<br>**u** KAMBE 1 2<br>**v** JIBANA 1 2<br>**w** RIBE 1 2<br>**p.** OTHER SPECIFY 1 2		
**119**	What is your religion?	CATHOLIC 1<br>PROTESTANT 2<br>ISLAM 3<br>TRADITIONAL 4<br>NO RELIGION 5<br>OTHER (SPECIFY_____) 98		**119**
**120**	How important is religion to you?	**READ OUT OPTIONS**<br>VERY IMPORTANT 1<br>SOMEWHAT IMPORTANT 2<br>NOT IMPORTANT 3		**120**
**121**	Now I want to ask you a few questions about the other people who live in your house. For each person that lives with you I will ask about their schooling and employment.Other than you, how many people live in your household?<br><br>**EXCLUDING RESPONDENT**	NUMBER OF PEOPLE [ ]<br><br>NONE 0	**123**	**121**
	**START BY ASKING WHO THEY LIVE WITH, CONTINUE TO PROBE ABOUT OTHERS WHO LIVE IN THE HOUSEHOLD UNTIL ALL WHO LIVE WITH THE RESPONDENT HAVE BEEN IDENTIFIED. HOUSEHOLD MEMBER IS DEFINED AS SOMEONE WHO REPORTS TO THE SAME HOUSEHOLD HEAD AND EATS FROM THE SAME POT. ASK SCHOOL/WORK QUESTIONS – 122e & 122f and 122g– ONLY OF PERSONS AGE 5 AND ABOVE**			

122

122a<br>Name	122b<br>Age / 8888=DK	122c<br>Sex	122d<br>Relationship to Respondent	122e<br>Highest Level Education Reached	122f<br>Are they in school? **[If < age 21]**	122g<br>Have they worked for pay in the last month? **[If parent/guardian]**
		F M			YES NO	YES NO DON'T KNOW
		1 2			1 2	1 2 88
		1 2			1 2	1 2 88
		1 2			1 2	1 2 88
		1 2			1 2	1 2 88
		1 2			1 2	1 2 88
		1 2			1 2	1 2 88
		1 2			1 2	1 2 88

CODES 122e

1 NO EDUCATION
2 NURSERY SCHOOL
3 PRIMARY SCHOOL
4 SECONDARY SCHOOL
5 VOCATIONAL/TECHNICAL TRAINING
6 COLLEGE/ UNIVERSITY
7 NON-FORMAL SCHOOL
88 DON'T KNOW

CODES 122d:

0	Mother	8	Uncle
1	Father	9	Friend
2	Brother	10	Boyfriend/Husband
3	Sister	11	Own Child
4	Grandmother	12	Employer
5	Grandfather	13	Guardian
		14	Niece/nephew
		15	Other relative
6	Cousin	96	Other(SPECIFY)
7	Aunt		

**123**	What is the main material used for the EXTERIOR WALLS of the house you live in?	MUD 1<br>CEMENT 2<br>CORRUGATED IRON 3<br>WOOD 4<br>STONES 5<br>BRICKS 6<br>GRASS/MAKUTI 7<br>OTHER(SPECIFY) _____ 96		**123**
**124**	What is the main material used for the ROOF of the house you live in?	CORRUGATED IRON 1<br>PLASTIC 2		**124**

		PLYWOOD 3<br>TILES 4<br>MAKUTI/GRASS 5<br>OTHER(SPECIFY) _______ 96		
125	What is the main material used for the FLOOR of the house you live in?	MUD 1<br>CEMENT 2<br>WOOD/CEMENT WITH ADDITIONAL COVERING 3<br>OTHER(SPECIFY)_______ 96		125
126	What is the main source of drinking water for members of your household?	PIPED WATER PIPED INTO RESIDENCE/COMPOUND/PLOT 1<br>PUBLIC TAP WHERE WATER IS FREE 2<br>PUBLIC TAP WHERE WATER IS BOUGHT 3<br>WELL WATER IS BOUGHT 4<br>WELL ON RESIDENCE/PLOT 5<br>PUBLIC WELL 6<br>SURFACE WATER/RIVER/STREAM/POND/LAKE 7<br>RAINWATER 8<br>WATER VENDOR<br>OTHER (SPECIFY) 98	130 (1)<br>130 (WATER VENDOR)	126
127	Where is the water source located?	IN OWN DWELLING 1<br>IN OWN YARD/PLOT 2<br>ELSEWHERE 3	130 (1)<br>130 (2)	127
128	How long does it take to walk there, get water and come back?	a. HOURS [0-8] [ ][ ]<br>b. MINUTES [0-480] [ ][ ]<br>DON'T KNOW 88		128
129	Who usually goes to this source to fetch water for your house hold?	SELF 1<br>FEMALE AGE 15 AND ABOVE 2<br>MALE AGE 15 AND ABOVE 3<br>OTHER FEMALE CHILD UNDER 15 YEARS OLD 4<br>MALE CHILD UNDER 15 YEARS OLD 5<br>OTHER ____________ 98<br>SPECIFY		129
130	What kind of toilet facility do members of your house hold use?	FLUSH TOILET 1<br>VENTILATED PIT LATRINE 2<br>UNVENTILATED PIT LATRINE 3<br>NO FACILITY/BUSH/FIELD 4<br>POUR FLUSH 5<br>OTHER ____________ 98<br>SPECIFY	133 (4)	130
131	Do you share this toilet facility with other households?	YES 1<br>NO 2	133 (2)	131
132	How many households use this toilet facility?	NO. OF HOUSEHOLDS<br>IF LESS THAN 10 [ ]<br>10 OR MORE HOUSEHOLDS 95<br>DON'T KNOW 88		132
133	How many rooms in your household are used for sleeping?	NUMBER OF ROOMS [ ]		133
134	How many people share the same sleeping room with you?	NUMBER OF MALES [ ][ ]<br>NUMBER OF FEMALES [ ][ ]		134
135	In the last one month, was there a day that you went without food because there wasn't enough food in the household?	YES 1<br>NO 2		135

136 READ RESPONSE OPTIONS
Now I want to ask you about different items that you may or may not have in your household. Does anyone in the household that you live in own or have these items?

Item	NO	YES
A. ELECTRICITY	1	2
B. RADIO	1	2
C. TELEVISION	1	2
D. TELEPHONE/MOBILE PHONE	1	2
E. KEROSENE LAMP	1	2
F. BED	1	2
G. BICYCLE	1	2
H. MOTORCYCLE	1	2
I. CAR	1	2
J. THE HOUSE YOU LIVE IN	1	2
K. LAND	1	2
L. SMALL LIVESTOCK (i.e. Poultry, goats	1	2
M. LARGE LIVESTOCK (i.e. cows, pigs	1	2
N. REFRIGERATOR	1	2
O. TOOLS/MACHINE PEOPLE USE TO EARN MONEY	1	2

136

	P. JEWELRY	1 2		
	**SECTION 2: USE OF SKILLS**			
**201**	In your everyday life, how often do you usually read any written materials such as newspapers, pamphlets, magazines, books, or the Bible/Koran ?<br><br>**[READ RESPONSE OPTIONS]**	ALMOST EVERY DAY 1<br>AT LEAST ONCE A WEEK 2<br>AT LEAST ONCE A MONTH 3<br>LESS THAN ONCE A MONTH 4<br>NEVER 5		**201**
**202**	In your everyday life, how often do you usually write anything ?<br><br>**[READ RESPONSE OPTIONS]**	ALMOST EVERY DAY 1<br>AT LEAST ONCE A WEEK 2<br>AT LEAST ONCE A MONTH 3<br>LESS THAN ONCE A MONTH 4<br>NEVER 5		**202**
**203**	In your everyday life, how often do you usually listen to the radio?<br><br>**[READ RESPONSE OPTIONS]**	ALMOST EVERY DAY 1<br>AT LEAST ONCE A WEEK 2<br>AT LEAST ONCE A MONTH 3<br>LESS THAN ONCE A MONTH 4<br>NOT AT ALL 5		**203**
**204**	In your everyday life, how often do you usually watch television?<br><br>**[READ RESPONSE OPTIONS]**	ALMOST EVERY DAY 1<br>AT LEAST ONCE A WEEK 2<br>AT LEAST ONCE A MONTH 3<br>LESS THAN ONCE A MONTH 4<br>NOT AT ALL 5		**204**
	**SECTION 3: RESPONDENT'S SCHOOLING**			
	**QUESTIONS**	**RESPONSES**		
**301**	At what age did you start Standard 1?	AGE [ ][ ]<br>[4-14]<br>DON'T KNOW 88		**301**
**302**	Have you ever repeated a class in school?	YES 1<br>NO 2	 **304**	**302**
**303**	Please tell me which class you repeated and how many times?<br><br>**RECORD ALL MENTIONED.**	Number of imes Repeated<br>CLASS 1 [ ]<br>CLASS 2 [ ]<br>CLASS 3 [ ]<br>CLASS 4 [ ]<br>CLASS 5 [ ]<br>CLASS 6 [ ]<br>CLASS 7 [ ]<br>[1-5]<br>NONE 96		**303**
**304**	What is the name of the school you attend?<br><br>Note: Pre-populated list	______________________________		**304**
**305**	Do you live at home or somewhere else to attend school?	LIVE AT HOME 1<br>LIVE SOMEWHERE ELSE 2		**305**
**306**	How do you usually get to school?	BY FOOT / WALKING 1<br>OWN BICYCLE/HOUSEHOLD BICYCLE 2<br>HIRED BICYCLE TAXI/BODA BODA 3<br>BUS/MATATU/MINIBUS/OTHER PUBLIC TRANSPORT 4<br>PRIVATE VEHICLE 5<br>OTHER (SPECIFY_____) 98	**308**<br>**308**<br><br><br>**308**<br>**308**	**306**
**307**	How much fare do you usually pay in total to get to school and back?	a. PER DAY 1<br>PER MONTH 2<br>PER TERM 3<br><br>b. KENYA SHILLINGS [ ][ ][ ][ ][ ]		**307**

		DON'T KNOW 88888		
308	How long does it usually take you to get to school?	a. HOURS [0-5]<br>b. MINUTES [0-300]<br>DON'T KNOW 88		308
309	In the previous school term (between September and October last year), did you miss school even for one day?	YES 1<br>NO 2	 313	309
310	What were the reasons you missed school during the previous school term? How many days did you miss school for that reason? Any other reason?<br>**DO NOT READ LIST. RECORD ALL MENTIONED.**	Days Missed<br>SELF SICK/ILL<br>NEEDED TO WATCH BROTHERS/SISTERS<br>HOUSEHOLD MEMBER SICK<br>MARKET DAY<br>NEEDED TO DO CHORES AT HOME<br>NEEDED TO RUN ERRANDS FOR PARENTS<br>UNIFORM DIRTY<br>PROBLEMS WITH/AFRAID OF OTHER STUDENTS<br>PROBLEMS WITH/AFRAID OF TEACHER<br>MENSTRUATION<br>WOULD RATHER DO SOMETHING ELSE<br>WENT TO SEE/WAS WITH BOYFRIEND<br>BEREAVEMENT/FUNERAL<br>SCHOOL FEES NOT PAID<br>OTHER (SPECIFY_____)<br>[1-80] 0<br>NONE 96	**313**	310
311	What was the **MAIN** reason you missed school during the previous term?	SELF WAS SICK/ILL 1<br>NEEDED TO WATCH BROTHERS/SISTERS 2<br>HOUSEHOLD MEMBER SICK 3<br>MARKET DAY 4<br>NEEDED TO DO CHORES AT HOME 5<br>WAS SENT SOMEWHERE BY PARENTS/GUARDIAN 6<br>UNIFORM DIRTY 7<br>PROBLEMS WITH/AFRAID OF OTHER STUDENTS 8<br>PROBLEMS WITH/AFRAID OF TEACHER 9<br>MENSTRUATION 10<br>WOULD RATHER DO SOMETHING ELSE 11<br>WENT TO SEE/WAS WITH BOYFRIEND 12<br>BEREAVEMENT/FUNERAL 13<br>SCHOOL FEES NOT PAID 14<br>OTHER (SPECIFY_____) 98		311
**FILTER**	**IF MISSED DUE TO MENSTRUATION IN 310 OR 311, ASK 312. OTHERS SKIP TO 313.**			**FILTER**
312	When you missed class due to menstruation, what was/were the reason(s) you missed class?<br>**[CHECK ALL THAT APPLY]**	YES NO<br>a. You had nothing (e.g.sanitary pads or cloth) to manage period 1 2<br>b. You were scared to have accident at school 1 2<br>c. You had pain/cramps/headache 1 2<br>d. You were embarrased 1 2<br>e. You were scared to be teased at school 1 2<br>f. There is no private place to manage period at school 1 2<br>g. Lack of water/place to clean 1 2<br>h. Diarrhea 1 2<br>i. Other (SPECIFY_____________) 98		312
	If you said other, please specify	____		
313	In the past school **week**, how many days was your school in session?	DAYS<br>[0-7]<br>**IF 0**	**316**	313
314	In the past school week, how many days did you attend school?	DAYS<br>[0-7]<br>**IF 313 = 314]**	**316**	314
315	In the past week, why did you miss school?<br>**[CHECK ALL THAT APPLY]**	**YES NO**<br>SELF WAS SICK/ILL 1 2<br>NEEDED TO WATCH BROTHERS/SISTERS 1 2<br>HOUSEHOLD MEMBER SICK 1 2<br>MARKET DAY 1 2<br>NEEDED TO DO CHORES AT HOME 1 2		315

		WAS SENT SOMEWHERE BY PARENTS/GUARDIAN 1 2<br>UNIFORM DIRTY 1 2<br>PROBLEMS WITH/AFRAID OF OTHER STUDENTS 1 2<br>PROBLEMS WITH/AFRAID OF TEACHER 1 2<br>MENSTRUATION 1 2<br>WOULD RATHER DO SOMETHING ELSE 1 2<br>WENT TO SEE/WAS WITH BOYFRIEND 1 2<br>BEREAVEMENT/FUNERAL 1 2<br>OTHER (SPECIFY_____) 1 2		
	If you said other, please specify	_________________________________		

316 **School Engagement and Participation**

Now we would like to ask you about your participation and interest in school. For these questions we would like you to tell us if you disagree or agree with the following statements.

**[Read options 1 and 2 only]**	**AGREE**	**DISAGREE**	**NO OPINION /DON'T KNOW**
**a.** You do not attend school regularly. Do you... (E-)	1	2	88
**b**. You are attentive in class. Do you... (E+)	1	2	88
**c**. You rarely complete the tasks that are assigned to you in class. Do you... (E-)	1	2	88
**d**. Education is not important to you. Do you ..(A-)	1	2	88
**e**. You respect your teachers. Do you… (A+)	1	2	88
**f**. There is no adult at school whom you can talk to if you have a problem. Do you… (A-)	1	2	88
**g.** You study at home even if you do not have an exam. Do you… (E+)	1	2	88
**h.** Your teacher would not notice if you were absent from school. Do you…. (A-)	1	2	88
**i**. You often feel that school is boring	1	2	88
**Schooling Competence**			
**j.** You have the talent to learn your lessons well. Do you... (+)	1	2	88
**k**. You often do not understand the lessons in class. Do you...	1	2	88
**l**. It is difficult for you to complete your schoolwork. Do you... (-)	1	2	88
**m.** You feel comfortable participating in class	1	2	88
**n. You do not feel confident answering questions in class**	1	2	88
**o**. It It is often difficult for you to pay attention in class	1	2	88
**p**. You always complete your school work	1	2	88
**q.** You believe you are capable of doing well in school	1	2	88
**Self Esteem**			
**r**. You are as good or better at doing many things as others of your age. Do you... (-)	1	2	88
**s**. Your opinion is valued in your household. Do you... (+)	1	2	88
**t**. People think that you have a lot of good qualities. Do you…(+)	1	2	88
**u**. You cannot handle or succeed in many things that you do. Do you... (-)	1	2	88

316

**317**	What is success in school most determined by? Talent, luck or hard work? (-)	TALENT 1<br>LUCK 2<br>HARDWORK 3<br>COMBINATION 4<br>NO OPINION/DON'T KNOW 88		**317**
	**Education and Schooling Experience**<br>I am going to read a series of statements about education and schooling. I want you to tell me if you agree or disagree with the statement.			
**318**	a. It is as important for girls to complete secondary school as it is for boys.	AGREE 1<br>DISAGREE 2		**318**
	b. When a family cannot afford to send all children to school, it is better to send boys than girls.	AGREE 1<br>DISAGREE 2		

	c. A 16-year old girl should get married when she finds an appropriate partner, even if she is still in school.	AGREE 1<br>DISAGREE 2		
319	Now I am going to read a series of statements about your current schooling experience. I want you to tell me if the statement applies to you or your experience.<br><br>YES / NO / NA<br>a. Most of your teachers favor boys in class over girls 1 2 8<br>b. There is a female teacher who encourages you to do well in school 1 2 8<br>c. Your father approves of you going to school 1 2 8<br>d. Your mother approves of you going to school 1 2 8<br>e. At times, you have so much work at home, you cannot complete homework 1 2 8			319
320	Who receives more positive comments from your class teacher: boys, girls, or do they receive about the same amount?	NO POSITIVE COMMENTS MADE………… 0<br>BOYS…………………………………………… 1<br>GIRLS………………………………………… 2<br>ABOUT THE SAME…………………………… 3<br>DON'T KNOW...................................... 88		320
321	Who receives more negative comments from your class teacher: boys, girls or do they receive about the same amount?	NO NEGATIVE COMMENTS MADE………… 0<br>BOYS…………………………………………… 1<br>GIRLS…………………………………………… 2<br>ABOUT THE SAME…………………………….. 3<br>DON'T KNOW....................................... 88		321
322	Did you ever learn life skills education/family life education in your school?	Yes 1<br>No 2	 327	322
323	In the last year (2016), did any of your teachers cover any of the following topics in class?<br><br>**[READ LIST; CHECK ALL THAT APPLY]**	YES / NO<br>COMMUNICATION 1 2<br>SELF-ESTEEM 1 2<br>PREGNANCY 1 2<br>PUBERTY/HOW BOYS AND GIRLS MATURE 1 2<br>HIV/AIDS 1 2<br>MARRIED LIFE/ROLES IN FAMILY 1 2<br>MENSTRUATION 1 2<br>CONTRACEPTIVES/FAMILY PLANNING 1 2<br>ABSTINENCE/AVOIDING SEX 1 2<br>HEALTH AND HYGIENE 1 2<br>OTHER (SPECIFY___) 98	**If all 'NO':**<br>**325**	323
324	Who taught those sessions?<br><br>**[READ LIST, CHECK ALL THAT APPLY]**	Male teacher 1 2<br>Female teacher 1 2<br>Guidance counselor 1 2<br>OTHER (SPECIFY___) 98		324
325	In the last year (2016), did someone other than your teachers (someone from outside or from an NGO or CBO) teach you any of the following topics in school?<br><br>**[READ LIST; CHECK ALL THAT APPLY]**	YES / NO<br>COMMUNICATION 1 2<br>SELF-ESTEEM 1 2<br>PREGNANCY 1 2<br>PUBERTY/HOW BOYS AND GIRLS MATURE 1 2<br>HIV/AIDS 1 2<br>MARRIED LIFE/ROLES IN FAMILY 1 2<br>MENSTRUATION 1 2<br>CONTRACEPTIVES/FAMILY PLANNING 1 2<br>ABSTINENCE/AVOIDING SEX 1 2<br>HEALTH AND HYGIENE 1 2<br>OTHER (SPECIFY) 98	**If all 'NO':**<br>**327**	325
326	How often did they teach you these topics?	EVERY WEEK 1<br>AT LEAST TWICE PER MONTH 2<br>ONCE OR TWICE PER TERM 3<br>LESS OFTEN 4		326
327	What is the highest level of school you would like to complete?	**DO NOT READ LIST**<br>PRIMARY SCHOOL 1<br>SECONDARY SCHOOL 2<br>PRE - SECONDARY VOCATIONAL/TECHNICAL TRAINING 3<br>POST-SECONDARY VOCATIONAL/TECHNICAL TRAINING 4<br>COLLEGE/UNIVERSITY 5		327
328	Sometimes people are not able to go as far in school as they would like for many reasons. How much school do you actually <u>expect to</u> complete?	**DO NOT READ LIST**<br>PRIMARY SCHOOL 1<br>SECONDARY SCHOOL 2<br>PRE - SECONDARY VOCATIONAL/TECHNICAL TRAINING 3<br>POST-SECONDARY VOCATIONAL/TECHNICAL TRAINING 4<br>COLLEGE/UNIVERSITY 5	 <br>401<br><br>401<br>401	328
329	What is the main reason why you think you will complete secondary school?	**DO NOT READ LIST**<br>EXPECT TO GET MARRIED 1<br>CURRENTLY PREGNANT 2		329

		CAN'T PAY SCHOOL FEES 3<br>CAN'T AFFORD UNIFORM 4<br>PARENTS NOT SUPPORTIVE 5<br>DOMESTIC RESPONSIBILITIES 6<br>DON'T EXPECT TO PASS KCPE EXAM 7<br>DON'T EXPECT TO GET A PLACE IN SECONDARY 8<br>OTHER (SPECIFY_______) 98	

SECTION 4: WORK, SAVINGS, AND FINANCIAL LITERACY

	QUESTIONS	RESPONSES		
401	Yesterday, how much time did you spend doing household chores, such as cooking, cleaning, laundry, collecting firewood, water?<br>**[IF YESTERDAY WAS FRIDAY, SATURDAY OR SUNDAY, ASK ABOUT THURSDAY]**	**a.** HOURS [0-12] [ ][ ]<br>**b.** MINUTES [0-720] [ ][ ]<br>DON'T KNOW 88		401
402	Aside from your housework, have you done any work for which you were paid in cash or kind (given something in return) in the last	YES 1<br>NO 2	**405**	402
403	As you know, some girls take up jobs for which they are paid in cash or kind. Others sell things, have a small business or work on the family farm or in the family business. Just to be sure, in the last month, have you done any chores or activities for which you got paid or were given something in return (for example food, or other goods)?	YES 1<br>NO 2	**405**	403
404	Aside from your housework, have you done any work or chores or activities for which you were paid in cash or kind in the last one year?	YES 1<br>NO 2	**407**	404
405	How old were you the very first time you did any work or activity for pay, whether you were paid in cash or kind?	AGE [4-20] [ ][ ]<br>DON'T KNOW AGE 88		405
406	Which of the following kinds of activities or work did you do for which you were paid in cash or in kind?<br>**[READ ALL OPTIONS]**	YES NO<br>**a.** Domestic work (Maid) 1 2<br>**b.** Wash clothes/utensils/house 1 2<br>**c.** Fetching water 1 2<br>**d.** Plaiting hair/beauty 1 2<br>**e.** Babysit/child care 1 2<br>**f.** Work in a restaurant 1 2<br>**g.** Work in a farm 1 2<br>**h.** Packaging food or drinks 1 2<br>**i.** Selling things 1 2<br>**j.** Work in an office 1 2<br>**k.** Other type of own business 1 2<br>**k.** Other type of temporary job 1 2<br>**l.** Other type of employment 1 2		406
407	I want to talk about the items that a person might own. Do you personally own or have these items?<br>**[READ LIST; IF 'YES' ASK 408]**	**408**<br>Who last gave you [ITEM], gave you money to buy this item, or purchased this item for you?<br>USE THE FOLLOWING CODES:<br>[DO NOT READ LIST]<br>SELF- WITH MONEY EARNED 1<br>MOTHER 2<br>FATHER 3<br>GUARDIAN 4<br>BOYFRIEND 5<br>HUSBAND 6<br>SUGAR DADDY 7<br>MALE FRIEND 8<br>FEMALE FRIEND 9<br>OTHER RELATIVE 10<br>TEACHER 11<br>OTHER (SPECIFY_____________) 98		407

		YES	NO			CODE
**a.**	A PAIR OF SHOES	1	2	**a.**	A PAIR OF SHOES	[ ][ ]
**b.**	SCHOOL UNIFORM	1	2	**b.**	SCHOOL UNIFORM	[ ][ ]
**c.**	TWO SETS OF CLOTHES (OTHER THAN UNIFORM)	1	2	**c.**	TWO SETS OF CLOTHES (OTHER THAN UNIFORM)	[ ][ ]
**d.**	TWO UNDERWEARS	1	2	**d.**	TWO UNDERWEARS	[ ][ ]
**e.**	A SCHOOL BAG	1	2	**e.**	A SCHOOL BAG	[ ][ ]

	**f.** SOME JEWELRY 1 2	**f.** SOME JEWELRY [ ][ ]		
	**g.** A MOBILE TELEPHONE 1 2	**g.** A MOBILE TELEPHONE [ ][ ]		
	Now I am going to ask you several questions about different issues related to your use of any money you earned or money you were given, spending money, and savings. We know that some girls have money to spend and save and others don't. So, there are no **right** or **wrong** answers, just answer as honestly as you can.			
**409**	In the last one year, did you spend any money on your daily needs, other odds and ends, or other expenses?	YES 1<br>NO 2	 **412**	**409**
**410**	In the last one year, when you spent money on your daily needs, other odds and ends, or other expenses, where did you get that money from?<br><br>**[RECORD ALL MENTIONED]**	YES NO<br>**a.** MOTHER 1 2<br>**b.** FATHER 1 2<br>**c.** GUARDIAN YOU LIVE WITH 1 2<br>**d.** OTHER RELATIVES 1 2<br>**e.** FRIEND 1 2<br>**f.** BOYFRIEND 1 2<br>**g.** HUSBAND 1 2<br>**h.** SUGAR DADDY 1 2<br>**i.** STRETCHING MONEY 1 2<br>**j.** OWN SAVINGS 1 2<br>**k.** SAVINGS GROUP/CHAMA 1 2<br>**l.** CASUAL JOBS/CHORES 1 2<br>**m.** STEADY JOB 1 2<br>**n.** CASH TRANSFERS 1 2<br>**o.** SPONSOR 1 2<br>**p.** OTHER (SPECIFY________) 1 2		**410**
**411**	In the past month, did you spend money on this item?<br>(READ EACH ITEM ALOUD. ASK YES OR NO)<br><br>**(IF NO ON A, GO TO NEXT ITEM, SKIP B, C)**<br>**NOTE: Clarify if it is a personal spending need or for someone else if it is not clear from the type of expenditure)**			**411**

	**A.** In the past month, did you spend money on this item?	**B.** About how much did you spend in one MONTH on this item?	**C.** Do you decide to buy this item on your own, together with someone, or someone else decides for you?
		Ksh	**1 = YOU ALONE**<br>**2 = YOU TOGETHER WITH SOMEONE**<br>**3 = SOMEONE ELSE**
**a** FOOD, CHIPS, SWEETS, DRINKS/SODA FOR YOURSELF	YES 1<br>NO 2		
**b** FOOD, CHIPS, SWEETS, DRINKS/SODA FOR SOMEONE ELSE	YES 1<br>NO 2		
**c** CLOTHES/SHOES FOR YOURSELF	YES 1<br>NO 2		
**d** CLOTHES/SHOES FOR SOMEONE ELSE	YES 1<br>NO 2		
**e** SANITARY PADS	YES 1<br>NO 2		
**f** BEAUTY PRODUCTS/ SERVICES	YES 1<br>NO 2		
**g** CREDIT/AIRTIME	YES 1<br>NO 2		

	h ENTERTAINMENT	YES 1<br>NO 2			
	i TRANSPORT	YES 1<br>NO 2			
	j RENT	YES 1<br>NO 2			

412	In the past six months, have you saved, or put money aside to use at a later time?	YES 1<br>NO 2	 417	412
413	Would you say that you saved on a WEEKLY BASIS always, usually, sometimes, or never?	ALWAYS 1<br>USUALLY 2<br>SOMETIMES 3<br>NEVER 4	 <br><br>417	413
414	In total, how many shillings do you currently have saved?	KENYA SHILLINGS [ ][ ][ ][ ][ ]<br>DON'T KNOW 8888		414
415	What are you saving for?<br>**[DO NOT READ LIST]**	**CHECK ALL THAT APPLIES**<br>YES NO<br>**a.** EMERGENCIES 1 2<br>**b.** PERSONAL ITEMS 1 2<br>**c.** HOUSEHOLD EXPENSES 1 2<br>**d.** OWN SCHOOL FEES 1 2<br>**e.** SCHOOL FEES FOR OTHERS 1 2<br>**f.** SCHOOL SUPPLIES 1 2<br>**g.** OWN BUSINESS 1 2<br>**h.** FAMILY BUSINESS 1 2<br>**i.** GENERAL FUTURE USE 1 2<br>**j.** AGRICULTURAL INPUTS 1 2<br>**k.** MEDICAL EXPENSES FOR SELF 1 2<br>**l.** MEDICAL EXPENSES FOR OTHER 1 2<br>**m.** TRANSPORT/TRIPS 1 2<br>**n.** OTHER (SPECIFY________) 1 2		415
416	In the past six months, in which of the following places have you saved your money?	**READ ALL OPTIONS** **YES** **NO**<br>BANK/BANK ACCOUNT 1 2<br>SAVINGS ACCOUNT 1 2<br>HOMEBANK/UNDER MATTRESS/HOLE 1 2<br>BOX/CLOSET 1 2<br>WITH A FRIEND 1 2<br>WITH A PARENT OR GUARDIAN 1 2<br>IN A SAVINGS GROUP/CHAMA 1 2<br>SHOPKEEPER 1 2<br>AGRICULTURAL INVESTMENTS 1 2<br>MPESA 1 2<br>OTHER (SPECIFY) 96		416
417	Do you agree or disagree with the following statement: “Only people with a lot of money can save”?	AGREE 1<br>DISAGREE 2		417

SECTION 5: SOCIAL CAPITAL AND NETWORKS

	QUESTIONS	RESPONSES			
501	Are you a member of any social groups or clubs that you have attended within the last one year ? For instance, are you a member of	**502**<br>When is the last time you attended this club?	**503**<br>Was it a girls only club or mixed girls and boys?		501

[READ LIST; IF 'YES' ASK 502 and 503]

1 = Within last 7 days
2 = Within the last month
3 = Within the last one year

		YES	NO		LAST 7 DAYS	LAST MONTH	IN THE LAST ONE YEAR		Girls Only	Mixed
**a.**	Gender club	1	2	**a.**	1	2	3	**a.**	1	2
**b.**	Child rights/Human rights	1	2	**b.**	1	2	3	**b.**	1	2
**c.**	HIV&AIDS/Health life skills/ Guidance & counseling club	1	2	**c.**	1	2	3	**c.**	1	2
**d.**	Girl guides/scouts	1	2	**d.**	1	2	3	**d.**	1	2
**e.**	Sports club	1	2	**e.**	1	2	3	**e.**	1	2
**f.**	Drama or dancing group	1	2	**f.**	1	2	3	**f.**	1	2
**g.**	Church group/Muslim group	1	2	**g.**	1	2	3	**g.**	1	2
**h.**	Any other ______________	1	2	**h.**	1	2	3	**h.**	1	2

[FIRST ASK ALL QUESTIONS FOR MALES AND RECORD ANSWERS IN COLUMN A; THEN RETURN TO ASK ALL QUESTIONS FOR FEMALES AND RECORD ANSWERS IN COLUMN B]

Now I want to ask about your good friends. What we mean by a good friend is someone you can confide in about personal matters and share important information.

	[FRIENDS: 0-80; RECORD 80 IF MORE THAN 80; RECORD 88 IF DON'T KNOW]	A. MALES	B. FEMALES		
**504**	How many good friends do you have who are (MALE/FEMALE)?	[ ][ ]	[ ][ ]<br>**[IF =A= 0 & B=0 → 507]**		**504**
**505**	How many of these (MALE/FEMALE) friends are currently attending school?	[ ][ ]	[ ][ ]<br>**[IF = A=0 & B=0 → 507]**		**505**
**506**	How many of these (MALE/FEMALE) friends are attending your school?	[ ][ ]	[ ][ ]		**506**

SOCIAL ASSETS AND SAFETY NETS

I am going to read you a few more statements and ask if you agree or disagree with them.

**507**	I have many friends in my neighborhood/community	AGREE<br>DISAGREE	1<br>2		**507**
**508**	I feel safe walking around in my neighborhood/community during the day	AGREE<br>DISAGREE	1<br>2		**508**
**509**	I feel safe walking around in my neighborhood/community after dark	AGREE<br>DISAGREE	1<br>2		**509**
**510**	People in my neighborhood/community trust one another	AGREE<br>DISAGREE	1<br>2		**510**
**511**	Sometimes, in my every day life, I feel scared that I will be raped	AGREE<br>DISAGREE	1<br>2		**511**
**512**	I have been touched indecently by a boy or man in the past six months	AGREE<br>DISAGREE	1<br>2		**512**
**513**	I have been robbed in the past six months while in my neighborhood/community	AGREE<br>DISAGREE	1<br>2		**513**
**514**	I have been robbed in the past six months while outside my neighborhood/community	AGREE<br>DISAGREE	1<br>2		**514**

515	In my neighborhood/community, boys or men tease me as I go about my day	AGREE 1<br>DISAGREE 2		515
516	I have a good friend in my community who I can turn to for help if I had a serious problem	AGREE 1<br>DISAGREE 2		516
517	There is a female adult in my community, other than my parents or teacher, whom I can turn to for help if I had a serious problem	AGREE 1<br>DISAGREE 2		517
518	I have a good friend who I meet regularly in my life that I can discuss my problems and joys, and ask questions of.	AGREE 1<br>DISAGREE 2		518
519	There is a female adult, that is not my mother or teacher, who I meet regularly in my life that I can discuss my problems and joys, and ask questions of.	AGREE 1<br>DISAGREE 2		519

520

Q520A Could you count on the following people if you needed money urgently?

[READ THE LIST. IF NO ON Q520A, GO TO NEXT ITEM, SKIP Q520B, Q520C]

Q520B Sex

Q520C Residence/location (CODE)

CODES
1 Resides in my household
2 Resides in this community
3 Resides in neighboring community
4 Resides upcountry elsewhere/ (rural area)

		YES	NO	Male Only	Female Only	Both	Male CODES	Female CODES
A	Parent/guardian	1	2	1	2	3		
B	Sibling	1	2	1	2	3		
C	Relative	1	2	1	2	3		
D	A close friend	1	2	1	2	3		
E	Neighbor	1	2	1	2	3		
F	Boyfriend/husband	1	2	1	2	3		
G	Mentor	1	2	1	2	3		
H	Teacher	1	2	1	2	3		

521

Q521A Would you go to any of the following people if you had a question about your health or your body?

[READ THE LIST. IF NO ON Q521A, GO TO NEXT ITEM, SKIP Q521B, Q521C]

Q521B Sex

Q521C Residence/location (CODE)

1 Resides in my household
2 Resides in this community
3 Resides in neighboring community
4 Resides elsewhere/ upcountry (rural area)

		YES	NO	Male Only	Female Only	Both	Male CODES	Female CODES
							**Add boxes**	
A	Parent / Guardian	**1**	**2**	**1**	**2**	**3**		
B	Sibling	1	2	1	2	3		
C	Relative	1	2	1	2	3		
D	A close friend	1	2	1	2	3		
E	Neighbor	1	2	1	2	3		
F	Boyfriend/husband	1	2	1	2	3		
G	Mentor	1	2	1	2	3		
H	Teacher	1	2	1	2	3		

For each of the following places, please tell me whether you are usually permitted to go to them on your own, only if someone accompanies you, or not at all

522	Local Health Clinic	ON MY OWN 1<br>IF SOMEONE ACCOMPANIES ME 2<br>NOT AT ALL 3<br>NEVER GO THERE 9		522
523	Homes of friends in my neighborhood	ON MY OWN 1<br>IF SOMEONE ACCOMPANIES ME 2		523

		NOT AT ALL 3<br>NEVER GO THERE 9			
524	Youth Group/Girls Group	ON MY OWN 1<br>IF SOMEONE ACCOMPANIES ME 2<br>NOT AT ALL 3<br>NEVER GO THERE 9			524
525	Many different factors can prevent girls from getting medical advice or treatment for themselves. When you are sick and want to get medical advice or treatment, is each of the following a big problem or not?<br><br>**[READ THE LIST]**	BIG PROBLEM<br>YES NO			525
	**a.** Getting permission to go?	**a.** PERMISSION 1 2			
	**b.** Getting money needed for treatment?	**b.** MONEY 1 2			
	**c.** The distance to the health facility?	**c.** DISTANCE 1 2			
	**d.** Having to take transport?	**d.** TRANSPORT 1 2			
	**e.** Not wanting to go alone?	**e.** GO ALONE 1 2			
	**f.** Concern that there may not be a female health provider?	**f.** NO FEMALE PROVIDER 1 2			
	**g.** Concern that there may not be any health provider?	**g.** NO PROVIDER 1 2			
	**h.** Concern that there may be no drugs available?	**h.** NO DRUGS 1 2			
	**i.** Concern about lack of privacy/confidentiality	**i.** LACK OF PRIVACY/CONFIDENTIALITY 1 2			
	**j.** Fear of judgement from the health provider	**j.** JUDGEMENT BY PROVIDER 1 2			
	**k.** Embarrassed to discuss the health issue	**k.** EMBARRASSMENT 1 2			
526	In the past six months, did you go to a health facility to get any type of health services?	YES 1<br>NO 2	**529**		526
527	Which of the following health facilities did you visit in the past six months?<br><br>**[Read the list, Check all that apply]**	PRIVATE CLINIC 1<br>PUBLIC HOSPITAL 2<br>PUBLIC HEALTH CENTER/CLINIC 3<br>DISPENSARY 4<br>CHEMIST/PHARMACY 5<br>MOBILE/OUTREACH SERVICE 6<br>NGO FACILITY 7<br>OTHER (SPECIFY) ….._______________________________ 98			527
528	Please tell me which health services you received during those visits?<br><br>**[RECORD ALL MENTIONED]**	**SERVICE CODES**<br>a. GENERAL HEALTH/ SICKNESS (e.g. malaria, diarrhea, cold) 1<br>b. FAMILY PLANNING 2<br>c. PREGNANCY TEST 3<br>d. ANTENATAL CARE 4<br>e. HIV TESTING 5<br>f. HIV CARE/TREATMENT 6<br>g. STI TESTING (NON-HIV) 7<br>h. STI CARE/TREATMENT (NON-HIV) 8<br>i. CHILD HEALTH/SICKNESS 9<br>j. DELIVERY 10<br>k. POSTNATAL CARE 11<br>l. URINARY TRACT INFECTIONS (UTIs) CARE/TREATMENT 12<br>l. OTHER REPRODUCTIVE HEALTH SERVICES 13<br>m. PHARMACY/VITAMINS/MEDICINE 14<br>o. OTHER (SPECIFY________) 98			528
529	**I want you to think about your most recent weekday, from Monday through Friday, that was not a public holiday or a school holiday and try to recall the different activities you did on that day. Tell me about the different activities you did and how long you spent on them.**				529
	[RECORD HOW MANY HOURS SHE SPENT ON VARIOUS ACTIVITIES DURING THE LAST COMPLETE WEEKDAY – NOT SATURDAY OR SUNDAY OR HOLIDAY .]	**ACTIVITY** — **NUMBER OF MINUTES**<br>**a**. In school [ ]<br>**b**. Unpaid work at home (e.g. cooking, cleaning, washing clothes) [ ]<br>**c.** Unpaid work outside the home (e.g. going to the market, fetching water) [ ]<br>**d.** Paid wage work/ paid work for others/In own business [ ]			

**e.** Leisure time (chatting with friends, drinking tea/coffee with friends, watching TV, reading novels, etc)	[ ]	
**f.** Church/mosque/praying/duksi/madrassa	[ ]	
**g.** Studying/homework	[ ]	
h. Travelling to and from school	[ ]	
i. Sleeping	[ ]	
TOTAL MINUTES [CHECK NOT MORE THAN 1440]	[ ]	

SECTION 6: SELF-EFFICACY, LOCUS OF CONTROL, AND GENDER ATTITUDES

	QUESTIONS	RESPONSES		
	**Generalized Self Efficacy**			
**601**	Now, I am going to read you some statements, please tell me if you agree or disagree with them.			**601**

	AGREE	DISAGREE	DON'T KNOW
**a.** You always manage to solve difficult problems if you try hard enough.	1	2	88
**b**. If someone is against you, you can still find ways to get what you want.	1	2	88
**c**. It is easy for you to focus on your aims and accomplish your goals	1	2	88
**d.** You are confident that you could handle unexpected events very well	1	2	88
**e.** Because of the help you can get, you know how to manage unexpected situations.	1	2	88
**f.** You can solve most problems if you make the necessary effort.	1	2	88
**g**. You can remain calm when facing difficulties because you can rely on your own abilities.	1	2	88
**h.** When you face a problem, you can usually find more than one solution.	1	2	88
**i.** If you are in trouble, you can usually think of a solution.	1	2	88
**j.** You can usually handle any situation that comes your way.	1	2	88

	QUESTIONS	RESPONSES		
**602**	**Self-Efficacy (India Transition Study)**<br>Do you express your opinion to elders in your family? Would you say often, sometimes or never?	OFTEN 1<br>SOMETIMES 2<br>NEVER 3		**602**
**603**	If you disagree with someone, do you usually tell that person that you disagree with him/her? Would you say you always confront, sometimes give a response or always stay quiet?	ALWAYS CONFRONT 1<br>SOMETIMES GIVE RESPONSE 2<br>ALWAYS,STAY QUIET 3		**603**
**604**	Suppose, a boy or man tries to touch you on your private parts, when you did not want. Would you be able to say 'NO' to that person?	YES 1<br>NO 2		**604**
**605**	Do you feel confident about speaking in front of a group of people of your age? Would you say always, sometimes or never?	ALWAYS 1<br>SOMETIMES 2<br>NEVER 3		**605**

606 For each of the following statements, please tell me if you AGREE A LOT, AGREE ALITTLE, DISAGREE ALITTLE OR DISAGREE A LOT **606**

Locus of Control	AGREE A LOT	AGREE ALITTLE	DISAGREE ALITTLE	DISAGREE A LOT	DON'T KNOW
**a.** To a great extent, the important things that happen in my life happen by chance (reverse scored).	1	2	3	4	88
**b.** My success so far in my life is due to my ability and hard work.	1	2	3	4	88
**c.** Whether or not I succeed in the KCPE exam will depend on my actions and what I do.	1	2	3	4	88
**d.** When I succeed it is often due to luck and when I fail it is often due to how powerful other people are (reverse scored).	1	2	3	4	88
**e.** If I succeed in achieving my goals in life it will be because of how well I prepared myself.	1	2	3	4	88
**f.** Most of my problems will go away if I just ignore them (reverse scored)	1	2	3	4	88

GENDER NORMS (GEAS)
For each of the following statements, please tell me if you AGREE A LOT, AGREE ALITTLE, DISAGREE ALITTLE OR DISAGREE A LOT

607	Girls Freedom Versus Lack of Independence	AGREE A LOT	AGREE ALITTLE	DISAGREE ALITTLE	DISAGREE A LOT	DON'T KNOW		607
	**a.** Girls should be as independent as boys	1	2	3	4	88		
	**b.** Girls should not go out with their friends unless an adult is present	1	2	3	4	88		
	**c.** Girls feel they are more limited in what they can do and where they can go than boys	1	2	3	4	88		
	**d.** Girls should have the same chances/opportunities as boys	1	2	3	4	88		
	**e.** Girls should be able to move about as freely as boys	1	2	3	4	88		
	**Girls Responsibilities**							
	**f.** t is as important for girls to do well in school as it is for boys	1	2	3	4	88		
	**g.** Boys and girls should be equally responsible for household chores	1	2	3	4	88		
	**Deference/Proper/Composed**							
	**h.** Girls should keep their thoughts to themselves and not say what they think in public	1	2	3	4	88		
	**i.** Girls are expected to be humble	1	2	3	4	88		
	**j.** Girls should not make too many demands on others	1	2	3	4	88		
	**k.** A girl should say what she thinks even if it hurts someone's feelings	1	2	3	4	88		
	**l.** Girls who argue with their friends in public do not behave as	1	2	3	4	88		
	**Girls are Responsible for Arousing Boys**							
	**m.** Girls should cover up or they will attract unwanted sexual attention	1	2	3	4	88		
	**n.** Girls should be careful about the way they look so they are not seen as trying to seduce men	1	2	3	4	88		
	**o.** It's a girl's fault if boys come onto them	1	2	3	4	88		
	**p.** Girls wear short dresses to get boys' attention	1	2	3	4	88		
	**q.** Girls should be free to dress as they want	1	2	3	4	88		

SECTION 7. MARRIAGE & SEXUAL BEHAVIOR

QUESTIONS		RESPONSES		
701	Have you ever been married?	YES 1<br>NO 2	703	701
702	At what age do you expect to get married?	AGE [10-50] [ ][ ]<br>DON'T KNOW 88<br>DON'T EXPECT TO GET MARRIED 98<br>ALL	705	702
703	At what age did you get married?	AGE [10-20] [ ][ ]<br>DON'T KNOW 88		703
704	Are you currently married, separated, divorced or widowed?	MARRIED/LIVING WITH PARTNER 1<br>SEPARATED 2<br>DIVORCED 3<br>WIDOWED 4		704
705	Have you ever lived with a boyfriend?	YES 1<br>NO 2	707	705
706	At what age did you first start living with a boyfriend?	AGE [10-20] [ ][ ]<br>DON'T KNOW 88		706
707	How old were you when you had sexual intercourse for the very <u>first</u> time?	NEVER HAD SEXUAL INTERCOURSE 0<br>BELOW 7 YEARS 1<br>AGE IN YEARS [7-20] [ ][ ]<br>DON'T KNOW 88	801	707

708	What was your relationship to the first person with whom you had sexual intercourse?<br><br>**[DO NOT READ OPTIONS]**	HUSBAND 1<br>BOYFRIEND 2<br>"SUGAR DADDY" 3<br>CASUAL ACQUAINTANCE 4<br>RELATIVE 5<br>TEACHER 6<br>STRANGER 7<br>SOMEONE ELSE 8		708
709	Was the person you <u>first</u> had sexual intercourse with older than you, younger than you, or about the same age as you?	OLDER 1<br>YOUNGER 2<br>ABOUT THE SAME AGE 3<br>DON'T KNOW/DON'T REMEMBER 88	 **711**<br>**711**<br>**711**	709
710	How many years older than you was this person?	NUMBER OF YEARS OLDER [ ][ ]<br>DON'T KNOW 88		710
711	The <u>first</u> time you had sexual intercourse, was a condom used?	YES 1<br>NO 2<br>DON'T KNOW/DON'T REMEMBER 88		711
712	The first time you had sex did you want to have sex, not want to have sex, or you were unsure if you wanted to have sex?	WANTED TO HAVE SEX 1<br>DID NOT WANT TO HAVE SEX 2<br>UNSURE 3		712
713	Now I would like to ask you some questions about your recent sexual activity. When was the <u>last</u> time you had sexual intercourse?	TODAY/LESS THAN A WEEK AGO 1<br>A WEEK OR MORE AGO 2<br>A MONTH OR MORE AGO 3<br>A YEAR OR MORE AGO 4	 **716**<br>**716**<br>**716**	713
714	In the last week, how many times did you have sex?	TIMES HAD SEX [1-35] [ ][ ]<br>DON'T KNOW 88		714
715	Of those times you had sex in the last week, how many times did you use a condom?<br><br>**[Consistency check: Maximum = previous question]**	TIMES USED CONDOM [1-35] [ ][ ]<br>DID NOT USE CONDOM 77<br>DON'T KNOW 88		715
716	What was your relationship to the <u>last</u> person with whom you had sexual intercourse?	HUSBAND 1<br>BOYFRIEND 2<br>"SUGAR DADDY" 3<br>CASUAL ACQUAINTANCE 4<br>RELATIVE 5<br>TEACHER 6<br>STRANGER 7<br>SOMEONE ELSE 8		716
717	Was the person you <u>last</u> had sexual intercourse with older than you, younger than you, or about the same age as you?	OLDER 1<br>YOUNGER 2<br>ABOUT THE SAME AGE 3<br>DON'T KNOW/DON'T REMEMBER 88	 **720**<br>**720**<br>**720**	717
718	How many years older than you was this person?	NUMBER OF YEARS OLDER [ ][ ]<br>DON'T KNOW 88		718
719	The <u>last</u> time you had sex did you want to have sex, not want to have sex, or you were unsure if you wanted to have sex?	WANTED TO HAVE SEX 1<br>DID NOT WANT TO HAVE SEX 2<br>UNSURE 3		719
720	The <u>last</u> time you had sexual intercourse, was a condom used?	YES 1<br>NO 2<br>DON'T KNOW/DON'T REMEMBER 88		720
721	On average, how often did you use a condom every time you had sexual intercourse with this person in the last one year? Would you say always, sometimes or never?	ALWAYS 1<br>SOMETIMES 2<br>NEVER 3		721
722	If you were interested in obtaining family planning, do you feel it would be easy, difficult or not possible to go to a place where you can obtain family planning?	EASY 1<br>DIFFICULT 2<br>NOT POSSIBLE 3<br>DON'T KNOW 88		722
**FILTER**	**[SKIP Q728 IF Q713 IS A YEAR OR MORE AGO]**			**FILTER**
723	In total, with how many different people have you had sexual	NUMBER OF PARTNERS IN LAST 6 MONTHS [1-35] [ ][ ]		723

	intercourse in the last six months?	DON'T KNOW 88		
724	I will now read a list of different profiles of people and I want you to tell me if you have had sex with such a person in the past year.<br><br>**[READ THE LIST]**	YES NO<br>A. A TOURIST OR FOREIGNER 1 2<br>B. AN EMPLOYER OF YOURS 1 2<br>C. A PERSON 10 OR MORE YEARS OLDER THAN YOU 1 2<br>D. A TEACHER OF YOURS 1 2<br>E. A PERSON WHO PAID YOU FOR SEX 1 2<br>F. A PERSON WHO IS MARRIED TO SOMEONE ELSE 1 2<br>G. NONE OF THE ABOVE 1 2		724
	For the following questions, please think about your own experiences and tell me if you agree or disagree with the following about yourself.			
725	You have not always been able to use condoms when you wanted to.	AGREE 1<br>DISAGREE 2		725
726	You have had sex with a person in exchange for him paying rent for you.	AGREE 1<br>DISAGREE 2		726
727	You have had sex at times when you did not want to.	AGREE 1<br>DISAGREE 2		727
728	In the past six months, have you had sex with someone because you needed or thought you would get any of the following?	YES NO<br>**a.** Food 1 2<br>**b.** Shelter/a place to stay 1 2<br>**c.** School fees 1 2<br>**d.** Sanitary pads 1 2<br>**e.** Money for something else 1 2<br>**f.** Anything else 1 2	 <br><br><br><br>**SPECIFY**<br>**SPECIFY**	728
	Money for something else, (SPECIFY)____			
	Anything else, (SPECIFY)____			

SECTION 8: MENSTRUATION

QUESTIONS		RESPONSES		
801	Have you ever heard of menstruation or the monthly period, which is also known as "getting visitors', 'leaking', or 'kuvudza/ kuvuja'?	YES 1<br>NO 2	 **901**	801
802	From whom or where did you **FIRST** get information on menstruation?<br><br>**MORE THAN ONE ANSWER POSSIBLE. CIRCLE ALL THAT APPLY**	**YES** **NO**<br>MOTHER 1 2<br>GRANDMOTHER 1 2<br>SISTER 1 2<br>AUNT 1 2<br>FRIEND 1 2<br>TEACHER 1 2<br>OTHER FAMILY MEMBER 1 2<br>NGO/CBO 1 2<br>HEALTH WORKER/CLINICIAN/DOCTOR 1 2<br>MEDIA 1 2<br>OTHER (SPECIFY________) 1 2		802
	If you said other, please specify	____		
803	Have you ever menstruated or gotten your monthly period?	YES 1<br>NO 2	 **901**	803
804	Did you know about menstruation before experiencing it **for the first time**?	YES 1<br>NO 2<br>NO ANSWER 96		804
805	At what age did you begin getting your menstrual period?	AGE<br>[4-20]<br>DON'T KNOW 88<br>**[CONSISTENCY CHECK WITH CURRENT AGE]**		805
806	When you first got your period, did you tell anyone about it?	YES 1<br>NO 2	 **808**	806
807	Who did you tell?	**YES** **NO**<br>MOTHER 1 2<br>GRANDMOTHER 1 2		807

No.	Question	Response	Code	Code	Skip	No.
	**MORE THAN ONE ANSWER POSSIBLE. CIRCLE ALL THAT APPLY**	SISTER	1	2		
		AUNT	1	2		
		FRIEND	1	2		
		TEACHER	1	2		
		HEALTH FPROFESSIONAL	1	2		
		OTHER (SPECIFY________)		98		
**808**	What is the main method that you **currently** use to manage yourself during your period at home, and at school?		<u>**AT SCHOOL**</u>	<u>**AT HOME**</u>		**808**
		Sanitary pads (disposable)	1	1		
		Tampon	2	2		
		Tissue paper/ other paper	3	3		
		Cotton wool	4	4		
		Old pieces of cloth	5	5		
		New peieces of cloth	6	6		
		Sanitary pads (reusable)	7	7		
		Matress pieces	8	8		
		Other (SPECIFY_______)	98	98		
**809**	Where do you usually get the materials you just mentioned?		**YES**	**NO**		**809**
	**[CHECK ALL THAT APPLY]**	I BUY THEM	1	2		
		FROM MY HOME	1	2	**811**	
		GIVEN BY MOTHER	1	2	**811**	
		GIVEN BY OTHER RELATIVE	1	2	**811**	
		FROM SCHOOL	1	2	**811**	
		FROM FRIENDS	1	2	**811**	
		GIVEN BY AN NGO/CBO	1	2	**811**	
		BOYFRIEND	1	2	**811**	
		OTHER (SPECIFY_______)		98	**811**	
	<u>If you said other, please specify</u>	______________________________				
**810**	Where do you get the money?		**YES**	**NO**		**810**
	**[CHECK ALL THAT APPLY]**	From parents	1	2		
		Job (specify job)	1	2		
		Own savings	1	2		
		Boyfriend	1	2		
		Other family member	1	2		
		Other (SPECIFY_______)	1	2		
	If you said other, please specify	______________________________				
**811**	Apart from the main method you mentioned, what other methods do you use to manage yourself during your period?		**YES**	**NO**		**811**
	**[CHECK ALL THAT APPLY]**	Sanitary pads (disposable)				
		Tampon	1	2		
		Tissue paper/ other paper	1	2	**IF NO ON ALL, SKIP 813**	
		Cotton wool	1	2		
		Old pieces of cloth	1	2		
		New pieces of cloth	1	2		
		Sanitary towel (reusable)	1	2		
		Mattress pieces	1	2		
		Other (SPECIFY_________)	1	2		
				98		
	If you said other, please specify	______________________________				
**812**	Of the methods you currently use, which do you prefer the most?	Sanitary towel (disposable)		1		**812**
		Tampon		2		
		Tissue paper/ other paper		3		
		Cotton wool		4		
		Old pieces of cloth		5		
		New pieces of cloth		6		
		Sanitary towel (reusable)		7		
		Mattress pieces		8		
		Other (SPECIFY_____)		98		
	I am now going to ask several more questions about how you manage your period at school, and how you manage your period at home.					
**813**	How do you usually deal with pain or cramps during your period?		<u>**AT SCHOOL**</u> **YES NO**	<u>**AT HOME**</u> **YES NO**		**813**
	**[Check all that apply]**	Does not do anything	1 2	1 2		
		Takes pain medication	1 2	1 2		
		Goes home/does not go to school	1 2	1 2		
		Goes to hospital	1 2	1 2		
		Uses alternative method. (SPECIFY_____)	1 2	1 2		
		Does not have pain/cramps	1 2	1 2		
**814**	Do you ever change your pad/other materials?		<u>**AT SCHOOL**</u>	<u>**AT HOME**</u>		**814**
	**[If never used pads ask about other materials used]**	YES	1	1		
		NO	2	2		
		N/A	97	97		
			**[IF NO FOR BOTH]**		**821**	
**815**	How often are you able to change your pad/other materials in private? Would you say always, sometimes, rarely, or never?		<u>**AT SCHOOL**</u>	<u>**AT HOME**</u>		**815**
		Always	1	1		
		Sometimes	2	2		

		Rarely<br>Never<br>You did not need to change	3<br>4<br>8	3<br>4<br>8		
816	Where do you usually change your pad/other material?	 Toilet<br>Classroom/room at home<br>Changed outside<br>Other (SPECIFY_____)<br>Not Applicable	**AT SCHOOL**<br>1<br>2<br>3<br>98<br>97	**AT HOME**<br>1<br>2<br>3<br>98<br>97		816
817	Are you able to clean your hands after changing?	 YES<br>NO<br>**[IF NO FOR BOTH]**	**AT SCHOOL**<br>1<br>2	**AT HOME**<br>1<br>2	 <br><br>**819**	817
818	What do you use to clean your hands after changing?	 <br>Soapy water<br>Soap<br>Water<br>Wet cloth<br>Dry cloth<br>Other<br>None	**AT SCHOOL**<br>YES NO<br>1 2<br>1 2<br>1 2<br>1 2<br>1 2<br>1 2<br>1 2	**AT HOME**<br>YES NO<br>1 2<br>1 2<br>1 2<br>1 2<br>1 2<br>1 2<br>1 2		818
819	How often are you able to dispose of your dirty pads/other material? Would you say always, sometimes, rarely or never?	 ALWAYS<br>SOMETIMES<br>NEVER<br>RARELY<br>N/A	**AT SCHOOL**<br>1<br>2<br>3<br>4<br>8	**AT HOME**<br>2<br>2<br>3<br>4<br>8		819
820	Where do you dispose your dirty pads/other material?<br><br>**[Check all that apply]**	 <br>INSIDE LATRINE<br>LATRINE FLOOR<br>BIN<br>DROP IN FIELD<br>BURN IN FIELD<br>TAKE TO SCHOOL/HOME<br>DROP ON WAY TO SCHOOL/HOME<br>RIVER, LAKE, POND<br>OTHER (SPECIFY________)	**AT SCHOOL**<br>YES NO<br>1 2<br>1 2<br>1 2<br>1 2<br>1 2<br>1 2<br>1 2<br>1 2	**AT HOME**<br>YES NO<br>1 2<br>1 2<br>1 2<br>1 2<br>1 2<br>1 2<br>1 2<br>1 2<br>98		820
821	Did you have a monthly period every month for the past three months?	YES<br>NO		1<br>2		821
822	What was your date of your last monthly period?	Date of period<br>**Note: First date when it started**		[ ][ ][ ]		822
823	How many days did your recent period last?	Total days		[ ][ ]		823
824	Did you have pain or cramps in this recent period?	YES<br>NO		1<br>2		824
825	Have you ever had any blood leak onto your body or clothes when you were at school?	YES<br>NO		1<br>2	 **FILTER**	825
826	If so, how often have you experienced this?<br><br>**READ LIST**	ALWAYS (EVERY PERIOD)<br>SOMETIMES<br>RARELY (ONCE OR TWICE)<br>NEVER		1<br>2<br>3<br>4		826
827	What did you use at school to wash/clean off the blood ?	SOAPY WATER<br>SOAP AND WATER<br>WATER<br>WET CLOTH<br>DRY CLOTH<br>NONE<br>OTHER (SPECIFY_________)		1<br>2<br>3<br>4<br>5<br>6<br>98		827
**FILTER**	**[IF USED PADS IN Q808 OR Q811, SKIP TO 829]**				**829**	**FILTER**
828	Have you ever used sanitary pads?	YES<br>NO		1<br>2	 **834**	828
829	On average, how many pads do you use per month when you have your period?	Total number		[ ][ ]		829

830	If you have some remaining, do you sometimes share the pads with anyone else?	YES 1<br>NO 2	 832	830
831	If you share, who do you share with?<br><br>**[Check all that apply]**	YES NO<br>MOTHER 1 2<br>SISTER 1 2<br>CLASSMATES 1 2<br>FRIENDS WHO ARE NOT CLASSMATES 1 2<br>OTHER RELATIVE 1 2<br>TEACHER 1 2<br>OTHER (SPECIFY_________) 1 2		831
	If you said other, please specify			
832	Do you feel like you have enough pads to manage your period comfortably?	YES 1<br>NO 2		832
833	About how frequently do you change your pads in a day/24hours?	Once a day 1<br>Twice a day (Once every 12hours) 2<br>Three times a day (Once every 8hours) 3<br>More than three times a day / Once every 7hours or less 4<br>None 5<br>Other (SPECIFY_________) 98		833
834	Did you receive sanitary pads at school during this/the last school term?	YES 1<br>NO 2	 838	834
835	If yes, were you just given the pads or did you have to ask for them?	Just given 1<br>Asked for them 2		835
836	The last time you received sanitary pads at school, did you receive three or more packets, two packets, one packet or less than a	Three or more packets 1<br>Two packets 2<br>One packet 3<br>Less than a packet 4		836
837	How often did you receive pads at school during the last school term?	Once or twice per term 1<br>Three or more times per term 2		837
838	Tell me how much you think the following statements are true for you? For each statement below, please check whether you Agree, Disagree or you're Not Sure.			838

	Agree	Disagree	Not sure	Don't know
**a.** I feel ashamed of my body when I have my period	1	2	3	88
**b.** I'm uncomfortable in my body when I have my period	1	2	3	88
**c.** It's important that I keep my period secret from anyone	1	2	3	88
**d.** I feel proud that I have my periods	1	2	3	88
**e.** I wish I could never have periods	1	2	3	88
**f.** A girl can engage in sports activities during her periods	1	2	3	88
**g.** It is easy to concentrate in class while on my periods	1	2	3	88
**h.** Periods are an illness	1	2	3	88
**i.** A girl is unclean when she is on her period	1	2	3	88
**j.** Boys can tell when girls are having their periods	1	2	3	88
**k.** Menstruation is a normal process for girls	1	2	3	88
**l.** One should not bathe during menstruation	1	2	3	88
**m.** All girls who have ever had a period are at risk of becoming pregnant if they have sex	1	2	3	88
**n.** A girl should not feel embarrassed if she stains her dress at school when on her periods	1	2	3	88

839	Are there any activities that you are forbidden by anyone to engage in during your periods?	Yes 1<br>No 2	 841	839
840	If yes, what activities?<br><br>**[CHECK ALL THAT APPLY]**	YES NO<br>Going to school 1 2<br>Going to church/mosque 1 2<br>Going to market 1 2<br>Cooking for the family 1 2<br>Interacting with other family members 1 2<br>Interacting with neighbors 1 2<br>Interacting with men/boys 1 2<br>Playing games/sports 1 2		840

		Fasting 1 2<br>Other (SPECIFY________) 1 2		
	If you said other, please specify			
841	Are there any activities that you **choose** not to engage in during your periods?	YES 1<br>NO 2	843	841
842	If yes, what activities?<br>**[CHECK ALL THAT APPLY]**	YES NO<br>Going to school 1 2<br>Going to church/mosque 1 2<br>Going to market 1 2<br>Cooking for the family 1 2<br>Interacting with other family members 1 2<br>Interacting with neighbors 1 2<br>Interacting with men/boys 1 2<br>Playing games/sports 1 2<br>Fasting 1 2<br>Other (SPECIFY_________) 98		842
	If you said other, please specify	If you said other, please specify		
843	Within the past year, has anyone teased you about your period?	Yes<br>No	901	843
844	Who has teased you about your period?<br>**[CHECK ALL THAT APPLY**	**YES NO**<br>Brother 1 2<br>Sister 1 2<br>Other male relative 1 2<br>Other female relative 1 2<br>Male school mate 1 2<br>Female school mate 1 2<br>Other male friend 1 2<br>Other female friend 1 2<br>Teacher 1 2<br>Other (SPECIFY) ________________________ 1 2		844
	If you said other, please specify			

SECTION 9: REPRODUCTIVE HEALTH KNOWLEDGE AND CONTRACEPTION

901	**Please tell me if the following statements are true or false?**<br>Can pregnancy occur after kissing or hugging?	TRUE 1<br>FALSE 2<br>DON'T KNOW 88<br>NO RESPONSE 96		901
902	Is it possible for a woman get pregnant on the very **first time** she has sexual intercourse?	TRUE 1<br>FALSE 2<br>DON'T KNOW 88<br>NO RESPONSE 96		902
903	Girls undergo pubertal changes. Can you please tell me, what are the physical changes that a girl experiences in transition from childhood to adolescence?<br>**[CHECK ALL THAT APPLY]**	YES NO<br>Hair grows in different parts of body 1 2<br>Breasts develop 1 2<br>Acne appears 1 2<br>Menstruation starts 1 2<br>Rapid physical development 1 2<br>Voice Changes 1 2<br>Don't know 88		903
904	From one menstrual period to the next, are there certain days when a woman is more likely to become pregnant if she has sexual intecourse?	YES 1<br>NO 2<br>DON'T KNOW 88	 906<br>906	904
905	Is this time just before her period begins, during her period, right after her period has ended, or two weeks after her period?	JUST BEFORE HER PERIOD BEGINS 1<br>DURING HER PERIOD 2<br>RIGHT AFTER HER PERIOD ENDS 3<br>HALFWAY BETWEEN TWO PERIODS 4<br>OTHER(SPECIFY_______________) 98		905
906	**Please tell me if the following statements are true or false?**<br>Menstruation typically occurs once a month?	TRUE 1<br>FALSE 2<br>DON'T KNOW 88		906
907	It is healthy to have some variation in vaginal discharge throughout your menstrual cycle	TRUE 1<br>FALSE 2<br>DON'T KNOW 88		907

908	Women and girls can get vaginal infections even if they have never had sex	TRUE 1<br>FALSE 2<br>DON'T KNOW 88		908
909	Women and girls can prevent vaginal infections by wiping their private area from the front to the back.	TRUE 1<br>FALSE 2<br>DON'T KNOW 88		909
910	If a girl gets pregnant in her teenage years, what are the potential issues she might have to face?<br><br>**[CHECK ALL THAT APPLY]**	YES NO<br>No problem 1 2<br>Health risk to mother 1 2<br>Health risk to baby 1 2<br>Education might get stopped 1 2<br>May not be able to continue working 1 2<br>May be forced to get married 1 2<br>Others (SPECIFY_____) 1 2<br>Don't know 1 2		910
	If you said other, please specify			
911	What is the legal age for a girl to get married in Kenya?	At age [ ][ ]<br>Don't Know 88		911
912	What are the disadvantages of marrying at an early age (before age 18) for girls?<br><br>**[CHECK ALL THAT APPLY]**	YES NO<br>Risk to child at birth 1 2<br>Risk to Mother at deliver 1 2<br>Immature and incapable of raising children 1 2<br>Immature and incapable of running household 1 2<br>Unable to complete education 1 2<br>Ill health/Depression 1 2<br>Other 1 2<br>Don't know 88		912
	If you said other, please specify ________________________________			
913	Now, I am going to read you some statements, please tell me if you agree or disagree with them.			913

	AGREE	DISAGREE	DON'T KNOW
**a**. A girl has the right to seek and recieve information related to sexuality	1	2	88
**b**. A girl/woman has the right to choose who she will marry	1	2	88
**c.** A girl/woman has the right to decide whether or not to have children	1	2	88
**d.** A girl has a right to refuse to have sex	1	2	88
**e.**A girl has a right to refuse to enter into a forced marriage	1	2	88
**f.** A girl/woman has the right to decide when to have children	1	2	88
**g**. A girl my age has a right to know about family planning and contraceptives	1	2	88
h. A girl/woman has the right to insist on the use of contraceptives/family planning with her husband/partner	1	2	88

914 Now I would like to talk about family planning – the various ways or methods that a couple can use to delay or avoid a pregnancy.

Which ways or methods have you heard about?

[FOR METHODS NOT MENTIONED SPONTANEOUSLY, ASK:

Have you heard of (METHOD)?]

If NO to 914 (a - i), skip to 915

915
Have you ever used [METHOD]?

	914	915
**a.** PILL: Women can take a pill every day to avoid becoming pregnant.	YES, SPONT 1<br>YES, RECOG 2<br>NO 3	YES 1<br>NO 2
**b.** IUD: Women can have a loop or coil placed inside them by a doctor or a nurse.	YES, SPONT 1<br>YES, RECOG 2<br>NO 3	YES 1<br>NO 2
**c.** INJECTABLES: Women can have an injection by a health provider that stops them from becoming pregnant for one or more months.	YES, SPONT 1<br>YES, RECOG 2<br>NO 3	YES 1<br>NO 2
**d.** IMPLANTS: Women can have several small rods placed in their upper arm by a doctor or nurse which can prevent	YES, SPONT 1<br>YES, RECOG 2	YES 1<br>NO 2

	their upper arm by a doctor or nurse which can prevent pregnancy for one or more years.	NO 3			
	**e.** MALE CONDOM: Men can put a rubber sheath on their erect penis before sexual intercourse.	YES, SPONT 1<br>YES, RECOG 2<br>NO 3	YES 1<br>NO 2		
	**f.** FEMALE CONDOM: Women can place a thin, transparent rubber in their vagina before sexual intercourse.	YES, SPONT 1<br>YES, RECOG 2<br>NO 3	YES 1<br>NO 2		
	**g.** RHYTHM METHOD: Every month that a woman is sexually active she can avoid pregnancy by not having sexual intercourse on the days of the month she is most likely to get pregnant.	YES, SPONT 1<br>YES, RECOG 2<br>NO 3	YES 1<br>NO 2		
	**h.** WITHDRAWAL: Men can pull out their penis from the vagina before ejaculation.	YES, SPONT 1<br>YES, RECOG 2<br>NO 3	YES 1<br>NO 2		
	**i.** EMERGENCY CONTRACEPTION: As an emergency measure, women can take pills up to three days after having unprotected sex to avoid getting pregnant. These pills are also called "morning-after pills".	YES, SPONT 1<br>YES, RECOG 2<br>NO 3	YES 1<br>NO 2		
**FILTER**	**CHECK Q707. IF NEVER HAD SEX, SKIP TO 922**				**FILTER**
**916**	Are you currently doing something or using any method to delay or avoid getting pregnant?	YES 1<br>NO 2<br>CURRENTLY PREGNANT 3		 **919**<br>**919**	**916**
**917**	Which method are you using?<br><br>**[RECORD ALL MENTIONED]**	YES NO<br>**a.** PILL 1 2<br>**b.** IUD 1 2<br>**c.** INJECTABLES 1 2<br>**d.** IMPLANTS 1 2<br>**e.** MALE CONDOM 1 2<br>**f.** FEMALE CONDOM 1 2<br>**g.** RHYTHM METHOD 1 2<br>**h.** WITHDRAWAL 1 2<br>**i.** BREASTFEEDING/ LACTATIONAL AMENORRHEA 1 2<br>**j.** EMERGENCY CONTRACEPTIVES 1 2<br>**k.** ABSTINENCE 1 2<br>**l.** OTHER (SPECIFY_________) 1 2		 <br><br><br><br><br><br><br>**919**<br>**919**<br><br>**919**<br>**919**	**917**
**918**	**[ASK IF ANSWER YES TO PILL, IUD, INJECTABLES, IMPLANTS, MALE CONDOM, EMERGENCY CONTRACEPTIVES OR FEMALE CONDOM IN 723]**<br>Where did you obtain [CURRENT METHOD] the last time?	PRIVATE CLINIC 1<br>PUBLIC HOSPITAL 2<br>PUBLIC HEALTH CENTER/CLINIC 3<br>DISPENSARY 4<br>CHEMIST/PHARMACY 5<br>MOBILE/OUTREACH SERVICE 6<br>NGO FACILITY 7<br>OTHER (SPECIFY) ….. ______________________________ 98		**ALL**<br>**922**	**918**
**919**	Do you know of a place where you can obtain a method of family planning?	YES 1<br>NO 2		 **922**	**919**
**920**	Where is that?<br>Any other place?<br><br>**[RECORD ALL MENTIONED]**	PRIVATE CLINIC 1<br>PUBLIC HOSPITAL 2<br>PUBLIC HEALTH CENTER/CLINIC 3<br>DISPENSARY 4<br>CHEMIST/PHARMACY 5<br>MOBILE/OUTREACH SERVICE 6<br>NGO FACILITY 7<br>OTHER (SPECIFY) ….. ______________________________ 98			**920**
**922**	Have you talked about reproductive/sexual health issues with your MOTHER/FEMALE GUARDIAN in the last 6 months?	NO 1<br>YES 2<br>N/A 9		**925**<br><br>**925**	**922**
**923**	Which of the following reproductive/sexual health issues have you discussed?<br><br>**[READ LIST; CHECK ALL THAT APPLY]**	**YES NO**<br>COMMUNICATION 1 2<br>SELF-ESTEEM 1 2<br>PREGNANCY 1 2<br>PUBERTY/HOW BOYS AND GIRLS MATURE 1 2<br>HIV/AIDS 1 2<br>MARRIED LIFE/ROLES IN FAMILY 1 2			**923**

		MENSTRUATION 1 2<br>CONTRACEPTIVES/FAMILY PLANNING 1 2<br>ABSTINENCE/AVOIDING SEX 1 2<br>HEALTH AND HYGIENE 1 2		
924	Which other reproductive/sexual health issue did you discuss? (**SPECIFY**)	____________________ NONE= 0		924
925	Have you talked about reproductive/sexual health issues with your FATHER /MALE GUARDIAN in the last 6 months?	NO 1<br>YES 2<br>N/A 9	928<br><br>928	925
926	Which of the following reproductive/sexual health issues have you discussed?<br><br>**[READ LIST; CHECK ALL THAT APPLY]**	**YES NO**<br>COMMUNICATION 1 2<br>SELF-ESTEEM 1 2<br>PREGNANCY 1 2<br>PUBERTY/HOW BOYS AND GIRLS MATURE 1 2<br>HIV/AIDS 1 2<br>MARRIED LIFE/ROLES IN FAMILY 1 2<br>MENSTRUATION 1 2<br>CONTRACEPTIVES/FAMILY PLANNING 1 2<br>ABSTINENCE/AVOIDING SEX 1 2<br>HEALTH AND HYGIENE 1 2		926
927	Which other reproductive/sexual health issue did you discuss? (**SPECIFY**)	____________________ NONE= 0		927
928	Sometimes women experience a bad smelling abnormal genital discharge. During the last 12 months, have you had a bad smelling abnormal genital discharge?	YES 1<br>NO 2<br>DON'T KNOW 88		928
929	Sometimes women experience pain when urinating, together with a frequent need to urinate. During the last 12 months, have you had pain when urinating together with a frequent need to urinate?	YES 1<br>NO 2<br>DON'T KNOW 88		929
930	Sometimes women have a genital sore or genital ulcer. During the last 12 months, have you had a genital sore or genital ulcer?	YES 1<br>NO 2<br>DON'T KNOW 88		930
	Check 922, 923 & 924. IF NO SYMPTOMS, GO TO FILTER		FILTER	
931	Did you seek treatment for these symptoms?	YES 1<br>NO 2	933	931
932	Why didn't you seek treatment for these symptoms?	1 = Didn't know where to go 1<br>2 = No money for treatment 2<br>3 = Facility too far 3<br>4 = Symptoms cleared up by themselves 4<br>5 = Unfriendly staff 5<br>6 = Other (SPECIFY__ ____________________ 98	FILTER<br>FILTER<br>FILTER<br>FILTER<br>FILTER<br>FILTER	932
933	From where did you seek advice or treatment the last time you had these symptoms?	PRIVATE CLINIC/HOSPITAL 1<br>PUBLIC HOSPITAL 2<br>PUBLIC HEALTH CENTER/CLINIC 3<br>DISPENSARY 4<br>CHEMIST/PHARMACY 5<br>MOBILE/OUTREACH SERVICE 6<br>NGO FACILITY 7<br>OTHER (SPECIFY) ….. ____________________ 98		933

SECTION 10: PREGNANCY AND BIRTHS

FILTER	**[IF NEVER HAD SEX) → Q1012]**		1012	FILTER
QUESTIONS		RESPONSES		Q
1001	Now I would like to ask you about all the births you have had during your life. Have you ever given birth?	YES 1<br>NO 2	1003	1001
1002	Have you ever given birth to a boy or girl who was born alive but later died?	YES 1<br>NO 2	 1005	1002
1003	How many births have you had <u>in total</u> during your life?	TOTAL BIRTHS [1-5] ☐		1003

1005	How old were you when you first gave birth/had a miscarriage/still birth	Age<br>Don't Know	☐☐<br>88<br>[1-19]		1005
1004	Have you ever had a pregnancy that miscarried, or ended in a still birth?	YES<br>NO	1 ☐<br>2 88<br>[1-19]		1004
1006	Are you pregnant now?	YES<br>NO<br>UNSURE/DON'T KNOW	1<br>2<br>88	 F3<br>F3	1006
1007	How many months pregnant are you?<br><br>**[RECORD NUMBER OF COMPLETED MONTHS]**	MONTHS<br>[0-10]<br>DON'T KNOW	☐☐<br><br>88		1007
F3	**[IF <u>ALL</u> OF 1001, 1002, 1005, AND 1006 = NO → 1012]**				F3
1008	When you found out you were pregnant for the very first time, were you still attending school?	YES<br>NO	1<br>2		1008
1009	When you found out you were pregnant for the very first time, were you single, engaged or married?	SINGLE<br>ENGAGED<br>MARRIED	1<br>2<br>3		1009
1010	At the time you first became pregnant, did you want to become pregnant <u>then</u>, did you want to wait until <u>later</u>, or did you <u>not want</u> the pregnancy at all?	THEN<br>ONE TO TWO YEARS LATER<br>THREE OR MORE YEARS LATER<br>NOT AT ALL	1<br>2<br>3<br>4		1010
1011	Sometimes a girl becomes pregnant when she does not want to be. Have you ever been pregnant when you did not want to be?	YES<br>NO	1<br>2		1011
1012	Now I have a question about the future. Would you like to have (a/another) child, or would you prefer not to have any (more) children?	YES<br>NO<br>DON'T KNOW	1<br>2<br>88	 1014	1012
1013	At what age would you like to have (a/another) child?	Age<br>Don't Know	☐☐<br>88<br>[1-50]		1013
1014	If you became pregnant now, how happy would you be? Would you say, very happy, somewhat happy, or not happy at all?	VERY HAPPY<br>SOMEWHAT HAPPY<br>NOT HAPPY AT ALL	1<br>2<br>3		1014

SECTION 11: HIV/AIDS AND OTHER STIs

	QUESTIONS	RESPONSES			
1101	Now I would like to talk about something else. Have you ever heard of an illness called AIDS?	YES<br>NO	1<br>2	 1117	1101
1102	Can people reduce their chances of getting the AIDS virus by having just one uninfected sex partner who has no other sex partners?	YES<br>NO<br>DON'T KNOW	1<br>2<br>88		1102
1103	Can people get the AIDS virus from mosquito bites?	YES<br>NO<br>DON'T KNOW	1<br>2<br>88		1103
1104	Can people reduce their chance of getting the AIDS virus by using a condom every time they have sex?	YES<br>NO<br>DON'T KNOW	1<br>2<br>88		1104
1105	Can people get the AIDS virus by sharing food with a person who has AIDS?	YES<br>NO<br>DON'T KNOW	1<br>2<br>88		1105
1106	Can people reduce their chance of getting the AIDS virus by not having sexual intercourse at all?	YES<br>NO<br>DON'T KNOW	1<br>2<br>88		1106
1107	Can people get the AIDS virus because of witchcraft or other supernatural means?	YES<br>NO<br>DON'T KNOW	1<br>2<br>88		1107
1108	Is it possible for a healthy-looking person to have the AIDS virus?	YES	1		1108

	Is it possible for a healthy looking person to have the AIDS virus?	NO 2<br>DON'T KNOW 88		
**1109**	Can the virus that causes AIDS be transmitted from a mother to her baby:<br><br>**a.** During pregnancy?<br>**b.** During delivery?<br>**c.** By breastfeeding?	YES / NO / DK<br>**a.** DURING PREGNANCY 1 / 2 / 88<br>**b.** DURING DELIVERY 1 / 2 / 88<br>**c.** BREASTFEEDING 1 / 2 / 88		**1109**
**1110**	Do you think your risk of getting infected with HIV is low, medium or high, or do you have no risk at all?	LOW 1<br>MEDIUM 2<br>HIGH 3<br>NO RISK 4<br>ALREADY INFECTED 5<br>DON'T KNOW 88	 <br><br><br>**1115**	**1110**
**1111**	I don't want to know the results, so please do not tell me, but have you ever been tested to see if you have the AIDS virus?	YES 1<br>NO 2	 **1115**	**1111**
**1112**	When was the last time you were tested?	LESS THAN 6 MONTHS AGO 1<br>LESS THAN 12 MONTHS AGO 2<br>12 - 23 MONTHS AGO 3<br>2 OR MORE YEARS AGO 4		**1112**
**1113**	The last time you had the test, did you yourself ask for the test, was it offered to you and you accepted, or was it required?	ASKED FOR THE TEST 1<br>OFFERED AND ACCEPTED 2<br>REQUIRED 3		**1113**
**1114**	I don't want to know the results, so please don't tell me, but did you get the results of the test?	YES 1<br>NO 2	**1116**<br>**1116**	**1114**
**1115**	Do you know of a place where people can go to get tested for the AIDS virus?	YES 1<br>NO 2		**1115**
**1116**	Do you personally know someone who has or is suspected to have the AIDS virus?	YES 1<br>NO 2		**1116**
	**Sexually Transmitted Infections/Diseases**			
**1117**	Besides HIV, do you know of any other diseases that can be transmitted through sexual intercourse?	YES 1<br>NO 2<br>NO RESPONSE 96	 **1201**<br>**1201**	**1117**
**1118**	Can you describe any symptoms of STIs in women?<br><br>**DO NOT READ THE LIST. MORE THAN ONE ANSWER IS POSSIBLE. CIRCLE ALL THAT APPLY**	YES / NO<br>A. Abdominal pain 1 / 2<br>B. Genital discharge 1 / 2<br>C. Foul-smelling discharge 1 / 2<br>D. Burning pain on urination 1 / 2<br>E. Genital ulcers/sores 1 / 2<br>F. Genital warts 1 / 2<br>G. Swellings in the groin area 1 / 2<br>H. Itching 1 / 2<br>I. Other (SPECIFY) ____________________ 1 / 2<br>J. Don't know 1 / 2		**1118**
**1119**	Can you describe any symptoms of STIs in men?<br><br>**DO NOT READ THE LIST. MORE THAN ONE ANSWER IS POSSIBLE. CIRCLE ALL THAT APPLY.**	YES / NO<br>A. Discharge from the penis 1 / 2<br>B. Discharge from the anus 1 / 2<br>C. Burning pain on urination 1 / 2<br>D. Genital ulcers/sores 1 / 2<br>E. Swellings in the groin area 1 / 2<br>F. Can't retract the foreskin 1 / 2<br>G. Ulcer/sores on the anus 1 / 2<br>H. Ulcers/sores on the penis 1 / 2<br>I. Ulcers/sores in the throat 1 / 2<br>J. Lower abdominal pain 1 / 2<br>K. Other (SPECIFY)….. _______________ 1 / 2<br>L. Don't know 1 / 2		**1119**
**1120**	Now I will read you some statements about STIs/STDs and I will want you to tell me whether you Agree or Disagree with each of them.			**1120**
	One can have STD/STIs without showing any symptoms	AGREE 1<br>DISAGREE 2<br>DON'T KNOW 88		
	All STIs/STDs can only be transmitted via sexual intercourse	AGREE 1<br>DISAGREE 2<br>DON'T KNOW 88		

	Condoms protect against most STDs/STIs	AGREE 1<br>DISAGREE 2<br>DON'T KNOW 88		
1121	Have you had or suspected that you had an STI in the past 6 months?	YES 1<br>NO 2<br>NO RESPONSE 96	 1201<br>1201	1121
1122	Did you seek treatment for the STI?	YES 1<br>NO 2	1124	1122
1123	Why didn't you seek treatment for the STI?	1 = Didn't know where to go 1<br>2 = No money for treatment 2<br>3 = Facility too far 3<br>4 = Symptoms cleared up by themselves 4<br>5 = Unfriendly staff 5<br>6 = Self medicated 6<br>7 = Other (SPECIFY__ ____________________ 98	 ALL<br>1201	1123
1124	From where did you seek advice or treatment the last time you had the STI?	PRIVATE CLINIC 1<br>PUBLIC HOSPITAL 2<br>PUBLIC HEALTH CENTER/CLINIC 3<br>DISPENSARY 4<br>CHEMIST/PHARMACY 5<br>MOBILE/OUTREACH SERVICE 6<br>NGO FACILITY 7<br>OTHER (SPECIFY) ….. ______________________________ 98		1124

SECTION 12: GBV/DOMESTIC VIOLENCE

QUESTIONS		RESPONSES				
1201	I am going to read a series of statements about marriage and I want you to tell me if you agree or disagree with the statement.	AGREE	DISAGREE	DON'T KNOW		1201
	**a.** If a young woman in this community were age 25 and unmarried, people would respect her.	1	2	88		
	**b.** It is OK for a man to cook for his family.	1	2	88		
	**c.** Polygamous marriages are part of your culture, so they should continue.	1	2	88		
	**d.** It is better if a girls' family arranges her marriage, than her choosing herself.	1	2	88		
	**e.** If a man doesn't hit his wife, it means he doesn't love her.	1	2	88		
	**f.** It is a man's right to have sex with his wife whenever he wants.	1	2	88		
	**g.** A wife should be able to refuse her husband sex.	1	2	88		
	**h.** If a husband and wife disagree on using family planning, the husband's opinion should come first	1	2	88		
	**i**. Girls my age would become promiscuous if they knew where to get family planning	1	2	88		

1202 **ATTITUDES TOWARDS GENDER VIOLENCE** 1202

I am now going to ask you to think about several situations involving a husband and a wife. When you think about these questions, please keep in mind that we are asking for your opinion about each situation, not your own personal experience.

Sometimes a husband is annoyed or angered by things that his wife does. In your opinion, is a husband justified in hitting or beating his wife in the following situations:

Question		Response	YES	NO
**a.** If she goes out without telling him?	**a.**	GOES OUT	1	2
**b.** If she neglects the children?	**b.**	NEGLECT CHILDREN	1	2
**c.** If she argues with him?	**c.**	ARGUES	1	2
**d.** If she refuses to have sex with him?	**d.**	REFUSES SEX	1	2
**e.** If she burns the food?	**e.**	BURNS FOOD	1	2

1203	Sometimes children feel unsafe or threatened while at school, or	Often 1		1203

	when they are going to or from school. For example, afraid of being attacked, bullied or abused. How often has this happened to you; often, sometimes or never?	Sometimes 2<br>Never 3<br>Don't know 88<br>Refused 96	 **1206**<br>**1206**<br>**1206**	
**1204**	Who makes you feel unsafe or threatened at school or when going to/from school (you do not have to say their name)?<br><br>**(CHECK ALL THAT APPLY)**	YES NO<br>A male teacher 1 2<br>A female teacher 1 2<br>A male students 1 2<br>A female students 1 2<br>A male who is not a student at your school 1 2<br>Other adults (SPECIFY) 1 2<br>Other things (example: dogs, other animals) 1 2		**1204**
	Other: What adults do you feel threatened by?	________________________________		
	Other: What other things do you feel threatened by?	________________________________		
**1205**	Can you tell me where you feel unsafe or threatened while at school or when going to/from school? **(CHECK ALL THAT APPLY)**	YES NO<br>On the way to or from school 1 2<br>In the school yard 1 2<br>In the classroom 1 2<br>In the school toilet or latrine 1 2<br>Refused 1 2<br>On the playing field 1 2<br>Other (SPECIFY) ____________ 1 2		**1205**
**1206**	Is there a person who you would go to for help if you felt threatened or unsafe at school or when going to/from school?	YES 1<br>NO 2<br>DON'T KNOW 88	 **1208**<br>**1208**	**1206**
**1207**	Who are the people you would go to for help if you felt unsafe or threatened while at school or when going to/from school? **(CHECK ALL THAT APPLY)**	YES NO<br>Mother 1 2<br>Father 1 2<br>Brother 1 2<br>Sister 1 2<br>Male friend 1 2<br>Female friend 1 2<br>Boyfriend 1 2<br>Female teacher 1 2<br>Male teacher 1 2<br>Female mentor or coach 1 2<br>Male mentor or coach 1 2<br>Religious leader 1 2<br>Local admnistration 1 2<br>Other relative 1 2<br>Other nonrelative 1 2		**1207**

1208 Has any **MALE** ever done any of the following things to you, if so, when is the last time it happened: in the last month, in the last 6 months in the last year or more than a year ago? **1208**

	1208a		1208b	1208c			
	Ever Happen		Relation to Male	WHEN HAPPENED			
	YES	NO	Codes Below	IN THE LAST MONTH	IN LAST 6 MONTHS	IN LAST YEAR	MORE THAN 1 YEAR AGO
A. Say or do something to humiliate you in front of others?	1	2		1	2	3	4
B. Threaten to hurt or harm you or someone close to you?	1	2		1	2	3	4
C. Insult you or make you feel bad about yourself?	1	2		1	2	3	4
D. Push you, shake you, or throw something at you	1	2		1	2	3	4
E. Slap you	1	2		1	2	3	4
F. Twist your arm or pull your hair	1	2		1	2	3	4
G. Punch you with his fist or something that could hurt you	1	2		1	2	3	4
H. Kick you, drag you, or beat you up	1	2		1	2	3	4
I. Try to choke you or burn you on purpose	1	2		1	2	3	4
J. Threatened to attack you with a knife or other weapon	1	2		1	2	3	4
K. Attacked you with a weapon	1	2		1	2	3	4
L. Touched you in a sexual way (e.g. kissing, grabbing, or fondling), when you did not want them to	1	2		1	2	3	4
M. Try to have sexual intercourse with you when you did not want	1	2		1	2	3	4
N. Physically forced you to have sexual intercourse even when	1	2		1	2	3	4
O. Forced you to perform sexual acts when you did not want to	1	2		1	2	3	4

RELATIONSHIP CODES FOR 1208b
1 = Husband 6 = Neighbor 11 = Co-worker

		2 = Boyfriend<br>3 = Fiancé<br>4 = Friend, acquaintance<br>5 = Relative	7 = Fellow student<br>8 = Teacher<br>9 = Foreigner/tourist<br>10 = Employer	12 = Parent/Guardian<br>13 = House guard<br>14 = House boy<br>15 = Stranger/unknown person<br>16 = Other ……………..

**1209**	Thinking about what you have experienced among the different things we have been talking about, have you ever tried to seek help to stop them/the/those person(s) from doing this to you again?	YES 1<br>NO 2	**1212**		**1209**

			YES	NO		
**1210**	From whom have you sought help?<br><br>Any one else?<br><br>**[RECORD ALL MENTIONED]**	OWN FAMILY	1	2		**1210**
		HUSBAND/PARTNER'S FAMILY	1	2		
		CURRENT/LAST/LATE HUSBAND/ PARTNER	1	2		
		CURRENT/FORMER BOYFRIEND	1	2		
		FRIEND	1	2		
		NEIGHBOR	1	2		
		RELIGIOUS LEADER	1	2		
		DOCTOR/MEDICAL PERSONNEL	1	2		
		POLICE	1	2		
		LAWYER	1	2		
		SOCIAL SERVICE ORGANIZATION	1	2		
		COMMUNITYLEADER/LOCAL ADMN L	1	2		
		TEACHER	1	2		
		OTHER ______________________ SPECIFY	1	2		

**1211**	Have you ever told any one else about this?	YES 1<br>NO 2		**1211**
**1212**	As far as you know, did your father/guardian ever beat your mother/guardian?	YES 1<br>NO 2<br>DON'T KNOW 88		**1212**

SECTION 13: Decision Making

1301 How often are the following statements true for you? **1301**

	Often	Sometimes	Never/Rarely	Don't know	Refuse
**A.** My parents or guardians ask for my opinion on things	1	2	3	88	99
**B.** My parents or guardians listen when I share my opinion	1	2	3	88	99
**C.** My friends ask my advice when they have a problem	1	2	3	88	99
**D.** If I see something wrong in school or the neighborhood I feel I can tell someone and they will listen	1	2	3	88	99
**E.** I can speak up when I see someone else being hurt	1	2	3	88	99
**F.** I can ask adults for help when I need it	1	2	3	88	99

1302 How often are you able to make each of the following decisions on your own, without an adult? **1302**

	Often	Sometimes	Never/Rarely	Don't know	Refuse
**A.** What clothes to wear when you are not in school/working	1	2	3	88	99
**B.** What to do in your free time	1	2	3	88	99
**C.** What to eat when you are not at home	1	2	3	88	99
**D.** How much education you will get (e.g. Complete secondary school, Go to university)	1	2	3	88	99
**E.** Who you can have as friends	1	2	3	88	99
**F.** Decide when to marry on your own	1	2	3	88	99
**G.** Decide who you will marry on your own	1	2	3	88	99

**1303**	Do you think you will be able to decide on your own when to marry?<br><br>**IF MARRIED, ASK:** Were you able to decide on your own when to marry?	YES 1<br>NO 2<br>DON'T KNOW 88		**1303**
**1304**	Do you think you will be able to decide who you will marry on your own?<br><br>**IF MARRIED, ASK:** Were you able to decide who you would marry? On your own?	YES 1<br>NO 2<br>DON'T KNOW 88		**1304**

END CAPI: If you would like to discuss any of the questions or issues raised in this survey, please feel free to discuss this with the interviewer. The interviewer can provide information to contact a social welfare officer to discuss your concerns, or can contact the social welfare officer for you. Thank you very much for participating in this important study.

SWAHILI

SECTION 1: RESPONDENT'S BACKGROUND

QUESTIONS	RESPONSES	SKIP TO
**[RECORD THE TIME INTERVIEW BEGINS USE 24 HOUR TIME]**	**a.** HOUR [6-20] ☐☐<br>**b.** MINUTES [0-59] ☐☐	
Je, ulizaliwa katika mwezi na mwaka gani?	**a.** MONTH [1-12] ☐☐<br>DON'T KNOW MONTH 88<br>**b.** YEAR [1996-2007] ☐☐☐☐<br>DON'T KNOW YEAR 8888	
Ulikuwa na umri wa miaka mingapi katika adhimisho la mwisho la siku yako ya kuzaliwa?<br>**[COMPARE AND CORRECT 102 AND/OR 103 IF INCONSISTENT]**	AGE IN COMPLETED YEARS [10-20] ☐☐	
Uko darasa la ngapi kwa sasa?	STANDARD [1-8] ☐<br>**IF NOT CLASS 7**	**END INT**
Ulizaliwa katika jimbo gani?	COUNTY CODE [01-49] ☐☐<br>BORN IN SOMALIA 66<br>BORN IN ETHIOPIA 77<br>DON'T KNOW 88<br>BORN IN OTHER COUNTRY ____________ 98<br>SPECIFY	
Mahali unapoishi sasa panaitwaje?<br>Note: Pre-populated list	____________________	
Kwa mfululizo umekua ukiishi [name of current place of residence] kwa muda gani?<br>**[IF LESS THAN ONE YEAR, RECORD '0' YEARS]**	YEARS [0-20] ☐☐<br>ALWAYS (SINCE BIRTH) 77<br>DON'T KNOW 88	**109**
Ukifikiria mahali ulipokua ukiishi kabla ya kuhamia hapa, ilikuwa mji, jiji au kijiji?	TOWN / URBAN AREA 1<br>VILLAGE / RURAL AREA 2	
Katika mwaka mmoja uliopita, umesafiri na kulala mbali na nyumbani kwako mara ngapi tofauti tofauti?	NUMBER OF TRIPS [1-80] ☐☐<br>NONE 0<br>DON'T KNOW 88	**110**
a. Katika muhula wa shule uliopita (kati ya Septemba na Oktoba mwaka jana), ulikuwa mbali na nyumbani kwenu kwa jumla ya siku ngapi?	NUMBER OF DAYS [1-70] ☐☐<br>NONE	110

b. Ni sababu zipi zilizokufanya uwe mbali kwa siku ulizotaja?

[READ ALL OPTIONS]

		YES	NO
**a.**	Kufunga ndoa	1	2
**b.**	Kwa ajili ya shule	1	2
**c.**	Kwa sababu ya matibabu	1	2
**d.**	Singeweza kujimudu kimaisha mahali nilipokuwa	1	2
**e.**	Kuandmana na familia	1	2
**f.**	Matatizo ya kibnafsi nyumbani (mgogoro wa familia, unyanya	1	2
**g.**	Kuenda kazi	1	2
**h**	Kutoroka ndoa za mapema	1	2
**i.**	Kutengana au kutalakiana na mume/mwenzi	1	2
**j**	Kifo cha mzazi au cha wazazi/mlezi	1	2
**k.**	Kutengana au kutalakiana kwa wazazi	1	2
**l.**	Kifo cha mume/mwenzi	1	2
**m.**	Machafuko ya kisiasa	1	2
**n.**	Ukame au umasikini katika maeneo uliotoka	1	2
**o.**	Fedheha (unyanyapaa) kwa sababu ya hali ya ukimwi	1	2
**p.**	Ukosefu wa karo	1	2
**q.**	Aina nyinginezo za fedheha (unyanyapaa) /ubaguzi	1	2
**r**	Nyingine taja		98

QUESTIONS	RESPONSES	SKIP TO
	YES 1	**112**

Je,mama mzazi yungali hai?	NO 2<br>DON'T KNOW 88	 **112**
Je, ulikuwa na umri gani mama yako alipofariki?	AGE IN YEARS [ ]<br>DON'T KNOW 88	
Je, mama yako alisoma mpaka kiwango kipi cha juu zaidi cha elimu?	NO EDUCATION 1<br>NURSERY SCHOOL 2<br>SOME PRIMARY SCHOOL 3<br>COMPLETED PRIMARY SCHOOL 4<br>SOME SECONDARY SCHOOL 5<br>COMPLETED SECONDARY SCHOOL 6<br>PRE-SECONDARY SCHOOL VOCATIONAL/TECHNICAL TRAINING 7<br>POST- SECONDARY SCHOOL VOCATIONAL/TECHNICAL TRAINING 8<br>COLLEGE/UNIVERSITY 9<br>DON'T KNOW 88	
Je, baba yako yungali hai?	YES 1<br>NO 2<br>DON'T KNOW 88	**115**<br><br>**115**
Je, ulikuwa na umri gani baba yako alipofariki?	AGE IN YEARS [ ]<br>DON'T KNOW 88	
Je, baba yako alisoma hadi kiwango kipi cha juu zaidi cha elimu?	NO EDUCATION 1<br>NURSERY SCHOOL 2<br>SOME PRIMARY SCHOOL 3<br>CLASS 8 4<br>SOME SECONDARY SCHOOL 5<br>FORM 4 6<br>PRE-SECONDARY SCHOOL VOCATIONAL/TECHNICAL TRAINING 7<br>POST- SECONDARY SCHOOL VOCATIONAL/TECHNICAL TRAINING 8<br>COLLEGE/UNIVERSITY 9<br>DON'T KNOW 88	

Kabila lako ni gani?

[RECORD ALL MENTIONED]

	YES	NO
EMBU	1	2
KALENJIN	1	2
KAMBA	1	2
KIKUYU	1	2
KISII	1	2
LUHYA	1	2
LUO	1	2
MASAI	1	2
MERU	1	2
MIJIKENDA/SWAHILI	1	2
SOMALI	1	2
BORANA	1	2
TAITA/TAVETA	1	2
NUBIAN	1	2
OTHER ____________ SPECIFY	1	2

Wewe huongea lugha gani nyumbani?

[RECORD ALL MENTIONED]

Note: Update with additional Mijikenda languages

		YES	NO
**a.**	EMBU	1	2
**b.**	KALENJIN	1	2
**c.**	KAMBA	1	2
**d.**	KIKUYU	1	2
**e.**	KISII	1	2
**f.**	LUHYA	1	2
**g.**	LUO	1	2
**h.**	MASAI	1	2
**I.**	MERU	1	2
**j.**	GIRIAMA	1	2
**k.**	SOMALI	1	2
**l.**	KIBORANA	1	2
**m.**	KISWAHILI	1	2
**n.**	ENGLISH	1	2
**o.**	NUBIAN	1	2
**p.**	CHONYI	1	2
**q**	RABAI	1	2
**r**	DURUMA	1	2
**s**	KAUMA	1	2
**t**	DIGO	1	2
**u**	KAMBE	1	2
**v**	JIBANA	1	2
**w**	RIBE	1	2
___**x**	OTHER (SPECIFY) ___________________		

Je, waweza kuongea lugha nyingineyo vizuri kiasi cha kuweza kuendeleza mazungumzo? **[IF YES, ASK:]** Lugha gani/zipi?

		YES	NO
**a.**	EMBU	1	2
**b.**	KALENJIN	1	2

SERIAL #: [________________________]

[RECORD ALL MENTIONED]	c. KAMBA 1 2<br>d. KIKUYU 1 2<br>e. KISII 1 2<br>f. LUHYA 1 2<br>g. LUO 1 2<br>h. MASAI 1 2<br>I. MERU 1 2<br>j. GIRIAMA 1 2<br>k. SOMALI 1 2<br>l. KIBORANA 1 2<br>m. KISWAHILI 1 2<br>n. ENGLISH 1 2<br>o. NUBIAN 1 2<br>P. CHONYI 1 2<br>q RABAI 1 2<br>r. DURUMA 1 2<br>s KAUMA 1 2<br>t DIGO 1 2<br>u KAMBE 1 2<br>v JIBANA 1 2<br>w RIBE 1 2<br>p. OTHER SPECIFY 1 2	
	Dini yako ni gani?	CATHOLIC 1<br>PROTESTANT 2<br>ISLAM 3<br>TRADITIONAL 4<br>NO RELIGION 5<br>OTHER (SPECIFY_____) 96	
	Je, dini ni muhimu kwako kwa kiasi gani?	**READ OUT OPTIONS**<br>MUHIMU SANA 1<br>MUHIMU KIASI 2<br>SIO MUHIMU 3	
	Sasa, ningependa kukuuliza maswali machache kuhusu watu wengine mnaoishi nao. Nitauliza kuhusu elimu na kazi ya kila mtu ambaye unaishi naye. Kando na wewe, ni watu wangapi wengine wanaishi ndani ya nyumba yenu?<br>**EXCLUDING RESPONDENT**	NAMBARI YA WATU [ ]<br>NONE 0	**123**

START BY ASKING WHO THEY LIVE WITH, CONTINUE TO PROBE ABOUT OTHERS WHO LIVE IN THE HOUSEHOLD UNTIL ALL WHO LIVE WITH THE RESPONDENT HAVE BEEN IDENTIFIED. HOUSEHOLD MEMBER IS DEFINED AS SOMEONE WHO REPORTS TO THE SAME HOUSEHOLD HEAD AND EATS FROM THE SAME POT. . ASK SCHOOL/WORK QUESTIONS – 122e & 122f and 122g– ONLY OF PERSONS AGE 5 AND ABOVE

122a<br>Jina	122b<br>Umri/ 8888=DK	122c<br>Jinsia	122d<br>Uhusiano	122e<br>Kiwango cha juu cha elimu alichofikia	122f<br>Je, anasoma? **[If < age 21]**	122g<br>Je, wamefanya kazi kwa ajili ya kulipwa,mwezi ulio pita? **[If parent/guardian]**
		F M			YES NO	YES NO DON'T KNOW
		1 2			1 2	1 2 88
		1 2			1 2	1 2 88
		1 2			1 2	1 2 88
		1 2			1 2	1 2 88
		1 2			1 2	1 2 88
		1 2			1 2	1 2 88
		1 2			1 2	1 2 88

CODES 122e

1	NO EDUCATION
2	NURSERY SCHOOL
3	PRIMARY SCHOOL
4	SECONDARY SCHOOL
5	VOCATIONAL/TECHNICAL TRAINING
6	COLLEGE/ UNIVERSITY
7	NON-FORMAL SCHOOL
#	DON'T KNOW

CODES 122d:

0	Mother	8	Uncle
1	Father	9	Friend
2	Brother	10	Boyfriend/Husband
3	Sister	11	Own Child
4	Grandmother	12	Employer
5	Grandfather	13	Guardian
		14	Niece/Nephew
		15	Other relative
6	Cousin	96	Other(SPECIFY)
88	Aunt		

	Je, KUTA za nje za nyumba unayoishi zimejengwa na vifaa gani?	Matope 1<br>Simiti 2<br>Mabati 3<br>Mbao 4<br>MAWE 5<br>MATOFALI 6<br>Nyasi/Makuti 7<br>NYINGINE (TAJA) _____ 96	
	Je, PAA ya nyumba unayoishi imejengwa na vifaa gani?	Mabati 1<br>Plastiki 2	

		Mbao 3<br>Vigae 4<br>Makuti/Nyasi 5<br>NYINGINE (TAJA) _______ 96	
	Je, SAKAFU ya nyumba unayoishi imejengwa na nini?	Matope 1<br>Simiti 2<br>Mbao / simiti na imefunikwa 3<br>NYINGINE (TAJA) _______ 96	
	Je, maji mnayokunywa nyumbani kwenu yanatoka wapi?	Maji hadi kwa ploti / nyumbani 1<br>Mfereji wa maji ya bure 2<br>Maji ya mfereji yanayouzwa 3<br>Maji ya kisima yanayouzwa 4<br>Kisima kwenye ploti / nyumbai 5<br>Kisima cha kila mtu 6<br>Mto / Kijito / Ziwa la maji 7<br>Maji ya mvua 8<br>Mchuuzi wa maji<br>NYINGINE (TAJA) 96	**130** (1)<br>**130** (Mchuuzi wa maji)
	Maji hupatikana wapi?	Translate options to swahili<br>IN OWN DWELLING 1<br>IN OWN YARD/PLOT 2<br>ELSEWHERE 3	**130** (1)<br>**130** (2)
	Je, inachukuwa muda gani kwenda na kurudi kutoka mahali pa kuteka maji?	**a.** HOURS [0-8] ☐☐☐<br>**b.** MINUTES [0-480] ☐☐☐<br>DON'T KNOW 88	
	Kwa kawaida ni nani huenda kuwachotea maji watu wa nyumbani kwenu?	SELF 1<br>ADULT WOMAN 2<br>ADULT MAN 3<br>OTHER FEMALE CHILD UNDER 15 YEARS OLD 4<br>MALE CHILD UNDER 15 YEARS OLD 5<br>OTHER ____________ 96<br>SPECIFY	
	Je, kwenu nyumbani kuna choo aina gani?	FLUSH TOILET 1<br>VENTILATED PIT LATRINE 2<br>UNVENTILATED PIT LATRINE 3<br>NO FACILITY/BUSH/FIELD 4<br>POUR FLUSH 5<br>OTHER ____________ 98<br>SPECIFY	**133** (4)
	Je mnatumia choo moja na nyumba nyinginezo?	YES 1<br>NO 2	**133** (2)
	Ni nyumba ngapi hutumia choo hiki?	NO. OF HOUSEHOLDS<br>IF LESS THAN 10 ☐<br>10 OR MORE HOUSEHOLDS 95<br>DON'T KNOW 88	
	Je, kuna vyumba vingapi vinavyo tumika kwa kulala katika nyumba yenu?	NUMBER OF ROOMS ☐	
	Kando na wewe, ni watu wangapi wengine, mnalala chumba kimoja?	NUMBER OF MALES<br>NUMBER OF FEMALES ☐	
	Katika mwezi mmoja uliopita, je, kuna siku yoyote ambapo ulikosa chakula kwa sababu hakukuwa na chakula cha kutosha nyumbani?	YES 1<br>NO 2	

READ RESPONSE OPTIONS
Sasa, nataka nikuulize kuhusu vitu ambavyo mnaweza kuwa navyo au msiwenavyo katika nyumba yenu.
Je, watu mnaoishi nao wana vitu hivi?

	NO	YES
A. STIMA	1	2
B. RADIO	1	2
C. RUNINGA/TV	1	2
D. SIMU	1	2
E. TAA YA	1	2
F. KITANDA	1	2
G. BAISKELI	1	2
H. PIKIPIKI	1	2
I. GARI	1	2
J. NYUMBA UNAYOISHI	1	2
K.SHAMBA	1	2
L. MIFUGO NDOGO (I.E. KUKU, MBUZI)	1	2
M. MIFUGO KUBWA (I.E. NGOM'BE, NGURUWE)	1	2
N.FRIJI	1	2
O. MITAMBO YA KULETA PESA	1	2

P. PETE, USHANGA	1 2	

SECTION 2: USE OF SKILLS

Katika maisha yako ya kila siku, wewe husoma mara nyingi kiasi gani kikawaida habari nyinginezo zilizoandikwa? Magazeti,<br><br>**[READ RESPONSE OPTIONS]**	KARIBU KILA SIKU<br>ANGALAU MARA MOJA KWA WIKI<br>ANGALAU MARA MOJA KWA MWEZI<br>CHINI YA MARA MOJA KWA MWEZI<br>KAMWE	1<br>2<br>3<br>4<br>5	
Kwa kawaida katika maisha yako ya kila siku, wewe huandika chochote kiasi gani ?<br><br>**[READ RESPONSE OPTIONS]**	KARIBU KILA SIKU<br>ANGALAU MARA MOJA KWA WIKI<br>ANGALAU MARA MOJA KWA MWEZI<br>CHINI YA MARA MOJA KWA MWEZI<br>KAMWE	1<br>2<br>3<br>4<br>5	
Katika maisha yako ya kila siku, kwa kawaida wewe huisikiliza redio mara nyingi kiasi gani ?<br><br>**[READ RESPONSE OPTIONS]**	KARIBU KILA SIKU<br>ANGALAU MARA MOJA KWA WIKI<br>ANGALAU MARA MOJA KWA MWEZI<br>CHINI YA MARA MOJA KWA MWEZI<br>HAPANA	1<br>2<br>3<br>4<br>5	
Katika maisha yako ya kila siku, kwa kawaida wewe hutazama televisheni mara nyingi kiasi gani ?<br><br>**[READ RESPONSE OPTIONS]**	KARIBU KILA SIKU<br>ANGALAU MARA MOJA KWA WIKI<br>ANGALAU MARA MOJA KWA MWEZI<br>CHINI YA MARA MOJA KWA MWEZI<br>HAPANA	1<br>2<br>3<br>4<br>5	

SECTION 3: RESPONDENT'S SCHOOLING

QUESTIONS	RESPONSES		
Ulianza darasa la kwanza ukiwa na umri gani?	AGE<br>[4-14]<br>DON'T KNOW	[ ][ ]<br><br>88	
Je, umewahi kurudia darasa lolote shuleni?	YES<br>NO	1<br>2	 **304**
Tafadhali niambie ulirudia darasa lipi na mara ngapi?<br><br>**RECORD ALL MENTIONED.**	CLASS 1<br>CLASS 2<br>CLASS 3<br>CLASS 4<br>CLASS 5<br>CLASS 6<br>CLASS 7	Number of imes Repeated<br>[ ][ ]<br>[ ][ ]<br>[ ][ ]<br>[ ][ ]<br>[ ][ ]<br>[ ][ ]<br>[ ][ ]<br>[1-5]<br>NONE 88	
Shule unayoenda inaitwaje?<br><br>Note: Pre-populated list	______________________________		
Je, wewe unaishi nyumbani au kwingineko kwenda shuleni?	LIVE AT HOME<br>LIVE SOMEWHERE ELSE	1<br>2	
Kwa kawaida ni mbinu gani huwa unatumia kufika shuleni?	BY FOOT / WALKING<br>OWN BICYCLE/HOUSEHOLD BICYCLE<br>HIRED BICYCLE TAXI/BODA BODA<br>BUS/MATATU/MINIBUS/OTHER PUBLIC TRANSPORT<br>PRIVATE VEHICLE<br>OTHER (SPECIFY_____)	1<br>2<br>3<br>4<br>5<br>98	**308**<br>**308**<br><br><br>**308**<br>**308**
Kwa kawaida, wewe hulipa pesa ngapi kwa jumla kuenda shule na kurudi?	a. PER DAY<br>PER MONTH<br>PER TERM<br><br>b. KENYA SHILLINGS	1<br>2<br>3<br><br>[ ][ ][ ][ ][ ]	

		DON'T KNOW 88888	
	Kwa kawaida, unatumia mda gani kufika shuleni?	**a.** HOURS [0-5]<br>**b.** MINUTES [300]<br>DON'T KNOW 88	
	Katika muhula wa shule uliopita (kati ya Septemba na Oktoba mwaka jana), uliwahi kosa shule hata siku moja?	YES 1<br>NO 2	 313
	Ni sababu zipi zilizokufanya ukose shule katika muhula uliopita? Ulikosa kwa siku ngapi kwa ajili ya sababu ulioitaja? Kuna sababu nyingine?<br>**DO NOT READ LIST. RECORD ALL MENTIONED.**	Days Missed<br>SELF SICK/ILL<br>NEEDED TO WATCH BROTHERS/SISTERS<br>HOUSEHOLD MEMBER SICK<br>MARKET DAY<br>NEEDED TO DO CHORES AT HOME<br>NEEDED TO RUN ERRANDS FOR PARENTS<br>UNIFORM DIRTY<br>PROBLEMS WITH/AFRAID OF OTHER STUDENTS<br>PROBLEMS WITH/AFRAID OF TEACHER<br>MENSTRUATION<br>WOULD RATHER DO SOMETHING ELSE<br>WENT TO SEE/WAS WITH BOYFRIEND<br>BEREAVEMENT/FUNERAL<br>SCHOOL FEES NOT PAID<br>OTHER (SPECIFY_____)<br>[1-80] 0<br>NONE 88	 **313**
	Je, ni ninni sababu **KUU** iliyokufanya ukose shule katika muhula uliopita?	SELF WAS SICK/ILL 1<br>NEEDED TO WATCH BROTHERS/SISTERS 2<br>HOUSEHOLD MEMBER SICK 3<br>MARKET DAY 4<br>NEEDED TO DO CHORES AT HOME 5<br>WAS SENT SOMEWHERE BY PARENTS/GUARDIAN 6<br>UNIFORM DIRTY 7<br>PROBLEMS WITH/AFRAID OF OTHER STUDENTS 8<br>PROBLEMS WITH/AFRAID OF TEACHER 9<br>MENSTRUATION 10<br>WOULD RATHER DO SOMETHING ELSE 11<br>WENT TO SEE/WAS WITH BOYFRIEND 12<br>BEREAVEMENT/FUNERAL 13<br>SCHOOL FEES NOT PAID 14<br>OTHER (SPECIFY_____) 98	
	**IF MISSED DUE TO MENSTRUATION IN 310 OR 311, ASK 312. OTHERS SKIP TO 313.**		
	Ulipokosa kwenda shule kwa sababu ya hedhi, sababu ilikuwa/zilikuwa ipi/zipi?<br>**[CHECK ALL THAT APPLY]**	**ASK ANYTHING ELSE, DO NOT READ LIST**<br>YES NO<br>a. You had nothing (e.g.sanitary pads or cloth) to manage p 1 2<br>b. You were scared to have accident at school 1 2<br>c. You had pain/cramps/headache 1 2<br>d. You were embarrased 1 2<br>e. You were scared to be teased at school 1 2<br>f. There is no private place to manage period at school 1 2<br>g. Lack of water/place to clean 1 2<br>h. Diarrhea 1 2<br>i. Other (SPECIFY_______________) 1 2	
	Kama nyingine, tafadhali eleza	________________________________	
	Katika wiki iliyopita, vipindi vya shuleni vilikuwa siku ngapi?	DAYS [0-7]<br>**IF 0**	 **316**
	Katika wiki iliyopita, ulienda shuleni siku ngapi?	DAYS<br>[0-7]<br>**IF 313 = 314]**	 **316**
	Katika wiki iliyopita, kwanini ulikosa kwenda shule?<br>**[CHECK ALL THAT APPLY]**	YES NO<br>SELF WAS SICK/ILL 1 2<br>NEEDED TO WATCH BROTHERS/SISTERS 1 2<br>HOUSEHOLD MEMBER SICK 1 2<br>MARKET DAY 1 2<br>NEEDED TO DO CHORES AT HOME 1 2	

	WAS SENT SOMEWHERE BY PARENTS/GUARDIAN	1	2
	UNIFORM DIRTY	1	2
	PROBLEMS WITH/AFRAID OF OTHER STUDENTS	1	2
	PROBLEMS WITH/AFRAID OF TEACHER	1	2
	MENSTRUATION	1	2
	WOULD RATHER DO SOMETHING ELSE	1	2
	WENT TO SEE/WAS WITH BOYFRIEND	1	2
	BEREAVEMENT/FUNERAL	1	2
	OTHER (SPECIFY_____)	1	2
Kama nyingine, tafadhali eleza	______________________________		

School Engagement and Participation Sasa ningependa kukuuliza kuhusu kushiriki kwako na kuwa na haja na shule. Katika maswali haya tungependa utueleze kama unakubaliana au haukubaliani na taarifa zifuatazo.

**[Read options 1 and 2 only]**	**UNAKUBALIANA**	**HAUKUBALIANI**	**NO OPINION /DON'T KNOW**
**a.** Wewe huendi shuleni mara kwa mara. Je wewe…..	1	2	88
**b**. Wewe huwa makini darasani. Je wewe…..	1	2	88
**c**. Ni nadra kwako kumaliza kazi unayopewa darasani. Je wewe…...	1	2	88
**d**. Elimu sio muhimu kwako. Je wewe…….	1	2	88
**e**. Wewe huwa unawaheshimu walimu wako. Je	1	2	88
**f**. Hakuna mtu mzima shuleni ambaye unaweza kuzungumza naye ukiwa na shida. Je wewe……	1	2	88
**g.** Wewe husoma nyumbani hata kama huna mtihani. Je wewe……..	1	2	88
**h.** Mwalimu wako hawezi tambua/kujua ukiwa hujaenda shuleni. Je wewe…..	1	2	88
**i**. Mara mingi huwa unahisi kuwa shule haifurahishi/inaboesha	1	2	88
**Schooling Competence**			
**j**. Una kipaji cha kuelewa masomo yako vyema . Je wewe…..	1	2	88
**k**. Mara nyingi huwa huelewi mafunzo darasani. Je wewe…..	1	2	88
**l.** Ni vigumu kwako kumaliza kazi yako ya shule. Je wewe……..	1	2	88
**m.** Unajihisi sawa kushiriki darasani	1	2	88
**n**. Huwa haujiamini unapojibu maswali darasani	1	2	
**o**.Mara nyingi huwa ni vigumu kwako kuwa makini darasani	1	2	
**p**. Wewe huwa unamaliza kazi yako ya shule kila mara	1	2	88
**q.** Unaamini ya kwamba unao uwezo wa kufanya vyema shul	1	2	88
**Self Esteem**			
**r**. Unaweza kufanya mambo mengi vizuri kama au kuliko wengine wa umri wako. Je wewe…...(-)	1	2	88
**s**. Maoni yako yanathaminiwa nyumbani kwenyu. Je wewe….	1	2	88
**t**. Watu hufikiria kwamba una sifa nyingi nzuri. Je wewe... (+)	1	2	88
**u**. Huwezi kushughulikia au kufaulu kwa mambo mengi unayoyafanya. Je wewe... (-)	1	2	88

Je, mara nyingi mafanikio shuleni huletwa na nini? Talanta, bahati ama kufanya kazi kwa bidii?	TALENT	1
	LUCK	2
	HARDWORK	3
	COMBINATION	4
	NO OPINION/DON'T KNOW	88

Education and Schooling Experience

Nitasoma baadhi ya taarifa kuhusu elimu na masomo. Nataka uniambie ikiwa unakubaliana au hukubalini na taarifa hiyo.

	DISAGREE	2
a.Ni jambo muhimu kwa wasichana kumaliza shule ya upili sawa na lilivyo kwa wavulana kumaliza.	AGREE	1
	DISAGREE	2
b.Familia isipoweza kupeleka watoto wote shuleni, ni bora zaidi kuwapeleka wavulana kuliko wasichana.	AGREE	1
	DISAGREE	2

c.Msichana wa miaka 16 anapaswa aolewe akipata mwenzi afaaye, hata akiwa bado anakwenda shuleni.	AGREE 1<br>DISAGREE 2	

Sasa nitasoma baadhi ya taarifa kuhusu unayoyapitia kwa sasa kimasomo. Nataka uniambie ikiwa taarifa hiyo inakuhusu au inahusu yale unayopitia.

	YES	NO	NA
a. Walimu wako wengi wanawapendelea wavulana zaidi ya wasichana darasani	1	2	8
b. Kunaye na mwalimu wa kike shule ambaye anakuhimiza kufanye vyema shuleni.	1	2	8
c. Baba yako anakubali kwamba unastahili kwenda shule.	1	2	8
d. Mama yako anakubali kwamba unastahili kwenda shule.	1	2	8
e. Wakati mwingine una kazi nyingi za nyumbani hadi unakosa kumaliza kazi ya shule.	1	2	8

Je, ni wakina nani huwa wanapokea maoni mengi mazuri kutoka kwa mwalimu wako wa darasa: wavulana, wasichana, au wanapokea maoni sawa?	NO POSITIVE COMMENTS MADE…………<br>BOYS……………………………………………<br>GIRLS…………………………………………<br>ABOUT THE SAME……………………………<br>DON'T KNOW......................................	0<br>1<br>2<br>3<br>88	
Je, ni wakina nani huwa wanapokea maoni mengi mabaya kutoka kwa mwalimu wako wa darasa: wavulana, wasichana, au wanapokea maoni sawa?	NO NEGATIVE COMMENTS MADE…………<br>BOYS……………………………………………<br>GIRLS……………………………………………<br>ABOUT THE SAME……………………………..<br>DON'T KNOW.......................................	0<br>1<br>2<br>3<br>88	
Je, umewahi kujifunza mafunzo ya kujikimu kimaisha /somo la maisha ya kijamii shuleni mwenu?	Yes<br>No	1<br>2	 **327**

Katika mwaka uliopita (2016), kuna mwalimu wenu yeyote aliyewafunza masomo yoyote kati ya haya darasani?		YES	NO	
	MAWASILIANO	1	2	
	HADHI/KUJIAMI	1	2	
	UJAUZITO	1	2	**If all 'NO':**
	KUBALEHE/JINSI WAVULANA NA WASICHANA WANAVY	1	2	
	HIV/AIDS	1	2	**325**
	MAISHA YA NDOA/MAJUKUMU KATIKA FAMILIA	1	2	
	HEDHI	1	2	
	MPANGO WA UZAZI	1	2	
	KUEPUKANA NA NGONO	1	2	
	AFYA NA USAFI	1	2	
	INGINE (ELEZA)		98	

Je, ni nani aliyefunza masomo hayo?				
	Mwalimu wa kiume	1	1	
	Mwalimu wa kike	1	2	
	Mshauri	1	3	
**[READ LIST, CHECK ALL THAT APPLY]**	INGINE (ELEZA)		98	

Katika mwaka uliopita (2016), kando na walimu wenu, kuna mtu (mtu kutoka nje au shirika lisilo la kiserikali au shirika la kijamii) aliyewafunza masomo yoyote kati ya haya shuleni?		YES	NO	
	MAWASILIANO	1	2	
	HADHI/KUJIAMINI	1	2	
	UJAUZITO	1	2	**If all 'NO':**
	KUBALEGHE/JINSI WAVULANA NA WASICHANA WANAV	1	2	
	HIV/AIDS	1	2	**327**
	MAISHA YA NDOA/MAJUKUMU KATIKA FAMILIA	1	2	
**READ LIST; CHECK ALL THAT APPL**	HEDHI	1	2	
	MPANGO WA UZAZI	1	2	
	KUEPUKANA NA NGONO	1	2	
	AFYA NA USAFI	1	2	
	INGINE (ELEZA)		98	

Je, waliwafunza masomo haya mara ngapi?	KILA WIKI<br>ANGALAU MARA MBILI KWA MWEZI<br>MARA MOJA AU MBILI KWA MUHULA<br>MARA CHACHE	1<br>2<br>4<br>5	
Je, ungependa kusoma hadi kiwango kipi cha juu zaidi?	**DO NOT READ LIST**<br>PRIMARY SCHOOL<br>SECONDARY SCHOOL<br>PRE - SECONDARY VOCATIONAL/TECHNICAL TRAINING<br>POST-SECONDARY VOCATIONAL/TECHNICAL TRAINING<br>COLLEGE/UNIVERSITY	 1<br>2<br>3<br>4<br>5	
Wakati mwingine watu huwa hawawezi kufika mbali kimasomo jinsi wangependa kwasababu nyingi. Je, <u>unatarajia</u> kusoma hadi kiwango kipi?	**DO NOT READ LIST**<br>PRIMARY SCHOOL<br>SECONDARY SCHOOL<br>PRE - SECONDARY VOCATIONAL/TECHNICAL TRAINING<br>POST-SECONDARY VOCATIONAL/TECHNICAL TRAINING<br>COLLEGE/UNIVERSITY	 1<br>2<br>3<br>4<br>5	 <br>**401**<br><br>**401**<br>**401**
Je, nini sababu kuu inayokufanya ufikirie kuwa hutamaliza shule ya u	**DO NOT READ LIST**<br>EXPECT TO GET MARRIED<br>CURRENTLY PREGNANT	 1<br>2	

	CAN'T PAY SCHOOL FEES 3<br>CAN'T AFFORD UNIFORM 4<br>PARENTS NOT SUPPORTIVE 5<br>DOMESTIC RESPONSIBILITIES 6<br>DON'T EXPECT TO PASS KCPE EXAM 7<br>DON'T EXPECT TO GET A PLACE IN SECONDARY 8<br>OTHER (SPECIFY) ____________________ 98	

SECTION 4: WORK, SAVINGS, AND FINANCIAL LITERACY

QUESTIONS	RESPONSES	
Jana, ulitumia muda wa saa ngapi kufanya kazi za nyumbani, kama vile kupika, kusafisha, kufua nguo, kukusanya kuni, kuchota maji?<br><br>**[IF YESTERDAY WAS FRIDAY, SATURDAY OR SUNDAY, ASK ABOUT THURSDAY]**	**a.** HOURS [0-12]<br>**b.** MINUTES [0-720]<br><br>DON'T KNOW 88	
Mbali na kazi zako za nyumbani, umefanya kazi nyingine na ukalipwa kwa pesa taslimu au kwa njia nyingine (ukapewa kitu) katika mwezi mmoja uliopita?	YES<br>NO	405
Kama ujuavyo, wasichana wengine hufanya kazi ili walipwe pesa taslimu au kwa njia nyingine. Wengine huuza vitu, wana biashara ndogo, au hufanya kazi kwenye shamba la familia au katika biashara ya familia.Ili tu kuhakikisha, katika mwezi mmoja uliopita, je, ulifanya kazi au shughuli zozote ukalipwa pesa taslimu au kwa njia nyingine (kwa mfano, kupewa chakula au mali nyingine)?	YES 1<br>NO 2	**405**
Katika mwaka mmoja uliopita, je, umefanya kazi au shughuli zozote mbali na kazi zako za nyumbani na ukalipwa pesa taslimu au kwa njia nyingine?	YES 1<br>NO 2	 **407**
Ulikuwa na umri gani mara ya kwanza ulipofanya kazi au shughuli yoyote ya kulipwa, iwe ulilipwa pesa taslimu au kwa njia nyingine?	AGE [4-20]<br>DON'T KNOW AGE 88	
Je, ni zipi kati ya shughuli au kazi zifuatazo ulizofanya na ukalipwa kwa pesa taslimu au kwa njia nyingine?<br><br>**[READ ALL OPTIONS]**	YES NO<br>**a.** Kazi ya nyumbani 1 2<br>**b.** Kufua nguo/vyombo/nyumba 1 2<br>**c.** Kuchota maji 1 2<br>**d.** Kusuka nywele/urembo 1 2<br>**e.** Kutunza mtoto/uyaya 1 2<br>**f.** kufanya kazi kwa mkahawa 1 2<br>**g.** Kufanya kazi kwa shamba 1 2<br>**h.** Kupakia chakula na vinywaji 1 2<br>**i.** kuuza vitu 1 2<br>**j.** Kufanya kazi ofisini 1 2<br>**k.** Aina nyingine ya biashara za kibinafsi 1 2<br>**k.** Aina nyingine ya kazi za kibarua 1 2<br>**l.** Aina nyingine ya kazi ya kuajiriwa 1 2	
Nataka kukuuliza kuhusu vitu ambavyo mtu anaweza kumiliki. Je, wewe binafsi unamiliki ama unavyo vitu hivi?<br><br>**[READ LIST; IF 'YES' ASK 408]**	**408**<br>Ni nani aliyekupa [ITEM], ni nani aliyekupa pesa za kununua kitu hicho, au ni nani aliyekununulia kitu hicho?<br><br>USE THE FOLLOWING CODES:<br>[DO NOT READ LIST]<br>SELF- WITH MONEY EARNED 1<br>MOTHER 2<br>FATHER 3<br>GUARDIAN 4<br>BOYFRIEND 5<br>HUSBAND 6<br>SUGAR DADDY 7<br>MALE FRIEND 8<br>FEMALE FRIEND 9<br>OTHER RELATIVE 10<br>TEACHER 11<br>OTHER (SPECIFY________) 98	

	YES	NO		CODE
**a.** JOZI LA VIATU	1	2	**a.** A PAIR OF SHOES	
**b.** SARE ZA SHULE	1	2	**b.** SCHOOL UNIFORM	
**c.** SETI MBILI ZA NGUO (OTHER THAN UNIFORM)	1	2	**c.** TWO SETS OF CLOTHES (OTHER THAN UNIFORM)	
**d.** NGUO MBILI ZA NDANI	1	2	**d.** TWO UNDER WEARS	
**e.** MFUKO WA SHULE	1	2	**e.** A SCHOOL BAG	

f.	VITO	1	2	f.	SOME JEWELRY
g.	SIMU YA MKONO/RUNUNU	1	2	g.	A MOBILE TELEPHONE

Sasa nitakuuliza maswali kadhaa kuhusu masuala tofauti-tofauti kuhusiana na pesa zozote ulizolipwa au kupewa, matumizi ya pesa za akiba. Tunajua kwamba wasichana wengine wana pesa za kutumia na za kuweka akiba na wengine hawana. Kwa hiyo hakuna majibu **yaliyo sawa** wala majibu **yasiyo sawa** wewe jibu tu kwa uaminifu kadiri uwezavyo.

Katika mwaka mmoja uliopita, je, ulitumia pesa zozote kwa ajili ya mahitaji yako ya kila siku, mambo mengine tofauti-tofauti au gharama nyinginezo?	YES 1<br>NO 2	 **412**

Katika mwaka mmoja uliyopita, ulipotumia pesa kwa ajili ya mahitaji yako ya kila siku, mambo mengine tofauti-tofauti au kwa gharama nyinginezo, ulipata pesa hizo kutoka wapi?

[RECORD ALL MENTIONED]

		YES	NO
**a.**	MOTHER	1	2
**b.**	FATHER	1	2
**c.**	GUARDIAN YOU LIVE WITH	1	2
**d.**	OTHER RELATIVES	1	2
**e.**	FRIEND	1	2
**f.**	BOYFRIEND	1	2
**g.**	HUSBAND	1	2
**h.**	SUGAR DADDY	1	2
**i.**	STRETCHING MONEY	1	2
**j.**	OWN SAVINGS	1	2
**k.**	SAVINGS GROUP/CHAMA	1	2
**l.**	CASUAL JOBS/CHORES	1	2
**m.**	STEADY JOB	1	2
**n.**	CASH TRANSFERS	1	2
**o.**	SPONSOR	1	2
**p.**	OTHER (SPECIFY________)	1	2

Katika MWEZI mmoja uliopita, ulitumia pesa kununua hiki kitu?

(READ EACH ITEM ALOUD. ASK YES OR NO)

(IF NO ON A, GO TO NEXT ITEM, SKIP B, C)
NOTE: Clarify if it is a personal spending need or for someone else if it is not clear from the type of expenditure)

	**A.**<br>MWEZI mmoja uliopita, ulitumia pesa kununua hii kitu?	**B.**<br>Ulitumia takriban kiasi gani cha pesa kwa hii kitu katika MWEZI mmoja?	**C.**<br>Wewe mwenyewe hujiamulia kununua kitu hiki, huamua pamoja na mtu mwengine au mtu mwengine hukuamulia?
		Ksh	**1 = YOU ALONE**<br>**2 = YOU TOGETHER WITH SOMEONE**<br>**3 = SOMEONE ELSE**
a CHAKULA, CHIPS, PEREMENDE,VINYWAJI/SODA ZAKO	YES 1<br>NO 2		
**b** CHAKULA, CHIPS, PIPI, VINYWAJI/SODA ZA MTU MWENGINE	YES 1<br>NO 2		
**c** NGUO/VIATU ZAKO	YES 1<br>NO 2		
**d** NGUO/VIATU ZA MTU MWENGINE	YES 1<br>NO 2		
**e** TAULO YA HEDHI	YES 1<br>NO 2		
**f** BIDHAA ZA UREMBO/ HUDUMA YA UREMBO	YES 1<br>NO 2		
**g** CREDIT/AIRTIME	YES 1<br>NO 2		

h BURUDANI	YES 1<br>NO 2		
i USAFIRI	YES 1<br>NO 2		
j KODI	YES 1<br>NO 2		

Question	Responses	Skip
Katika miezi sita iliyopita, je umeweka akiba ya pesa au umeweka pesa kando kwa matumizi ya baadaye?	YES 1<br>NO 2	 417
Je unaweza kusema kwamba unajiwekea akiba KILA WIKI, kwa kawaida, wakati mwingine, ama kamwe hujawahi?	ALWAYS 1<br>USUALLY 2<br>SOMETIMES 3<br>NEVER 4	 <br><br>417
Kwa jumla, umeweka akiba ya shilingi ngapi tayari?	KENYA SHILLINGS [ ][ ][ ][ ][ ]<br>DON'T KNOW 88888	

Unaweka akiba ya pesa kwa ajili ya nini?<br><br>**[DO NOT READ LIST]**		YES	NO
	**a.** HALI YA DHARURA	1	2
	**b.** VITU VYA KIBINAFSI	1	2
	**c.** GHARAMA ZA NYUMBANI	1	2
	**d.** KARO YAKO YA SHULE	1	2
	**e.** KARO YA SHULE YA WENGINE	1	2
	**f.** VIFAA VYA SHULE	1	2
	**g.** BIASHARA YAKO MWENYEWE	1	2
	**h.** BIASHARA YA FAMILIA	1	2
	**i.** MATUMIZI YA UJUMLA YA WAKATI UJAO	1	2
	**j.** UKULIMA	1	2
	**k.** GHARAMA ZAKO ZA MATIBABU	1	2
	**l.** GHARAMA ZA MATIBABU ZA WENGINE	1	2
	**m.** USAFIRI/SAFARI	1	2
	**n.** MENGINEO (TAJA________)	1	2

Katika miezi sita iliyopita, umeweka akiba yako pahali pepi kati ya palipotajwa hapa chini?	**READ ALL OPTIONS**	**YES**	**NO**
	BANKI / ACCOUNT YA BENKI	1	2
	AKAUNTI YA KUWEKA AKIBA	1	2
	BENKI YA NYUMBANI/CHINI YA GODORO/KWA SHIMO	1	2
	KISANDUKU/KABATI	1	2
	NA RAFIKI	1	2
	NA MZAZI AU MLEZI	1	2
	CHAMA	1	2
	MWENYE DUKA	1	2
	UEKEZAJI WA KILIMO	1	2
	MPESA	1	2
	INGINE (ELEZA)		96

Question	Responses
Je, wewe unakubaliana au ukubaliani na taarifa ifuatayo: "Ni watu wenye pesa nyingi tu wanaoweza kuweka akiba ya pesa."?	AGREE 1<br>DISAGREE 2

SECTION 5: SOCIAL CAPITAL AND NETWORKS

QUESTIONS	RESPONSES	
	**502**	**503**
Je wewe ni mwanachama wa chama (Kilabu) chochote cha kijamii ulipohuthuria katika mwaka moja uliopita? Kwa mfano wewe ni mwanachama wa …?	Ni lini mara ya mwisho ulipohudhuria chama hicho? ?	Je, kilikuwa chama cha wasichana peke yake au cha mchanganyiko wa wasichana

[READ LIST; IF 'YES' ASK 502 and 503]	1 = Within last 7 days<br>2 = Within the last month<br>3 = Within the last one year	na wavulana?

		YES	NO		LAST 7 DAYS	LAST MONTH	IN THE LAST ONE YEAR		Girls Only	Mixed
**a.**	Chama cha jinsia	1	2	**a.**	1	2	3	**a.**	1	2
**b.**	Haki za watoto/Haki za kibinadamu	1	2	**b.**	1	2	3	**b.**	1	2
**c.**	Chama cha mwongozo na kutoa mashauri ya UKIMWI/stadi ya afya ya maisha	1	2	**c.**	1	2	3	**c.**	1	2
**d.**	Wasichana viongozi/skauti	1	2	**d.**	1	2	3	**d.**	1	2
**e.**	Chama cha michezo	1	2	**e.**	1	2	3	**e.**	1	2
**f.**	Kikundi cha kuigiza au kucheza densi	1	2	**f.**	1	2	3	**f.**	1	2
**g.**	Kikundi cha kanisa/kikundi cha Kiislam	1<br>1	2	**g.**	1	2	3	**g.**	1	2
**h.**	Kingine chochote ______________	1	2	**h.**	1	2	3	**h.**	1	2

[FIRST ASK ALL QUESTIONS FOR MALES AND RECORD ANSWERS IN COLUMN A; THEN RETURN TO ASK ALL QUESTIONS FOR FEMALES AND RECORD ANSWERS IN COLUMN B]

Sasa nataka nikuulize kushusu rafiki zako wa karibu. Namaanisha, rafiki mwema ni yule unayeweza kumfichulia maneno yako binafsi na maneno muhimu.

**[FRIENDS: 0-80; RECORD 80 IF MORE THAN 80; RECORD 88 IF DON'T KNOW]**	**A. MALES**	**B. FEMALES**
Una rafiki wangapi wa karibu walio (WAUME/WAKIKE)?	[ ][ ]<br>**[IF = 0 → "FEMALES"]**	[ ][ ]<br>**[IF =A= 0 & B=0 → 507]**
Ni wangapi kati ya hao rafiki (WAUME/WAKIKE) wanaoenda shuleni kwa sasa?	[ ][ ]<br>**[IF = 0 → 'FEMALES']**	[ ][ ]<br>**[IF = A=0 & B=0 → 507]**
Ni wangapi kati ya hao rafiki (WAUME/WAKIKE) wanaoenda shuleni mwako?	[ ][ ]	[ ][ ]

SOCIAL ASSETS AND SAFETY NETS

Nitakusomea taarifa nyinginezo zaidi kisha nikuulize kama unakubaliana au haukubaliani

Nina rafiki wengi katika mtaa wangu/jamii yangu?	AGREE<br>DISAGREE	1<br>2
Nina hisi nikiwa salama nikitembea katika mtaa wangu/ jamii yangu mchana.	AGREE<br>DISAGREE	1<br>2
Nina hisi nikiwa salama nikitembea katika mtaa wangu/ jamii yangu giza inapoingia	AGREE<br>DISAGREE	1<br>2
Watu katika mtaa wangu/jamii yangu huaminiana.	AGREE<br>DISAGREE	1<br>2
Nyakati nyingine, katika maisha yangu ya kila siku, mimi huogopa nitabakwa.	AGREE<br>DISAGREE	1<br>2
Nimeguswa isivyofaa na mvulana au mwanamume katika miezi sita iliyopita	AGREE<br>DISAGREE	1<br>2
Nimeibiwa katika miezi sita iliyopita katika mtaa wangu/ jamii yangu	AGREE<br>DISAGREE	1<br>2
Nimeibiwa katika miezi sita iliyopita nje ya mtaa wangu/ jamii yangu	AGREE<br>DISAGREE	1<br>2

Katika mtaa wangu/jamii yanga, vijana au wanaume hunichezea-chezea niendeleapo na shughuli zangu za kila siku	AGREE 1<br>DISAGREE 2	
Ninaye rafiki mzuri katika jamii yangu ambaye naweza kumwendea ili nipate usaidizi nikiwa na tatizo zito.	AGREE 1<br>DISAGREE 2	
Kuna mwanamke fulani katika jamii yangu, asiye mzazi wala mwalimu wangu, ambaye naweza kumwendea kwa msaada nikiwa na tatizo zito.	AGREE<br>DISAGREE	
Ninaye rafiki mzuri ninayekutana naye mara kwa mara , ambaye naweza kuzungumza naye kuhusu shida zangu na furaha zangu, na kumwuliza maswali.	AGREE<br>DISAGREE	
Kuna mwanamke fulani katika maisha yangu, ninayekutana naye kwa kawaida, ambaye si mamangu wala mwalimu wangu, ambaye naweza kuzungumza naye kuhusu shida zangu na furaha zangu, na kumwuliza maswali.	AGREE 1<br>DISAGREE 2	

Q520A Je, ungeweza kutegemea watu wafuatao ikiwa ungekuwa na uhitaji wa haraka wa pesa?

[READ THE LIST. IF NO ON Q518A, GO TO NEXT ITEM, SKIP Q518B, Q518C]

Q520B Sex

Q520C Wanaishi wapi? (CODE)

CODES
1 Resides in my household
2 Resides in this community
3 Resides in neighboring community
4 Resides elsewhere/ upcountry (rural area)

		YES	NO	Male Only	Female Only	Both	Male CODES	Female CODES
A	Mzazi/Mlezi	1	2	1	2	3		
B	Ndugu/Dada	1	2	1	2	3		
C	Mtu wa Ukoo	1	2	1	2	3		
D	Rafiki wa karibu	1	2	1	2	3		
E	Jirani	1	2	1	2	3		
F	Rafiki mvulana/bwana	1	2	1	2	3		
G	Mshauri	1	2	1	2	3		
H	Mwalimu	1	2	1	2	3		

Q521A Je, unaweza mwendea yeyote kati ya watu hawa ukiwa na swali kuhusu afya yako au mwili wako?

[READ THE LIST. IF NO ON Q519A, GO TO NEXT ITEM, SKIP Q519B, Q519C]

Q521B Sex

Q521C Residence/location (CODE)

1 Resides in my household
2 Resides in this community
3 Resides in neighboring community
4 Resides elsewhere/ upcountry (rural area)

		YES	NO	Male Only	Female Only	Both	Male CODES	Female CODES
A	Mzazi/ Mlezi			**1**	**2**	**3**	**Add boxes**	
B	Ndugu/Dada	1	2	1	2	3		
C	Mtu wa ukoo	1	2	1	2	3		
D	Rafiki wa karibu	1	2	1	2	3		
E	Jirani	1	2	1	2	3		
F	Rafiki mvulana/bwana	1	2	1	2	3		
G	Mshauri	1	2	1	2	3		
H	Mwalimu	1	2	1	2	3		

Kwa kila moja ya mahali panapofuata, tafadhali niambie kama hauruhusiwi kwa kawaida kwenda hapo ukiwa peke yako, wakati tu ambapo umeandamana na mtu mwingine, ama hurusiwe kwenda hapo hata kidogo.

Kliniki ya matibabu ya kwenu	ON MY OWN 1<br>IF SOMEONE ACCOMPANIES ME 2<br>NOT AT ALL 3<br>NEVER GO THERE 9	
Nyumba za rafiki katika mtaa wako	ON MY OWN 1<br>IF SOMEONE ACCOMPANIES ME 2	

		NOT AT ALL 3<br>NEVER GO THERE 9	
	Kikundi cha vijana/Kikundi cha wasichana	ON MY OWN 1<br>IF SOMEONE ACCOMPANIES ME 2<br>NOT AT ALL 3<br>NEVER GO THERE 9	
	Mambo mengi tofauti tofauti yanaweza kuzuia wasichana wasipate ushauri wa afya au matibabu. Unapokuwa mgonjwa na wataka kupata ushauri wa kitiba au matibabu, je, yafuatayo ni tatizo kubwa au la?<br><br>**[READ THE LIST]**<br>**a.** Kupata ruhusa kwenda?<br>**b.** Kupata pesa zinazohitajika kwa ajili ya matibabu?<br>**c.** Umbali wa kituo cha afya?<br>**d.** Kuhitaji kuchukua usafiri/uchukuzi?<br>**e.** Kutotaka kwenda peke yako?<br>**f.** Kuwa na wasiwasi kwamba huenda kusiwe na muhudumu wa afya wa kike?<br>**g.** Kuwa na wasiwasi kwamba huenda kusiwe na muhudumu wa afya yeyote?<br>**h.** kuwa na wasiwasi kwamba huenda kusiwe na madawa?<br>**i.** Kuwa na wasiwasi kwamba huenda kusiwe na faragha/usiri<br>**j.** Kuogopa kufikiriwa vibaya na mhudumu wa afya<br>**k.** Kuona aibu kuzungumzia swala hilo la afya	**BIG PROBLEM**<br>YES NO<br>**a.** PERMISSION 1 2<br>**b.** MONEY 1 2<br>**c.** DISTANCE 1 2<br>**d.** TRANSPORT 1 2<br>**e.** GO ALONE 1 2<br>**f.** NO FEMALE PROVIDER 1 2<br>**g.** NO PROVIDER 1 2<br>**h.** NO DRUGS 1 2<br>**i.** LACK OF PRIVACY/CONFIDENTIALITY 1 2<br>**J.** JUDGEMENT BY PROVIDER 1 2<br>**k.** EMBARRASSMENT 1 2	
	Katika miezi sita iliyopita, je ulienda kwenye kituo cha afya kupata aina yoyote ya huduma za afya?	YES 1<br>NO 2	 **529**
	Ulizuru gani kati ya vituo vya afya vifuatavyo katika miezi sita iliyopita?	KILINIKI YA KIBNAFSI 1<br>HOSPITALI YA UMMA (SEREKALI) 2<br>KITUO CHA AFYA AU KILINIKI YA UMMA (SEREKALI) 3<br>ZAHANATI 4<br>DUKA LA DAWA 5<br>HUDUMA TAMBA (ZA KUZUNGUKA) 6<br>VITUO VIZIVYO VYA SEREKALI 7<br>INGINE (TAJA) ………. ______________________________ 98	
	Tafadhali niambie ni huduma zipi za afya ulizopokea ulipotembelea kituo cha afya?<br><br>**[RECORD ALL MENTIONED]**	**SERVICE CODES**<br>a. GENERAL HEALTH/ SICKNESS 1<br>b. FAMILY PLANNING 2<br>c. PREGNANCY TEST 3<br>d. ANTENATAL CARE 4<br>e. HIV TESTING 5<br>f. HIV CARE/TREATMENT 6<br>g. STI TESTING (NON-HIV) 7<br>h. STI CARE/TREATMENT (NON-HIV) 8<br>i. CHILD HEALTH/SICKNESS 9<br>j. DELIVERY 10<br>k. POSTNATAL CARE 11<br>l. URINARY TRACT INFECTIONS (UTIs) CARE/TREATMENT 12<br>m. OTHER REPRODUCTIVE HEALTH SERVICES 13<br>n. PHARMACY/VITAMINS/MEDICINE 14<br>o. OTHER (SPECIFY) _______________________________ 98	
	**Ningependa ufikirie siku yako ya wiki ya karibuni zaidi, kutoka Jumatatu hadi Ijumaa, ambayo haikuwa sikukuu ya umma au ya shule na ujaribu kukumbuka shughuli tofauti ulizofanya siku hiyo. Niambie kuhusu hizo shughuli tofauti ulizofanya na muda uliochukua kuzifanya.**		
	[RECORD HOW MANY HOURS SHE SPENT ON VARIOUS ACTIVITIES DURING THE LAST COMPLETE WEEKDAY – NOT SATURDAY OR SUNDAY OR HOLIDAY .]	**ACTIVITY** **NUMBER OF MINUTES**<br>**a**. Shuleni [ ]<br>**b**. Kazi ya bila malipo nyumbani (k.m.. Kupika, kusafisha, kufua nguo) [ ]<br>**c.** Kazi ya bila malipo nje ya nyumbani (k.m. kwenda sokoni, kuchota maji) [ ]<br>**d.** Kazi ya malipo kazini/kazi ya malipo kwa ajili ya wengine/ katika biashara ya kibinafsi [ ]	

**e.** Wakati wa mapumziko (kuongea na rafiki, kunywa chai/kahawa na rafiki, kusoma, kutazam televisheni)	[ ]
**f.** Kanisa/Mskiti/Kuomba/duksi/madrassa	[ ]
**g.** Kusoma/kufanya kazi ya ziada ya shule	[ ]
h. Kusafiri kwenda na kutoka shuleni	[ ]
i. Kulala	[ ]
**k. TOTAL MINUTES [CHECK NOT MORE THAN 1440]**	[ ]

SECTION 6: SELF-EFFICACY, LOCUS OF CONTROL, AND GENDER ATTITUDES

QUESTIONS | **RESPONSES**

Generalized Self Efficacy

Sasa nitakusomea baadhi ya taarifa, tafadhali niambie kama unakubaliana nazo au haukubaliani.

	AGREE	DISAGREE	DON'T KNOW
**a**. Kila mara unaweza kusuluhisha matatizo magumu ukijaribu sana.	1	2	88
**b**. Mtu akikupinga, bado unaweza kupata njia za kufanya utakavyo.	1	2	88
**c**. Ni rahisi kwako kukazia fikira malengo yangu na kutimiza miradi yangu.	1	2	88
**d. Una** uhakika kwamba ungeweza kushughulikia matukio yasiyotarajiwa vizuri sana.	1	2	88
**e.** Kwa sababu ya msaada ambao unaweza kupata, unajua kwamba unaweza kukabiliana na hali zisizotarajiwa.	1	2	88
**f**. Unaweza kutatua matatizo yaliyo mengi ukijitahidi inavyohitajiwa.	1	2	88
**g**. Unaweza kubaki ukiwa mtulivu ukabiliwapo na magumu kwa sababu unaweza kutegemea uwezo wako mwenyewe.	1	2	88
**h.** Unapokabili tatizo, kwa kawaida unaweza kupata suluhisho zaidi ya moja.	1	2	88
**i. U**kiwa na tatizo, kwa kawaida unaweza kufikiria suluhisho.	1	2	88
**j.** Kwa kawaida unaweza kushughulikia hali yoyote inayokupata.	1	2	88

Self-Efficacy (India Transition Study)

QUESTIONS	RESPONSES	
Je, wewe hutoa maoni yako kwa wakubwa wako katika familia yako? Unaweza kusema ni mara kwa mara, wakati mwingine au hujawahi?	OFTEN<br>SOMETIMES<br>NEVER	1<br>2<br>3
Usipokubaliana na mtu, je kwa kawaida wewe humwambia huyo mtu kwamba hukubaliani naye? Unaweza kusema kwamba wewe humkabili, wakati mwingine unajibu ama wewe hunyamaza kila mara?	ALWAYS CONFRONT<br>SOMETIMES GIVE RESPONSE<br>ALWAYS,STAY QUIET	1<br>2<br>3
Ikiwa mvulama au mwanaume angejaribu kukushika kwenye sehemu zako za siri bila ya wewe kutaka. Je, ungeweza	YES<br>NO	1<br>2
Je, unajiamini kuweza kuzungumza mbele ya kikundi cha watu wa rika lako? Unaweza kusema ni kila mara, wakati mwingine au hujiamini kamwe?	ALWAYS<br>SOMETIMES<br>NEVER	1<br>2<br>3

Kwa taarifa zifuatazo tafadhali nieleze kama unakubaliana sana, unakubaliana kidogo, hukubaliani kidogo, hukubaliani sana,

**Locus of Control**	**UNAKUBALI ANA SANA**	**UNAKUBALIA NA KIDOGO**	**HAUKUBALIA NI KIDOGO**	**HAKUBALIAN I SANA**	**SIJUI**
**a** Kwa kiwango kikubwa mambo muhimu yanayotendeka maishani mwangu yanatendeka kibahati.	1	2	3	4	88
**b** Kufikia sasa, mafanikio maishani mwangu yamekuwa kwasababu ya uwezo wangu na bidii.	1	2	3	4	88
**c.** Kufaulu au kutofaulu kwangu katika mtihani wa KCPE kutategemea vitendo vyangu na kile nifanyacho	1	2	3	4	88
**d.** Ninapofaulu huwa mara mingi ni kwasababu ya bahati na nisipofaulu huwa mara mingi ni kwasababu ya nguvu ambazo watu wengine wanazo	1	2	3	4	88
**e.** Ikiwa nitafaulu kuafikia malengo yangu maishani, itakuwa ni kwasababu ya vile nilivyojitayarisha vyema.	1	2	3	4	88
**f.** Shida zangu nyingi zitatoweka nikizipuuza	1	2	3	4	88

GENDER NORMS (GEAS)

Kwa taarifa zifuatazo tafadhali nieleze kama unakubaliana sana, unakubaliana kidogo, hukubaliani kidogo, hukubaliani sana,

**Girls Freedom Versus Lack of Independence**	**UNAKUBALI ANA SANA**	**UNAKUBALIA NA KIDOGO**	**HAUKUBALIA NI KIDOGO**	**HAUKUBALIA NI SANA**	**SIJUI**
**a.** Wasichana wanafaa kujitegemea kama wavulana	1	2	3	4	88
**b.** Wasichana hawafai kutoka na marafiki zao isipokuwa tu kama kuna mtu mzima.	1	2	3	4	88
**c.** Wasichana huwa wanahisi kwamba uhuru wao ni mdogo ukilinganisha na wa wavulana kuhusiana na kile wanachoweza kufanya na pahali wanapoweza kwenda	1	2	3	4	88
**d.** Wasichana wanafaa kuwa na nafasi sawa na wavulana	1	2	3	4	88
**e.** Wasichana wanafaa kuwa na uhuru wa kutembea kama wavulana	1	2	3	4	88
**Girls Responsibilities**					
**f.** Ni muhimu kwa wasichana kufanya vyema shuleni jinsi ilivyo muhimu kwa wavulana	1	2	3	4	88
**g.** Wavulana na wasichana wanafaa kujukumika kwa usawa katika kazi za nyumbani	1	2	3	4	88
**Deference/Proper/Composed**					
**h.** Wasichana wanafaa kujiwekea fikira zao na wasiseme wanachofikiria kwa umma	1	2	3	4	88
**i.** Wasichana wanatarajiwa kuwa wanyenyekevu	1	2	3	4	88
**j.** Wasichana hawafai kutoa madai mengi sana kwa wengine	1	2	3	4	88
**k.** Msichana anafaa kusema anachofikiria hata kama kitaumiza hisia za mtu	1	2	3	4	88
**l.** Wasichana wanaobishana na rafiki zao hadharani hawana tabia za kike.	1	2	3	4	88
**Girls are Responsible for Arousing Boys**					
**m** Wasichana wanafaa kujifunika, la sivyo watavutia kimapenzi isivyohitajika	1	2	3	4	88
**n.** Wasichana wanafaa kuwa waangalifu kuhusu jinsi wanavyo onekana ili wasionekane ni kama wanajaribu kuwatongoza	1	2	3	4	88
**o.** Ni makosa yake msichana wavulana wakimtongoza	1	2	3	4	88
**p.** Wasichana huvaa nguo fupi ili kuwavutia wavulana	1	2	3	4	88
**q.** Wasichana wanafaa kuwa huru kuvaa wanayotaka	1	2	3	4	88

SECTION 7. MARRIAGE & SEXUAL BEHAVIOR

QUESTIONS	RESPONSES	
Je, umewahi kuolewa?	NDIO 1<br>LA 2	**703**
Je, unatarajia kuolewa ukiwa na miaka mingapi?	UMRI [10-50] [ ][ ]<br>SIJUI 88<br>SITARAJII KUOLEWA 98<br>**ALL**	**705**
Je, uliolewa ukiwa na miaka mingapi?	UMRI [10-20] [ ][ ]<br>SIJUI 88	
Je, kwa sasa umeolewa, umetengana, umetalikiana au umefiwa na mwenzi?	NIMEOLEWA/NAISHI NA MWENZI 1<br>NIMETENGANA 2<br>NIMETALAKIANA 3<br>NIMEFIWA NA MWENZI 4	
Je, umewahi kuishi pamoja na mpenzi wa kiume?	NDIO 1<br>LA 2	**707**
Je,ulikuwa na miaka mingapi ulipoanza kuishi na mpenzi wako?	UMRI [10-20] [ ][ ]<br>SIJUI 88	
Ulikuwa na umri wa miaka mingapi ulipofanya ngono kwa mara ya kwanza?	SIJAWAHI KUFANYA NGONO 0<br>CHINI YA MIAKA 7 1<br>UMRI [7-20] [ ][ ]<br>SIJUI 88	**801**

	Ulikuwa na uhusiano gani na mtu wa kwanza uliyefanya ngono naye?<br><br>**[DO NOT READ OPTIONS]**	MUME 1<br>MPENZI 2<br>"MZEE MPENZI" (BUZI) 3<br>MTU UNAYEMJUA 4<br>JAMAA YAKO 5<br>MWALIMU 6<br>MTU USIYEMJUA 7<br>MTU MWINGINE 8	
	Je, mtu wa kwanza uliyefanya ngono naye alikuwa na umri mkubwa kuliko wewe, alikuwa mchanga kuliko wewe au alikuwa na umri karibu sawa na wewe?	UMRI MKUBWA KULIKO WEWE 1<br>MCHANGA KULIKO WEWE 2<br>UMRI KARIBU SAWA NA WEWE 3<br>SIJUI / SIKUMBUKI 88	 **711**<br>**711**<br>**711**
	Je, alikuwa na miaka ngapi zaidi yako?	MKUBWA KWA MIAKA MINGAPI [ ][ ]<br>SIJUI 88	
	Mara ya kwanza ulipofanya ngono ulitumia kondomu/mpira?	NDIO 1<br>LA 2<br>SIJUI / SIKUMBUKI 88	
	Mara ya kwanza ulipofanya ngono, je, ulitaka kufanya ngono, au haukutaka kufanya ngono, ama haukuwa na uhakika kama ulitaka kufanya ngono?	ULITAKA KUFANYA NGONO 1<br>HUKUTAKA KUFANYA NGONO 2<br>HAUKUWA NA HAKIKA 3	
	Sasa ningependa kukuuliza baadhi ya maswali kuhusu utendaji wako wa ngono wa hivi karibuni. Ni lini mara ya mwisho ulipofanya ngono?	LEO/WIKI MOJA HAIJAPITA 1<br>WIKI MOJA AU ZAIDI ILIYOPITA 2<br>MWEZI MMOJA ULIOPITA AU ZAIDI 3<br>MWAKA MMOJA ULIOPITA AU ZAIDI 4	 **716**<br>**716**<br>**716**
	Katika wiki moja iliyopita, ulifanya ngono mara ngapi?	MARA NGAPI ULIFANYA NGONO [1-35] [ ][ ]<br>SIJUI 88	
	Nyakati ulipofanya ngono katika wiki iliyopita, ulitumia kondomu mara ngapi?<br><br>**[Consistency check: Maximum = previous question]**	MARA NGAPI ULITUMIA KONDOMU [1-35] [ ][ ]<br>HUKUTUMIA KONDOMU 77<br>88<br>SIJUI	
	Ni nini uhusiano wako na mtu wa mwisho uliyefanya ngono naye?	MUME 1<br>MPENZI 2<br>"MZEE MPENZI" (BUZI) 3<br>MTU UNAYEMJUA 4<br>JAMAA 5<br>MWALIMU 6<br>MTU USIYEMJUA 7<br>MTU MWINGINE 8	
	Je, yule mtu wa mwisho uliyefanya ngono naye alikuwa na umri mkubwa kuliko wewe, alikuwa mchanga kuliko wewe, au alikuwa na umri karibu sawa na wewe?	UMRI MKUBWA KULIKO WEWE 1<br>MCHANGA KULIKO WEWE 2<br>UMRI KARIBU SAWA NA WEWE 3<br>SIJUI / SIKUMBUKI 88	 **720**<br>**720**<br>**720**
	Je, alikuwa na miaka ngapi zaidi yako?	MKUBWA KWA MIAKA MINGAPI [ ][ ]<br>SIJUI 88	
	Mara ya **mwisho** ulipofanya ngono, je, ulitaka kufanya ngono, au haukutaka kufanya ngono, ama haukuwa na uhakika kama ulitaka kufanya ngono?	ULITAKA KUFANYA NGONO 1<br>HUKUTAKA KUFANYA NGONO 2<br>HAUKUWA NA HAKIKA 3	
	Je, mara ya mwisho ulipofanya ngono, kondomu ilitumiwa?	NDIO 1<br>LA 2<br>SIJUI / SIKUMBUKI 88	
	Kwa wastani, ulitumia kondomu mara nyingi kadri gani katika mwaka mmoja uliopita ulipofanya ngono na mtu huyo? Unaweza kusema ni kila mara, wakati mwingine ama hukutumia kamwe?	KILA MARA 1<br>WAKATI MWINGINE 2<br>HUKUTUMIA KAMWE 3	
	Kama ungependa kupata njia ya kupanga uzazi, je waona kama ingekuwa rahisi, vigumu au isingewezekana kwenda mahali ambapo waweza kupata njia ya kupanga uzazi?	RAHISI 1<br>VIGUMU 2<br>ISINGEWEZEKANA 3<br>SIJUI 88	
	**[SKIP Q728 IF Q713 IS A YEAR OR MORE AGO]**		
	Kwa jumla, umefanya ngono na watu wangapi tofauti katika miezi [illegible]	IDADI YA WAPENZI KATIKA MIEZI 6 ILIYOPITA [1-35] [ ][ ]	

Kwa jumla, umefanya ngono na watu wangapi tofauti katika miezi iliyopita ?	SIJUI 88	
Sasa nitasoma orodha ya watu wa aina tofauti na ninataka uniambie kama umewahi kufanya ngono na mtu wa aina hiyo.<br><br>**[READ THE LIST]**	YES NO<br>A. MTALII AU MGENI 1 2<br>B. MWAJIRI WAKO 1 2<br>C. MTU ALIIYE NA UMRI WA MIAKA KUMI AU ZAIDI KULIKO \ 1 2<br>D. MWALIMU WAKO 1 2<br>E. MTU ALIYEKULIPA UFANYE NGONO NAYE 1 2<br>F. MTU ALIYEFUNGA NDOA NA MTU MWINGINE 1 2<br>G. SI YEYOTE KATI YA WALIOTAJWA JUU 1 2	
Kwa maswali yafuatayo, tafadhali fikiria mambo uliyoyapitia kisha uniambie kama unakubaliana au hukubiliani na yafuatayo kujihusu.		
Hujaweza sikuzote kutumia kondomu wakati ambapo umetaka kuitumia.	UNAKUBALIANA 1<br>HAUKUBALIAN 2	
Umefanya ngono na mtu ili akulipie kodi ya nyumba.	UNAKUBALIANA 1<br>HAUKUBALIAN 2	
Umefanya ngono wakati ambapo hujataka kufanya ngono.	UNAKUBALIANA 1<br>HAUKUBALIAN 2	
Katika miezi sita iliyopita, je, umefanya ngono na mtu kwa sababu ulihitaji au ulidhani utapata yafuatayo?	YES NO<br>**a.** Chakula 1 2<br>**b.** Makao/mahali pa kukaa 1 2<br>**c.** Karo ya shule 1 2<br>**d.** Taulo za hedhi 1 2<br>**e.** Pesa za kitu kingine 1 2<br>**f.** Chochote kingine 1 2	 <br><br><br>**SPECIFY**<br>**SPECIFY**
Pesa za kitu kingine, eleza____		
Chochote kingine, eleza____		

SECTION 8: MENSTRUATION

QUESTIONS	RESPONSES	
Je, umeshawahi kusikia kuhusu hedhi ama damu ya mwezi, ambayo pia inajulikana kama 'kupata wageni', 'kuvuja/kuvudza', 'kupata hedhi'?	YES 1<br>NO 2	 **901**
Je, ulipata habari kuhusu hedhi **MARA YA KWANZA** kutoka kwa nani ama wapi?<br><br>**MORE THAN ONE ANSWER POSSIBLE. CIRCLE ALL THAT APPLY**	**YES NO**<br>MOTHER 1 2<br>GRANDMOTHER 1 2<br>SISTER 1 2<br>AUNT 1 2<br>FRIEND 1 2<br>TEACHER 1 2<br>OTHER FAMILY MEMBER 1 2<br>NGO/CBO 1 2<br>HEALTH WORKER/CLINICIAN/DOCTOR 1 2<br>MEDIA 1 2<br>OTHER (SPECIFY________) 1 2	
If you said other, please specify	____	
Je, umeshawahi kupata hedhi/damu ya mwezi?	YES 1<br>NO 2	 **901**
Je, ulijua kuhusu hedhi kabla itokee kwako?	YES 1<br>NO 2<br>NO ANSWER 96	
Ulianza kupata hedhi ulipokuwa na umri wa miaka mingapi?	AGE<br>[4-20]<br>DON'T KNOW 88	
Je, ulipopata hedhi kwa mara ya kwanza, ulimwambia mtu yo yote?	YES 1<br>NO 2	 **808**
Je, ulimwambia nani?	**YES NO**<br>MOTHER 1 2<br>GRANDMOTHER 1 2	

MORE THAN ONE ANSWER POSSIBLE. CIRCLE ALL THAT APPLY	SISTER 1 2<br>AUNT 1 2<br>FRIEND 1 2<br>TEACHER 1 2<br>HEALTH FPROFESSIONAL 1 2<br>OTHER (SPECIFY) 98	

Question	Response	AT SCHOOL	AT HOME	Skip
Ni njia ipi kuu wewe hutumia ili kumudu, hedhi yako kwa sasa shuleni na nyumbani?	Sanitary pads (disposable)	1	1	
	Tampon	2	2	
	Tissue paper/ other paper	3	3	
	Cotton wool	4	4	
	Old pieces of cloth	5	5	
	New peieces of cloth	6	6	
	Sanitary pads (reusable)	7	7	
	Matress pieces	8	8	
	Other (specify)	98	98	

Question	Response	YES	NO	Skip
Je , kwa kawaida wewe hupata wapi vitu ulivyovitaja? **[CHECK ALL THAT APPLY]**	I BUY THEM			
	FROM MY HOME	1	2	**811**
	GIVEN BY MOTHER	1	2	**811**
	GIVEN BY OTHER RELATIVE	1	2	**811**
	FROM SCHOOL	1	2	**811**
	FROM FRIENDS	1	2	**811**
	GIVEN BY AN NGO/CBO	1	2	**811**
	BOYFRIEND	1	2	**811**
	OTHER		98	**811**
Kama nyingine, (TAJA)	____________________			

Question	Response	YES	NO	Skip
Je, wewe hupata wapi pesa? **[CHECK ALL THAT APPLY]**	From parents	1	2	
	Job (specify job)	1	2	
	Own savings	1	2	
	Boyfriend	1	2	
	Other family member	1	2	
	Other		98	
Kama nyingine, (TAJA)	____________________			

Question	Response	YES	NO	Skip
Mbali na njia kuu uliotaja, wewe hutumia nini kingine kuikabili hedhi yako? **[CHECK ALL THAT APPLY]**	Sanitary pads (disposable)			
	Tampon	1	2	**IF NO ON ALL, SKIP 813**
	Tissue paper/ other paper	1	2	
	Cotton wool	1	2	
	Old pieces of cloth	1	2	
	New peieces of cloth	1	2	
	Sanitary towel (reusable)	1	2	
	Mattress pieces	1	2	
	Other (SPECIFY)	1	2	
Kama nyingine, (TAJA)	____________________			

Question	Response	Code	Skip
Kati ya njia unazotumia, je, unapendelea njia ipi sana?	Sanitary towel (disposable)	1	
	Tampon	2	
	Tissue paper/ other paper	3	
	Cotton wool	4	
	Old pieces of cloth	5	
	New peieces of cloth	6	
	Sanitary towel (reusable)	7	
	Mattress pieces	8	
	Other (SPECIFY)	98	

Sasa nitakuuliza maswali mengine zaidi kuhusu jinsi unavyoikabili hedhi yako shuleni, na jinsi unavyoikabili hedhi yako nyumbani.

Question	Response	AT SCHOOL YES	AT SCHOOL NO	AT HOME YES	AT HOME NO
Je, wewe hukabiliana vipi na uchungu au tumbo kuuma wakati wa hedhi yako? **[Check all that apply]**	Did not do anything	1	2	1	2
	Took medication	1	2	1	2
	Did not go to school	1	2	1	2
	Goes to hospital	1	2	1	2
	Used alternative pain medication. (SPECIFY_____)	1	2	1	2
	Does not have pain/cramps	1	2	1	2

Question	Response	AT SCHOOL	AT HOME	Skip
Je, wewe hubadilisha taulo za hedhi/vitu vingine? **[If never used pads ask about other materials used]**	YES	1	1	
	NO	2	2	
	N/A	97	97	
	**[IF NO FOR BOTH]**			**821**
Je, unaweza kubadilisha taulo za hedhi/vitu vingine kwa usiri mara ngapi? Unaweza kusema kila mara, wakati mwingine, nadra/mara moja moja, au huwezi kamwe?	Always	1	1	
	Sometimes	2	2	

Question	Response	At School	At Home	Skip
nadra/mara moja moja au huwezi kamwe?	Rarely	3	3	
	Never	4	4	
	You did not need to change	8	8	
Je, wewe hubadilishia wapi taulo ya hedhi/vitu vingine?		**AT SCHOOL**	**AT HOME**	
	Toilet	1	1	
	Classroom/room at home	2	2	
	Changed outside	3	3	
	Other (SPECIFY_____)	98	98	
	Not Applicable	97	97	
Je, wewe huweza kusafisha mikono baada ya kubadilisha?		**AT SCHOOL**	**AT HOME**	
	YES	1	1	
	NO	2	2	
	**[IF NO FOR BOTH]**			**819**
Je, wewe hutumia nini kunawa mikono baada ya kubadilisha?		**AT SCHOOL**	**AT HOME**	
		**YES NO**	**YES NO**	
	Soapy water	1 2	1 2	
	Soap	1 2	1 2	
	Water	1 2	1 2	
	Wet cloth	1 2	1 2	
	Dry cloth	1 2	1 2	
	Other	1 2	1 2	
	None	1 2	1 2	
Je, unaweza kutupa taulo za hedhi/vitu vingine vilivyotumiwa mara mingi kiasi gani? Unaweza kusema ni kila mara, wakati mwingine, nadra/mara moja moja au huwezi kamwe		**AT SCHOOL**	**AT HOME**	
	ALWAYS	1	2	
	SOMETIMES	2	2	
	NEVER	3	3	
	RARELY	4	4	
	N/A	8	8	
Je, wewe hutupa wapi taulo za hedhi/vitu vingine ulivyotumia? **[Check all that apply]**		**AT SCHOOL**	**AT HOME**	
		**YES NO**	**YES NO**	
	INSIDE LATRINE	1 2	1 2	
	LATRINE FLOOR	1 2	1 2	
	BIN	1 2	1 2	
	DROP IN FIELD	1 2	1 2	
	BURN IN FIELD	1 2	1 2	
	TAKE TO SCHOOL/HOME	1 2	1 2	
	DROP ON WAY TO SCHOOL/HOME	1 2	1 2	
	RIVER, LAKE, POND	1 2	1 2	
	OTHER (SPECIFY________)		98	
Je, katika miezi mitatu iliyopita, ulipata hedhi kila mwezi?	YES		1	
	NO		2	
Je, hedhi yako ya mwisho ilikuwa tarehe ngapi? **Note: First date when it started**	Date of period		[ ][ ][ ]	
Je, hedhi yako ya mwisho ilikuwa kwa siku ngapi?	Total days		[ ][ ]	
Wakati wa hedhi yako ya mwisho ulipata uchungu au kuumwa na tumbo?	YES		1	
	NO		2	
Je, damu imewahi kuvuja hadi kwa mwili wako au nguo ukiwa shuler	YES		1	
	NO		2	**FILTER**
Kama ndio, imefanyika mara mingi kiasi gani? **READ LIST**	KILA MARA (KILA HEDHI)		1	
	WAKATI MWINGINE		2	
	NADRA (MARA MOJA AU MBILI)		3	
	HAIJAWAHI KUFANYIKA		4	
Je, ulitumia nini ukiwa shule kuosha hiyo da	SOAPY WATER		1	
	SOAP AND WATER		2	
	WATER		3	
	WET CLOTH		4	
	DRY CLOTH		5	
	NONE		6	
	OTHER (SPECIFY)		98	
**[IF USED PADS IN Q808 OR Q811, SKIP 829]**				**829**
Je, umewahi kutumia taulo za hedhi?	YES		1	
	NO		2	**834**
Kwa wastani, wewe hutumia taulo ngapi za hedhi kila mwezi unapokuwa na hedhi?	Total number		[ ][ ]	

	Ikiwa uko na zingine zimebaki, wakati mwingine wewe hugawanya na mtu yeyote hizo taulo za hedhi?	YES 1<br>NO 2	 832
	Kama mnagawanya, wewe hugawana na nani?<br><br>**[Check all that apply]**	YES / NO<br>MOTHER 1 2<br>SISTER 1 2<br>CLASSMATES 1 2<br>FRIENDS WHO ARE NOT CLASSMATES 1 2<br>OTHER RELATIVE 1 2<br>TEACHER 1 2<br>OTHER (SPECIFY_________) 1 2	
	Kama ndio, taja	____	
	Je, unahisi kuwa unazo taulo za hedhi za kutosha kukabili hedhi yako vyema?	YES 1<br>NO 2	
	Kwa siku/masaa 24, wewe hubadilisha taulo za hedhi kama mara ngapi?	Once a day 1<br>Twice a day (Once every 12hours) 2<br>Three times a day (Once every 8hours) 3<br>More than three times a day / Once every 7hours or less 4<br>None 5<br>Other (SPECIFY_________) 98	
	Je, ulipokea taulo za hedhi shuleni muhula huu/muhula uliopita?	YES 1<br>NO 2	 838
	Kama ndio, ulipewa tu hizo taulo za hedhi ama ilibidi uombe?	Just given 1<br>Asked for them 2	
	Je, mara ya mwisho ulipokea taulo za hedhi shuleni, ulipokea pakiti tatu au zaidi, pakiti mbili, pakiti moja au chache kuliko pakiti moja?	Three or more packets 1<br>Two packets 2<br>One packet 3<br>Less than a packet 4	
	Katika muhula uliopita, ulipokea taulo za hedhi mara nyiingi kiasi gani shuleni?	Once or twice per term 1<br>Three or more times per term 2	

Niambie unafikiria taarifa zifuatazo ni kweli kwako kwa kiasi gani? Kwa kila taarifa, tafadhali niambie kama unakubali, unakataa ama hauna hakika.

	Unakubali	Unakataa	hauna haki	Don't know
**a.** Mimi huona aibu kwasababu ya mwili wangu wakati niko kwa hedh	1	2	3	88
**b.** Mimi huwa sijihisi vizuri mwilini wakati wa hedhi	1	2	3	88
**c.** Ni muhimu kuweka hedhi yangu siri kwa mtu yeyote	1	2	3	88
**d.** Najivunia kuwa na hedhi	1	2	3	88
**e.** Natamani nisingewahi kupata hedhi	1	2	3	88
**f.** Msichana anaweza kijuhusisha na michezo wakati wa hedh	1	2	3	88
**g.** Ni rahisi kuwa makini darasani wakati niko kwenye hedhi	1	2	3	88
**h.** Hedhi/damu ya mwezi ni ugonjwa	1	2	3	88
**i.** Msichana akiwa kwenye hedhi huwa sio msafi	1	2	3	88
**j.** Wavulana wanaweza kujua wakati wasichana wako kwenye hedhi	1	2	3	88
**k.** Kupata hedhi/damu ya mwezi ni jambo la kawaida kwa wasichana	1	2	3	88
**l.** Mtu hafai kuoga wakati wa hedhi	1	2	3	88
**m** Wasichana wote ambao wamewahi kupata hedhi wako katika hatari ya kupata mimba wakifanya ngono	1	2	3	88
**n**.Msichana hafai kuona haya akichafua nguo yake na damu akiwa shuleni wakati wa hedhi	1	2	3	88

	Je, kuna shughuli ambazo umekatazwa na mtu yeyote kufanya wakati uko kwenye hedhi?	Yes 1<br>No 2	 841
	Kama ndio, ni shughuli zipi?<br><br>**[CHECK ALL THAT APPLY]**	YES / NO<br>Going to school 1 2<br>Going to church/mosque 1 2<br>Going to market 1 2<br>Cooking for the family 1 2<br>Interacting with other family members 1 2<br>Interacting with neighbors 1 2<br>Interacting with men/boys 1 2<br>Playing games/sports 1 2	

		Fasting 1 2<br>Other (SPECIFY_____) 1 2	
	If you said other, please specify		
	Je, kuna shughuli ambazo wewe **huchagua** kutofanya wakati wa hedhi?	YES 1<br>NO 2	**843**
	Kama ndio, ni shughli zipi?<br><br>**[CHECK ALL THAT APPLY]**	YES NO<br>Going to school 1 2<br>Going to church/mosque 1 2<br>Going to market 1 2<br>Cooking for the family 1 2<br>Interacting with other family members 1 2<br>Interacting with neighbors 1 2<br>Interacting with men/boys 1 2<br>Playing games/sports 1 2<br>Other (SPECIFY) ________________________ 1 2<br>1 2	
	If you said other, please specify		
	Je, katika mwaka mmoja uliopita, kuna mtu ye yote ambaye amewahi kukutania kuhusu hedhi yako?	Yes<br>No	**901**
	Je, ni nani ambaye alikutania kuhusu hedhi yako?<br><br>**[CHECK ALL THAT APPLY**	**YES NO**<br>Brother 1 2<br>Sister 1 2<br>Other male relative 1 2<br>Other female relative 1 2<br>Male school mate 1 2<br>Female school mate 1 2<br>Other male friend 1 2<br>Other female friend 1 2<br>Teacher 1 2<br>Other (SPECIFY) ________________________ 1 2	
	If you said other, please specify		

SECTION 9: REPRODUCTIVE HEALTH KNOWLEDGE AND CONTRACEPTION

	**Tafadali niambie kama taarifa zifuatazo ni kweli au la/sio kweli?**<br><br>Je, uja uzito unaweza kutokea baada ya kubusu ama kukumbatiana?	TRUE 1<br>FALSE 2<br>DON'T KNOW 88<br>NO RESPONSE 96	
	Je, kunauwezekano kwa mwanamke kupata mimba **mara ya kwanza** anapofanya ngono?	TRUE 1<br>FALSE 2<br>DON'T KNOW 88<br>NO RESPONSE 96	
	Wasichana hupitia mabadiliko ya kubalehe. Tafadhali unaweza kuniambia ni mabadiliko gani ya mwili ambayo wasichana hupitia wakati wanapotoka kwa hali ya kuwa watoto na kubalehe?<br><br>**[CHECK ALL THAT APPLY]**	YES NO<br>Hair grows in different parts of body 1 2<br>Breasts develop 1 2<br>Acne appears 1 2<br>Menstruation starts 1 2<br>Rapid physical development 1 2<br>Voice Changes 1 2<br><br>Don't know 98	
	Kati ya wakati mmoja wa kuwa na hedhi hadi wakati mwingine je, kuna siku fulani ambapo mwanamke ana uwezekano mkubwa zaidi wa kupata mimba akifanya ngono?	YES 1<br>NO 2<br>DON'T KNOW 88	 **906**<br>**906**
	Je, wakati huo ni kabla tu ya hedhi yake kuanza, muda akiwa na hedhi, mara tu hedhi yake inapoisha, au wiki mbili baada ya hedhi yake?	JUST BEFORE HER PERIOD BEGINS 1<br>DURING HER PERIOD 2<br>RIGHT AFTER HER PERIOD ENDS 3<br>HALFWAY BETWEEN TWO PERIODS 4<br>OTHER(SPECIFY_______________) 98	
	**Tafadhali niambie kama taarifa zifuatazo ni kweli au la/sio kweli**<br>Kwa kawaida, hedhi/damu ya mwezi huja mara moja kwa mwezi<br><br>Kwa kawaida, hedhi/damu ya mwezi huja mara moja kwa mwezi	TRUE 1<br>FALSE 2<br>DON'T KNOW 88	
	Ni afya bora kwa mwanamke kuwa na utofauti wa unyevunyevu kwa sehemu za uke wakati mmoja wa kuwa na hedhi hadi ule mwingine	TRUE 1<br>FALSE 2<br>DON'T KNOW 88	

	Wanawake na wasichana wanaweza kupata maambukizi kwenye sehemu za siri /uke hata kama hawajawahi kufanya ngono	TRUE 1<br>FALSE 2<br>DON'T KNOW 88	
	Wanawake na wasichana wanaweza kuzuia maambukizi kwenye sehemu za siri /uke wakijipanguza sehemu za siri kwanzia mbele kwenda nyuma	TRUE 1<br>FALSE 2<br>DON'T KNOW 88	
	Je, ni mambo yapi msichana anaweza kukabiliana nayo akipata mimba akiwa kijana/tineja?<br><br>**[CHECK ALL THAT APPLY]**	YES NO<br>No problem 1 2<br>Health risk to mother 1 2<br>Health risk to baby 1 2<br>Education might get stopped 1 2<br>May not be able to continue working 1 2<br>May be forced to get married<br>Others(SPECIFY________) 98<br>Don't know 88	
	If you said other, please specify		
	Katika sheria ya Kenya, msichana anaweza kuolewa kuanzia miaka mingapi?	At age [__][__]<br>Don't Know 88	
	Je, ni nini ubaya wa msichana kuolewa mapema (kabla ya umri wa miaka 18) ?<br><br>**[CHECK ALL THAT APPLY]**	YES NO<br>Risk to child at birth 1 2<br>Risk to Mother at deliver 1 2<br>Immature and incapable of raising children 1 2<br>Immature and incapable of running household 1 2<br>Unable to complete education 1 2<br>Ill health/Depression 1 2<br>Other 1 2<br>Don't know 98	
	If you said other, please specify		
	Sasa nitakusomea baadhi ya taarifa. Tafadhali niambie kama unakubaliana ama haukubaliani nazo.		

	**UNAKUBALIANA**	**HAUKUBALIANI**	**DON'T KNOW**
**a**. Msichana ana haki ya kutafuta na kupokea habari kuhusu kujamiiana	1	2	88
**b**. Msichana/ mwananke ana haki ya kuchagua atakayemuoa	1	2	88
**c.** Msichana/mwanamke ana haki ya kuamua ikiwa atapata watoto ama la	1	2	88
**d.** Msichana ana haki ya kukataa kufanya ngono	1	2	88
e.Msichana ana haki ya kukataa ndoa ya lazima	1	2	88
**f.** Msichana/mwanamke ana haki ya kuamua wakati wa kupata watoto	1	2	88
**g**. Msichana wa umri wangu ana haki ya kujua kuhusu mpango wa uzazi na njia za kuzuia mimba	1	2	88
h. Msichana/mwanamke ana haki ya kusisitiza kutumia njia za kuzuia mimba/kupanga uzazi na mume/mpenzi wake.	1	2	88

Sasa ningependa kuongea kuhusu kupanga uzazi – njia au mbinu tofauti ambazo wenzi wanaweza kutumia ili kuchelewesha au kuzuia mimba.<br><br>Wewe umesikia kuhusu njia au mbinu zipi?<br><br>**[FOR METHODS NOT MENTIONED SPONTANEOUSLY, ASK:**<br>**Je umewahi kusikia kuhusu(MBINU YA)?]**<br><br>**If NO to 914 (a - i), skip to 915**		**915**<br>Je, umewahi kutumia [METHOD]?
**a.** KIDONGE/TEMBE: Wanawake wanaweza kumeza kidonge kila siku ili kuzuia mimba.	YES, SPONT 1<br>YES, RECOG 2<br>NO 3	YES 1<br>NO 2
**b.** IUD: Wanawake wanawekewa tanzi au pindi ndani yao na daktari au muuguzi.	YES, SPONT 1<br>YES, RECOG 2<br>NO 3	YES 1<br>NO 2
**c.** MASINDANO: Wanawake wanaweza kudungwa sindano na mhudumu wa afya inayowazuia wasipate mimba kwa muda wa mwezi mmoja au zaidi.	YES, SPONT 1<br>YES, RECOG 2<br>NO 3	YES 1<br>NO 2
**d.** VIDUDE VYA KUPACHIKWA: Wanawake wanaweza kupachikwa vidude kadhaa katika sehemu ya juu ya mikono	YES, SPONT 1<br>YES, RECOG 2	YES 1<br>NO 2

	yao na daktari au na muuguzi ambavyo vyaweza kuzuia mimba kwa mwaka mmoja au zaidi.	NO 3		
**e.**	KONDOMU YA WANAUME: Wanaume wanaweza kuweka ala ya mpira kwenye uume wao ulio wima kabla ya kufanya ngono.	YES, SPONT 1<br>YES, RECOG 2<br>NO 3	YES 1<br>NO 2	
**f.**	KONDOMU YA WANAWAKE: Wanawake wanaweza kuingiza katika uke wao, kabla ya kufanya ngono, mpira mwembamba usio na rangi.	YES, SPONT 1<br>YES, RECOG 2<br>NO 3	YES 1<br>NO 2	
**g.**	NJIA YA MWENDO: Kila mwezi ambapo mwanamke anatenda ngono yeye anaweza kuzuia mimba kwa kutofanya ngono katika siku za mwezi ambapo ana uwezekano mkubwa zaidi wa kupata mimba,	YES, SPONT 1<br>YES, RECOG 2<br>NO 3	YES 1<br>NO 2	
**h.**	UONDOA UUME: Wanaume wanaweza kuondoa uume wao kutoka katika uke kabla ya kutokwa shahawa.	YES, SPONT 1<br>YES, RECOG 2<br>NO 3	YES 1<br>NO 2	
**i.**	NJIA YA DHARURA YA KUDHIBITI UZAZI: Ikiwa hatua ya dharura, wanawake wanaweza kumeza vidonge hadi siku tatu baada ya kufanya ngono bila kujikinga ili kuzuia mimba. Vidonge hivyo huitwa pia "vidonge vya asubuhi ifuatayo."	YES, SPONT 1<br>YES, RECOG 2<br>NO 3	YES 1<br>NO 2	

**CHECK Q707. IF NEVER HAD SEX, SKIP TO 922**		
Je, kwa sasa unafanya chochote au unatumia mbinu yoyote ya kuchelewesha au kuzuia mimba?	NDIO 1<br>LA 2<br>NINA MIMBA KWA SASA 3	 **919**<br>**919**

Question		Response	YES	NO	Skip
Unatumia njia gani?<br><br>**[RECORD ALL MENTIONED]**	**a.**	TEMBE	1	2	
	**b.**	KITANZI (COIL)	1	2	
	**c.**	SINDANO	1	2	
	**d.**	VIPANDIKIZI (IMPLANTS)	1	2	
	**e.**	KONDOMU ZA WANAUME	1	2	
	**f.**	KONDOMU ZA WANAWAKE	1	2	
	**g.**	KUHESABU MASIKU/CALENDA	1	2	
	**h.**	KUMWAGA SHAHAWA NJE	1	2	**919**
	**i.**	KUNYONYESHA	1	2	**919**
	**j.**	TEMBE ZA DHARURA	1	2	
	**k.**	KUEPUKANA NA NGONO	1	2	**919**
	**i.**	INGINE (ELEZA________)	1	2	**919**

**[ASK IF ANSWER YES TO PILL, IUD, INJECTABLES, IMPLANTS, MALE CONDOM, EMERGENCY CONTRACEPTIVES OR FEMALE CONDOM IN 723]**<br><br>Ni wapi ulipokea [CURRENT METHOD] mara ya mwisho?	KLINIKI YA KIBINAFSI	1	
	HOSPITALI YA UMMA (SEREKALI)	2	
	KITUO CHA AFYA AU KLINIKI YA UMMA	3	
	ZAHANATI	4	
	DUKA LA DAWA	5	<u>**ALL**</u>
	HUDUMA TAMBA (ZA KUZUNGUKA)	6	
	VITUO VISIVYO VYA SEREKALI	7	**922**
	INGINE (ELEZA) …….. ______________________________	98	
Je, unajua mahali ambapo unaweza kupata njia ya kupanga uzazi?	NDIO	1	
	LA	2	**922**

Question	Response	YES	NO
Ni wapi hapo?<br>Kuna mahali pengine?<br><br>**[RECORD ALL MENTIONED]**	KLINIKI YA KIBINAFSI	1	2
	HOSPITALI YA UMMA (SEREKALI)	1	2
	KITUO CHA AFYA AU KLINIKI YA UMMA	1	2
	ZAHANATI	1	2
	DUKA LA DAWA	1	2
	HUDUMA TAMBA (ZA KUZUNGUKA)	1	2
	VITUO VISIVYO VYA SEREKALI	1	2
	INGINE (ELEZA) …….. ______________________________	1	2

Je, umewahi kuzungumzia na <u>**mama yako/mama mlezi**</u> kuhusu afya ya uzazi ama maswala ya kujamiiana au mapenzi	NO	1	**925**
	YES	2	
	N/A	9	**925**

Question	Response	**YES**	**NO**
Je, ni yapi kati ya mambo ya hali ya uzazi ama maswala ya kujamiiana au mapenzi yafuatayo ambayo umewahi kuzungumzia?<br><br>**[READ LIST; CHECK ALL THAT APPLY]**	MAWASILIANO	1	2
	HADHI/KUJIAMINI	1	2
	UJA UZITO	1	2
	KUBALEHE/JINSI WAVULANA NA WASICHANA WANAVY	1	2
	HIV/AIDS	1	2
	MAISHA YA NDOA/MAJUKUMU YA FAMILIA	1	2

[illegible]	HEDHI<br>MPANGO WA UZAZI<br>KUEPUKANA NA NGONO<br>AFYA NA USAFI<br>INGINE (ELEZA)	1 2<br>1 2<br><br>1 2<br>98	
Je, ni nini ingine ulizungumzia kuhusu afya ya uzazi ama maswala ya kujamiiana au mapenzi? (ELEZA)	______________________ NONE=	0	
Je, umewahi kuzungumzia na baba **yako/baba mlezi** kuhusu hali ya uzazi ama maswala ya kujamiiana au mapenzi miezi	NO<br>YES<br>N/A	1<br>2<br>9	**928**<br><br>**928**
Je, ni yapi kati ya mambo ya afya ya uzazi ama maswala ya kujamiiana au mapenzi yafuatayo ambayo umewahi kuzungumzia?<br><br>**[READ LIST; CHECK ALL THAT APPLY]**	MAWASILIANO<br>HADHI/KUJIAMINI<br>UJA UZITO<br>KUBALEHE/JINSI WAVULANA NA WASICHANA WANAVY<br>HIV/AIDS<br>MAISHA YA NDOA/MAJUKUMU YA FAMILIA<br>HEDHI<br>MPANGO WA UZAZI<br>KUEPUKANA NA NGONO<br>AFYA NA USAFI<br>INGINE (ELEZA)	**YES NO**<br>1 2<br>1 2<br>1 2<br>1 2<br>1 2<br>1 2<br>1 2<br>1 2<br><br>1 2<br>98	
Je, ni nini ingine ulizungumzia kuhusu afya ya uzazi ama maswala ya kujamiiana au mapenzi? (ELEZA)	______________________ NONE=	0	
Wakati mwingine wanawake hupata utoko/unyevu usio wa kawaida na wenye harufu mbaya katika sehemu za siri. Je, umeshawahi kupata utoko usiowakawaida na wenye harufu mbaya kwa sehemu zako za siri katika miezi kumi na mbili iliyopita?	YES<br>NO<br>DON'T KNOW	1<br>2<br>88	
Wakati mwingine wanawake hupata uchungu wakati wanakojoa pamoja na haja ya kukojoa mara kwa mara. Je, katika miezi kumi na mbili iliyopita, umepata uchungu unapokojoa pamoja na haja ya kukojoa mara kwa mara?	YES<br>NO<br>DON'T KNOW	1<br>2<br>88	
Wakati mwingine wanawake hupata vidonda kwenye sehemu za siri. Je, umeshawahi kuwa na vidonda kwa sehemu zako za siri katika miezi kumi na mbili iliyopita?	YES<br>NO<br>DON'T KNOW	1<br>2<br>88	
Check 922, 923 & 924. IF NO SYMPTOMS, GO TO FILTER			**FILTER**
Ulitafuta matibabu kwa hizi dalili?	YES<br>NO	1<br>2	**933**
Kwa nini hukutafuta matibabu kwa hizi dalili?	1 = Didn't know where to go<br>2 = No money for treatment<br>3 = Facility too far<br>4 = Symptoms cleared up by themselves<br>5 = Unfriendly staff<br>6 = Other (SPECIFY__ ____________________	1<br>2<br>3<br>4<br>5<br>98	**FILTER**<br>**FILTER**<br>**FILTER**<br>**FILTER**<br>**FILTER**<br>**FILTER**
Ni wapi ulitafuta ushauri ama matibabu mara ya mwisho ulipokuwa na hizi dalili?	PRIVATE CLINIC/HOSPITAL<br>PUBLIC HOSPITAL<br>PUBLIC HEALTH CENTER/CLINIC<br>DISPENSARY<br>CHEMIST/PHARMACY<br>MOBILE/OUTREACH SERVICE<br>NGO FACILITY<br>OTHER (SPECIFY) ….. ____________________________	1<br>2<br>3<br>4<br>5<br>6<br>7<br>98	

SECTION 10: PREGNANCY AND BIRTHS

QUESTIONS	RESPONSES		
**[IF NEVER HAD SEX) → Q1012]**			**1012**
Sasa ningependa kukuuliza kuhusu matukio ya kuzaa watoto ambao umekuwa nao muda wa maisha yako. Je, umewahi kuzaa mtoto?	YES<br>NO	1<br>2	**1003**
Je, umewahi kuzaa mvulana au msichana aliyezaliwa akiwa hai lakini aliyekufa baadaye?	YES<br>NO	1<br>2	 **1005**
Umezaa watoto mara ngapi kwa jumla maishani mwako?	TOTAL BIRTHS<br>[1-5]	[ ]	

Je wewe umewahi kupata mimba iliyotoka, au ambayo mtoto alizaliwa akiwa amekufa?	YES 1<br>NO 2	
Je, ulikuwa na miaka mingapi ulipozaa kwa mara ya kwanza?	Age [ ][ ]<br>Don't Know 88<br>[1-19]	
Je una mimba wakati huu?	YES 1<br>NO 2<br>UNSURE/DON'T KNOW 88	 **F3**<br>**F3**
Una mimba ya miezi mingapi?<br>**[RECORD NUMBER OF COMPLETED MONTHS]**	MONTHS [ ][ ]<br>[0-10]<br>DON'T KNOW 88	
**[IF <u>ALL</u> OF 1001, 1002, 1005, AND 1007 = NO → 1012]**		
Wakati wa kwanza kabisa ulipogundua una mimba, je, ulikuwa bado unaenda shuleni?	YES 1<br>NO 2	
Wakati wa kwanza kabisa ulipogundua una mimba, je, ulikuwa mseja, ulikuwa umechumbiwa au ulikuwa umefunga ndoa?	SINGLE 1<br>ENGAGED 2<br>MARRIED 3	
Wakati ulipopata mimba, je ulitaka kupata mimba, ulitaka kungoja mpaka baadaye, ama hukutaka hata kidogo kupata mimba?	THEN 1<br>ONE TO TWO YEARS LATER 2<br>THREE OR MORE YEARS LATER 3<br>NOT AT ALL 4	
Nyakati nyingine msichana hupata mimba asipotaka kuipata. Je, wewe umewahi kupata mimba usipotaka kuipata?	YES 1<br>NO 2	
Sasa niko na swali kuhusu maisha ya baadaye. Je, ungependa kupata mtoto/mtoto mwingine, ama ungependelea kutopata watoto/watoto wengine?	YES 1<br>NO 2<br>DON'T KNOW 88	 **1014**
Je, ungependa kupata mtoto/mtoto mwingine ukiwa na umri gan	Age [ ][ ]<br>Don't Know 88<br>[1-50]	
Je, ukipata mimba saa hizi, unaweza kufurahi kiasi gani? Unaweza kusema, kufurahi kabisa, kufurahi kiasi, ama huwezi kufurahi	VERY HAPPY 1<br>SOMEWHAT HAPPY 2<br>NOT HAPPY AT ALL 3	

SECTION 11: HIV/AIDS AND OTHER STIs

QUESTIONS	RESPONSES	
Sasa ningependa kuongea kuhusu jambo jingine. Je, umewahi kusikia kuhusu ugonjwa unaoitwa UKIMWI?	YES 1<br>NO 2	 **#REF!**
Je watu wanaweza kupunguza uwezekano wa kupatwa na virusi vya UKIMWI kwa kuwa na mwenzi wa ngono mmoja tu asiyeambukizwa ambaye hana wenzi wengine wa ngono?	YES 1<br>NO 2<br>DON'T KNOW 88	
Je watu wanaweza kupata virusi vya UKIMWI kutokana na kuumwa na mbu?	YES 1<br>NO 2<br>DON'T KNOW 88	
Je watu wanaweza kupunguza uwezekano wa kupatwa na virusi vya UKIMWI kwa kutumia kondomu kila mara wanapofanya ngono?	YES 1<br>NO 2<br>DON'T KNOW 88	
Je watu wanaweza kupata virusi vya UKIMWI kwa kushiriki chakula na mtu aliye na UKIMWI?	YES 1<br>NO 2<br>DON'T KNOW 88	
Je watu wanaweza kupunguza uwezekano wa kupatwa na virusi vya UKIMWI kwa kutofanya ngono kamwe?	YES 1<br>NO 2<br>DON'T KNOW 88	
Je watu wanaweza kupata virusi vya UKIMWI kwa sababu ya uchawi au kwa njia nyingine isiyo ya kawaida?	YES 1<br>NO 2<br>DON'T KNOW 88	
Je, mtu anayeonekana kuwa na afya njema anaweza kuwa na	YES 1	

virusi vya UKIMWI?	NO 2<br>DON'T KNOW 88	
Je virusi vinavyosababisha UKIMWI vyaweza kupitishwa kutoka kwa mama hadi kwa mtoto wake:<br><br>**a.** Wakati wa mimba?<br>**b.** Wakati wa kuzaa?<br>**c.** Kwa kunyonyesha?	YES NO DK<br>**a.** DURING PREGNANCY 1 2 88<br>**b.** DURING DELIVERY 1 2 88<br>**c.** BREASTFEEDING 1 2 88	
Je, wafikiri uwezekano wako wa kupata virusi vya UKIMWI ni kidogo, kiasi au juu au huna uwezekano wowote wa kupata?	LOW 1<br>MEDIUM 2<br>HIGH 3<br>NO RISK 4<br>ALREADY INFECTED 5<br>DON'T KNOW 88	 <br><br><br>**1115**
Sitaki kujua matokeo uliyopata, kwa hivyo usiniambie, lakini je, umewahi kupimwa kuona kama una virusi vya UKIMWI?	YES 1<br>NO 2	 **1115**
Ni lini mara ya mwisho ulipopimwa?	LESS THAN 6 MONTHS AGO 1<br>LESS THAN 12 MONTHS AGO 2<br>12 - 23 MONTHS AGO 3<br>2 OR MORE YEARS AGO 4	
Mara ya mwisho ulipopimwa, je ni wewe mwenyewe uliyeomba kupimwa, ulipewa nafasi ya kupimwa ukakubali au kupimwa kulitakikana?	ASKED FOR THE TEST 1<br>OFFERED AND ACCEPTED 2<br>REQUIRED 3	
Sitaki kujua matokeo uliyopata, kwa hivyo usiniambie, lakini je, ulipata matokeo ya kupimwa?	YES 1<br>NO 2	**1116**<br>**1116**
Je, unajua mahali ambapo watu wanaweza kwenda kupimwa kwa ajili ya virusi vya UKIMWI?	YES 1<br>NO 2	
Je, wewe binafsi wajua mtu aliye na au anayedhaniwa kuwa na virusi vya UKIMWI?	YES 1<br>NO 2	
**Sexually Transmitted Infections/Diseases**		
Mbali na virusi vya ukimwi, unajua magonjwa mengine yoyote ambayo yanaweza kusambazwa kupitia kwa ngono?	YES 1<br>NO 2<br>NO RESPONSE 96	 **1201**<br>**1201**
Je unaweza eleza dalili zozote ya magonjwa ya zinaa kwa wanawake?<br><br>**DO NOT READ THE LIST. MORE THAN ONE ANSWER IS POSSIBLE. CIRCLE ALL THAT APPLY**	YES NO<br>A. Abdominal pain 1 2<br>B. Genital discharge 1 2<br>C. Foul-smelling discharge 1 2<br>D. Burning pain on urination 1 2<br>E. Genital ulcers/sores 1 2<br>F. Genital warts 1 2<br>G. Swellings in the groin area 1 2<br>H. Itching 1 2<br>I. Other (SPECIFY_ _____________________ 1 2<br>J. Don't know 1 2	
Je, unaweza kunielezea dalili zo zote za ugonjwa wa zinaa ambazo hutokea kwa wanaume?<br><br>**DO NOT READ THE LIST. MORE THAN ONE ANSWER IS POSSIBLE. CIRCLE ALL THAT APPLY.**	YES NO<br>A. Discharge from the penis 1 2<br>B. Discharge from the anus 1 2<br>C. Burning pain on urination 1 2<br>D. Genital ulcers/sores 1 2<br>E. Swellings in the groin area 1 2<br>F. Can't retract the foreskin 1 2<br>G. Ulcer/sores on the anus 1 2<br>H. Ulcers/sores on the penis 1 2<br>I. Ulcers/sores in the throat 1 2<br>J. Lower abdominal pain 1 2<br>K. Other (SPECIFY)….. ________________ 1 2<br>L. Don't know 1 2	
Sasa nitakusomea baadhi ya taarifa kuhusu magonjwa ya zinaa na ningependa uniambie kama unakubali au haukubaliani nazo.		
Mtu anaweza kuwa na magonjwa ya zinaa bila kuonyesha dalili zozo	AGREE 1<br>DISAGREE 2<br>DON'T KNOW 88	
Magonjwa yote ya zinaa yanaweza tu kuambukizwa kupitia kwa ngor	AGREE 1<br>DISAGREE 2<br>DON'T KNOW 88	

Kondomu hukinga kutokana na magonjwa mengi ya zinaa	AGREE 1<br>DISAGREE 2<br>DON'T KNOW 88	
Je, umeshawahi kupata ama kushuku kwamba una ugonjwa wa zinaa katika miezi 6 iliyopita?	YES 1<br>NO 2<br>NO RESPONSE 96	 **1201**<br>**1201**
Ulitafuta matibabu kwasababu ya huo ugonjwa wa zinaa?	YES 1<br>NO 2	**1124**
Kwa nini hukutafuta matibabu kwa ugonjwa huo wa zinaa?	1 = Didn't know where to go 1<br>2 = No money for treatment 2<br>3 = Facility too far 3<br>4 = Symptoms cleared up by themselves 4<br>5 = Unfriendly staff 5<br>6 = Self medicated 6<br>7 = Other (SPECIFY__ ____________________ 98	**ALL**<br>**1201**
Ni wapi ulitafuta ushauri ama matibabu mara ya mwisho ulipokuwa na huo ugonjwa wa zinaa?	PRIVATE CLINIC 1<br>PUBLIC HOSPITAL 2<br>PUBLIC HEALTH CENTER/CLINIC 3<br>DISPENSARY 4<br>CHEMIST/PHARMACY 5<br>MOBILE/OUTREACH SERVICE 6<br>NGO FACILITY 7<br>OTHER (SPECIFY) ….. ______________________________ 98	

SECTION 12: GBV/DOMESTIC VIOLENCE

QUESTIONS	AGREE	DISAGREE	DON'T KNOW
Nitasoma mfululizo wa taarifa kuhusu ndoa kisha ninataka uniambie kama unakubaliana au haukubaliani nazo.			
**a.** Kama mwanamke kijana katika jamii hii angekuwa na umri wa miaka 25 bila kufunga ndoa, watu wangemheshimu.	1	2	88
**b.** Ni sawa kwa mwanamume kuipikia familia yake chakula.	1	2	88
**c.** Ndoa za wake wengi ni sehemu ya utamaduni wenu , basi zinapaswa kuendelea.	1	2	88
**d.** Ni bora zaidi kwa familia ya msichana kumpangia ndoa yake kuliko yeye mwenyewe kujichagulia.	1	2	88
**e.** Mwanamume asipompiga mke wake, yamaanisha hampendi.	1	2	88
**f.** Ni haki ya mwanamume kufanya ngono na mke wake wakati wowote anapotaka.	1	2	88
**g.** Mke anapaswa kuwa na uwezo wa kukataa kufanya ngono na mume wake. .	1	2	88<br>88
**h.** Mume na mke wakikosa kukubaliana kuhusu kupanga uzazi, maoni ya mume yanapaswa kutangulizwa.	1	2	88
**i.** Wasichana wa umri wangu wangekuwa washerati wangejua mahali pa kupata njia za kupanga uzazi	1	2	88

ATTITUDES TOWARDS GENDER VIOLENCE

Nitakuuliza ufikirie kuhusu hali kadhaa zinazohusisha mume na mke. Unapofikiria maswali hayo, tafadhali kumbuka kwamba tunakuuliza utoe maoni yako kuhusu kila moja ya hali hizo, wala si kuhusu mambo uliyoyapitia kibinafsi.

Nyakati nyingine mume anakasirishwa na mambo ambayo mke wake anafanya. Kwa maoni yako, je, mume ana haki ya kumchapa mke wake katika hali zifuatazo:

		YES	NO
**a.** akienda mahali bila kumweleza?	**a.** GOES OUT	1	2
**b.** akipuuza kuwatunza watoto?	**b.** NEGLECT CHILDREN	1	2
**c.** akibishana naye?	**c.** ARGUES	1	2
**d.** akikataa kufanya ngono na yeye?	**d.** REFUSES SEX	1	2
**e.** akiunguza chakula?	**e.** BURNS FOOD	1	2

Wakati mwingine watoto huwa wanajihisi kutokuwa salama ama	Often	1

kutishwa wakiwa shuleni, ama wakati wanaenda au kutoka shuleni. Je haya yamekufanyikia; mara kwa mara, wakati mwingine au kamwe?	Sometimes 2<br>Never 3<br>Don't know 88<br>Refused 96	 **1206**<br>**1206**<br>**1206**

		YES	NO
Je, ni nani huwa anakufanya uhisi kutokuwa salama ama kutishwa shuleni au unapoenda/ unapotoka shuleni (sio lazima useme jina lake)? **(CHECK ALL THAT APPLY)**	A male teacher	1	2
	A female teacher	1	2
	A male students	1	2
	A female students	1	2
	A male who is not a student at your school	1	2
	Other adults (SPECIFY)	1	2
	Other things (example: dogs, other animals)	1	2

Wengine: Unahisi unatisihwa na watu wagani wazima? ______________________________

Nyingine: Unahisi unatishwa na vitu gani zingine? ______________________________

		YES	NO
Unaweza kuniambia ni wapi huwa unajihisi kutokukuwa salama ama kutishwa ukiwa shuleni au unapoenda/unapotoka shuleni? **(CHECK ALL THAT APPLY)**	On the way to or from school	1	2
	In the school yard	1	2
	In the classroom	1	2
	In the school toilet or latrine	1	2
	Refused	1	2
	On the playing field	1	2
	Other (SPECIFY) ________________	1	2

Je, kuna mtu ambaye unaweza kumwendea ili upate usaidizi ukihisi umetishwa ama hauko salama ukiwa shuleni au unapoenda/unapotoka shuleni?	YES	1	
	NO	2	**1208**
	DON'T KNOW	88	**1208**

		YES	NO
Ni watu gani unaweza kuwaendea ili upate usaidizi ukihisi hauko salama ama umetishwa ukiwa shuleni au unapoenda/unapotoka shuleni? **(CHECK ALL THAT APPLY)**	Mother	1	2
	Father	1	2
	Brother	1	2
	Sister	1	2
	Male friend	1	2
	Female friend	1	2
	Boyfriend	1	2
	Female teacher	1	2
	Male teacher	1	2
	Female mentor or coach	1	2
	Male mentor or coach	1	2
	Religious leader	1	2
	Local admnistration	1	2
	Other relative	1	2
	Other nonrelative	1	2

Je, mwanamume yeyote amewahi kukufanyia yoyote kati ya mambo yafuatayo:
kama ndiyo, ni lini mara ya mwisho ilipotendeka: katika mwezi uliopita,
katika miezi sita iliiyopita, katika mwaka uliopita au zaidi ya mwaka uliopita?

	1208a		1208b	1208c			
	Ever Happen		Relation to Male	WHEN HAPPENED			
	YES	NO	Codes Below	IN THE LAST MONTH	IN LAST 6 MONTHS	IN LAST YEAR	MORE THAN 1 YEAR AGO
A. Kusema au kufanya jambo la kukuaibisha mbele ya watu wengine?	1	2		1	2	3	4
B. Kutisha kukuumiza au kuumiza mtu aliye karibu na wewe?	1	2		1	2	3	4
C. Kkukutukana au kukufanya uhisi vibaya juu yako mwenyewe?	1	2		1	2	3	4
D. Kukusukuma, kukutikisa au kukurushia kitu fulani?	1	2		1	2	3	4
E. Kukupiga kofi	1	2		1	2	3	4
F. Kukunja mkono wako ay kuvuta nywele zako	1	2		1	2	3	4
G. Kukupiga ngumi au kufanya jambo liwezalo kukuumiza	1	2		1	2	3	4
H. Kukupiga teke, kukuvuta, au kukuchapa	1	2		1	2	3	4
I. Kujaribu kukunyonga koo au kukuchoma kimakusudi	1	2		1	2	3	4
J. Kutisha kukushambulia na kisu au na silaha nyingine	1	2		1	2	3	4
K. Kukushambulia na silaha	1	2		1	2	3	4
L. Kukugusa kwa njia ya kingono (k.m. kukubusu, kukushika kwa nguvu, kukupapasa), usipotaka wafanye hivyo	1	2		1	2	3	4
M. Kujaribu kufanya ngono na wewe usipotaka akakosa kufaulu	1	2		1	2	3	4
N. Kukulazimisha kwa nguvu kufanya ngono naye hata kama	1	2		1	2	3	4
Kukulazimisha kufanya matendo ya kingono usipotaka	1	2		1	2	3	4

RELATIONSHIP CODES FOR 1208b
1 = Husband 6 = Neighbor 11 = Co-worker

	2 = Boyfriend<br>3 = Fiancé<br>4 = Friend, acquaintance<br>5 = Relative<br>7 = Fellow student<br>8 = Teacher<br>9 = Foreigner/tourist<br>10 = Employer<br>12 = Parent/Guardian<br>13 = House guard<br>14 = House boy<br>15 = Stranger/unknown person<br>16 = Other ……………..	
Unapofikiria yale ambayo umejionea miongoni mwa mambo tofauti ambayo tumekuwa tukizungumzia, je umewahi kujaribu kupata msaada ili wale watu waache au yule mtu aache kukufanyia mambo hayo tena?	YES 1<br>NO 2	**1212**
Umejaribu kupata msaada kutoka kwa nani?<br><br>Kutoka kwa yeyote mwingine?<br><br>**[RECORD ALL MENTIONED]**	YES / NO<br>OWN FAMILY 1 2<br>HUSBAND/PARTNER'S FAMILY 1 2<br>CURRENT/LAST/LATE HUSBAND/ PARTNER 1 2<br>CURRENT/FORMER BOYFRIEND 1 2<br>FRIEND 1 2<br>NEIGHBOR 1 2<br>RELIGIOUS LEADER 1 2<br>DOCTOR/MEDICAL PERSONNEL 1 2<br>POLICE 1 2<br>LAWYER 1 2<br>SOCIAL SERVICE ORGANIZATION 1 2<br>COMMUNITYLEADER/LOCAL ADMN L 1 2<br>TEACHER 1 2<br>OTHER ____________ 1 2<br>SPECIFY	
Je, umewahi kuambia mtu yeyote mwingine kuhusu jambo hilo?	YES 1<br>NO 2	
Kadri ujuavyo, je, babako/mlezi aliwahi mpiga mamako/mlezi?	YES 1<br>NO 2<br>DON'T KNOW 88	

SECTION 13: Decision Making

Je, taarifa zifuatazo ni kweli kwako mara ngapi?

	Mara kwa mara	Wakati mwingine	Kamwe/Nadra	Don't know	Refuse
**A.** Wazazi wangu ama walezi wangu huniuliza maoni yangu kuhusu mambo	1	3	3	88	99
**B.** Wazazi wangu ama walezi wangu husikiliza ninapotoa maoni yangu.	1	3	3	88	99
**C.** Marafiki zangu huwa wananiomba mawaidha wakiwa na shida	1	3	3	88	99
**D.** Nikiona kitu hakiko sawa shuleni ama mtaani nahisi kwamba ninaweza kumwambia mtu na atanisikiliza	1	3	3	88	99
**E.** Ninaweza kuzungumza nikiona mtu mwingine akiumizwa	1	3	3	88	99
**F.** Ninaweza kuomba msaada kutoka kwa watu wazima nikiuhitaji	1	3	3	88	99

Je, unaweza kutoa maamuzi yafuatayo mwenyewe mara mingi kiasi gani bila mtu r

	Kamwe	Wakati mwingine	Kamwe/Nadra	Don't know	Refuse
**A.** Ni nguo gani utavaa wakati hauko shuleni/kazini	1	3	3	88	99
**B.** Ni nini utafanya wakati wako wa mapumziko	1	3	3	88	99
**C.** Ni nini utakula wakati hauko nyumbani	1	3	3	88	99
**D.** Kiasi cha elimu utakachopata (k.m. kumaliza shule ya upili, kwenda chuo kikuu)	1	3	3	88	99
**E.** Ni nani unaweza kuwa marafiki naye	1	3	3	88	99
**F.** Kuamu mwenyewe wakati wa kuolewa	1	3	3	88	99
**G.** Kuamua mwenyewe mtu atakay kuoa	1	3	3	88	99

Je, unadhani kwamba utaweza kuamua mwenyewe wakati wa kuolewa?<br><br>**IF MARRIED, ASK:** Je, uliweza kuamua mwenyewe wakati wa kuolewa?	YES 1<br>NO 2<br>DON'T KNOW 88	
Je, unadhani kwamba utaweza kuamua mwenyewe mtu atakaye kuoa?<br><br>**IF MARRIED, ASK:** Je, uliweza kuamua mwenyewe mtu aliye kuoa?	YES 1<br>NO 2<br>DON'T KNOW 88	

END CAPI: If you would like to discuss any of the questions or issues raised in this survey, please feel free to discuss this with the interviewer. The interviewer can provide information to contact a social welfare officer to discuss your concerns, or can contact the social welfare officer for you. Thank you very much for participating in this important study.
